# Integrated Network Pharmacology and Gut Microbiota Analysis to Explore the Mechanism of Sijunzi Decoction Involved in Alleviating Airway Inflammation in a Mouse Model of Asthma

**DOI:** 10.1155/2023/1130893

**Published:** 2023-01-03

**Authors:** Wenqing Jia, Chengling Xu, Tong Zhao, Qiuyang Fan, Bo Qiao, Yueying Wu, Jiali Yuan, Jing Chen

**Affiliations:** ^1^School of Basic Medical Science, Yunnan University of Chinese Medicine, Kunming, Yunnan, China; ^2^School of Chinese Medical Science, Hunan University of Chinese Medicine, Changsha, Hunan, China; ^3^College of First Clinical Medical Science, Yunnan University of Chinese Medicine, Kunming, Yunnan, China; ^4^Yunnan Key Laboratory of Molecular Biology of Traditional Chinese Medicine, Kunming, Yunnan, China

## Abstract

**Background:**

Asthma is a chronic inflammatory disease of the airways with recurrent attacks, which seriously affects the patients' quality of life and even threatens their lives. The disease can even threaten the lives of patients. Sijunzi decoction (SJZD), a classical Chinese medicine formula with a long history of administration, is a basic formula used for the treatment of asthma and demonstrates remarkable efficacy. However, the underlying mechanism has not been elucidated.

**Materials and Methods:**

We aimed to integrate network pharmacology and intestinal flora sequencing analysis to study the mechanism of SJZD in the treatment of allergic asthmatic mice. The active compounds of SJZD and their asthma-related targets were predicted by various databases. We performed Gene Ontology (GO) and Kyoto Encyclopedia of Genes and Genomes (KEGG) analyses to identify potentially relevant pathways for target genes. Furthermore, the active compound-target and target-signaling pathway network maps were constructed by using Cytoscape 3.8.2. These results were combined with those of the intestinal flora sequencing analysis to study the influence of SJZD on airway inflammation in allergic asthmatic mice.

**Result:**

We obtained 137 active compounds from SJZD and associated them with 1445 asthma-related targets acquired from the databases. A total of 109 common targets were identified. We visualized active compound-target and target-signaling pathway network maps. The pathological analysis and inflammation score results suggested that SJZD could alleviate airway inflammation in asthmatic mice. Sequencing analysis of intestinal flora showed that SJZD could increase the relevant abundance of beneficial bacterial genus and maintain the balance of the intestinal flora. The core toll-like receptor (TLR) signaling pathway was identified based on network pharmacology analysis, and the important role TLRs play in intestinal flora and organismal immunity was also recognized. The analysis of the correlation between environmental factors and intestinal flora revealed that beneficial bacterial genera were negatively correlated with TLR2 and positively correlated with the TLR7 expression. Furthermore, they were positively correlated with IFN-*γ* and IL-10 levels and negatively correlated with IL-4 and IL-17 levels.

**Conclusion:**

SJZD alleviated the airway inflammation state in asthmatic mice. The findings suggest that increasing the relevant abundance of beneficial intestinal bacteria in mice with asthma, regulating intestinal flora, interfering with the level of TLR2 and TLR7 expression to adjust the secretion of inflammatory factors, and alleviating asthmatic airway inflammation may be the possible mechanism involved in the treatment of asthma by SJZD, providing a basis for further studies on SJZD.

## 1. Introduction

Asthma is a disease of chronic airway inflammation, involving a variety of inflammatory cells like eosinophils, macrophages, mast cells, T lymphocytes, and epithelial cells, characterized by airway inflammation, airway hyperresponsiveness, and abnormal mucus secretion. Clinical manifestations include chest tightness, wheezing, shortness of breath, and/or cough, which are often repeatedly observed [1]. Statistical analysis indicates that approximately 330 million individuals have been diagnosed with asthma globally. The associated incidence and mortality are significant, and the use of antiasthma medication in adults is increasing [2, 3]. Furthermore, recurrent asthma attacks with a progressive decline in the lung function increase morbidity and mortality rates, imposing a significant economic burden on patients and society. Many studies have focused on immune imbalance as a factor in the development and progression of asthma, and gastrointestinal microbes have an important effect on maintaining local [4] and systemic imbalance of immunity in humans [5, 6]. Dysbiosis of gut microbiota can lead to colonization and proliferation of potentially pathogenic bacteria, affecting immune homeostasis and inducing the production of inflammatory factors, and this is highly associated with the onset and development of asthma [7–9]. Significant differences have been observed in gut microbial composition between patients with asthma and healthy individuals [10]. Hence, intestinal flora has a significant role in asthma. Microbial differences in asthmatics have an impact on the production of mucins and inflammatory factors, for example, interleukin (IL)-4 and IL-5 [11], and alterations in gut microbial patterns are closely related to the degree of sensitization and inflammation in asthma [12].

Chinese traditional medicine has been practiced for thousands of years with a sound theoretical foundation and rich clinical experience. It is widely recognized for its efficacy and few side effects. Sijunzi decoction (SJZD), recorded in the Chinese ancient medicine book “Prescriptions of the Bureau of Taiping People's Welfare Pharmacy,” has effects of improving qi and strengthening the spleen and is widely used in classic formulations. The main pathogenesis of asthma, as considered in traditional Chinese medicine, is the deficient functioning of both lungs and spleen. “Treatise on the spleen and stomach” states that “the lung is the most affected by spleen and stomach weakness.” It is considered that deficient functioning of the spleen leads to the inability to transport the essence of water and grain, resulting in the accumulation of fluid and phlegm, along with the inability to nourish the lungs. Hence, the lungs cannot resist external pathogens, resulting in asthma [13]. Therefore, deficient splenic functioning is the core pathogenic mechanism underlying asthma, and the treatment should focus on strengthening the spleen [14]. SJZD is used as a basic formula for the treatment of asthma; it supplements qi and strengthens the spleen, and the curative effect is remarkable [15, 16]. Modern medicine has found that SJZD has shown significant effects in increasing the intestinal flora variety and abundance [17, 18], regulating imbalances in immune function [19], and ameliorating inflammatory diseases in the lungs [20, 21]. However, the components of SJZD are complex, and its potential mechanisms for the treatment of asthma have not yet been clarified. Therefore, the exploration of the therapeutic effects of SJZD in asthma possibly provides a direction in the development of clinical treatment strategies.

**Figure 1:**
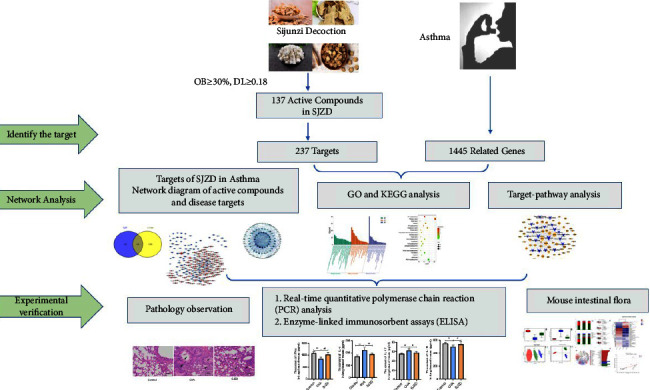
Schematic research strategy of this experiment.

In recent years, network pharmacology has been recognized as a useful instrument to expedite the process of modernizing Chinese traditional medicine. Through high-throughput data analysis, computerized virtual screening, and network prediction technology, network pharmacology uses multiple Chinese medicine databases to analyze Chinese medicine formulations, screen the active components of Chinese medicine, and predict the potential targets and pathways for the treatment of diseases [22]. On the basis of network pharmacology, the “active compound-target-pathway network” of traditional Chinese traditional medicine formulas was constructed, and the potential mechanism of Chinese herbal medicine for disease treatment was studied. Through integrated network pharmacology, many researchers have found that Danlong Dingchuan decoction can inhibit the onset of asthma by reducing inflammatory factors in the lung tissues [23]. Guizhi decoction can regulate macrophages to exert anti-inflammatory effects and participate in immune regulation to treat asthma [24]. The aforementioned findings indicate that the systematic and holistic characteristics of network pharmacology coincide with the overall regulation of multitargets and multipathways of Chinese traditional medicine formulas. Hence, this method can be used to scientifically predict the potential mechanisms associated with Chinese medicine for treating diseases and develop novel strategies for the treatment of asthma. Hence, in our present study, by using the analysis of intestinal flora in combination with network pharmacology, we explored the efficacy and mechanism of effect with SJZD on allergic asthma. The detailed research strategy is shown in Figure 1.

## 2. Materials and Methods

### 2.1. Collection and Selection of Relevant Targets for SJZD and Asthma

SJZD comprises ginseng, *Atractylodes macrocephala*, *Poria cocos,* and licorice. All active compounds are collected from the pharmacological database of the Chinese traditional medicine system (https://tcmspw.com/tcmsp.php), the encyclopedia of traditional Chinese medicine (https://www.tcmip.cn/ETCM), and relevant literature [25–29]. Pharmacology Database and Analysis Platform (TCMSP) takes oral bioavailability (OB) ≥30% and drug similarity (DL) ≥0.18 as thresholds for screening important active compounds. The corresponding targets of SJZD active components were also obtained from the TCMSP database. “Asthma” was searched in the Gene Cards (http://www.genecards.org/), DrugBank (http://www.https://go.drugbank.com/), TTD(http://db.idrblab.net/ttd/), and OMIM (https://www.ncbi.nlm.nih.gov/omim/) databases to identify potential therapeutic targets associated with asthma. The UniProt database (https://www.uniprot.org/) was used to standardize gene information and further eliminate repeated and false-positive genes to select common targets.

### 2.2. Construction and Analysis of SJZD and Asthma Disease Network

The intersection targets of the active ingredients of SJZD relative to the target genes and asthma-related genes were derived using the R language plug-in a Venn diagram. The construction of active compound-target maps for Chinese medicine was performed using the Cytoscape software. The String database was used to generate common target protein-protein interaction (PPI) network (https://string-db.org/cgi/input.pl) and calculate the node degree of PPI with the network analyzer tool function of String database. Using the DAVID database (https://david.ncifcrf.gov/), Gene Ontology (GO) and Kyoto Encyclopedia of Genes and Genomes (KEGG) enrichment analyses were performed at *P* < 0.05, and the enrichment results were integrated and visualized. Finally, the relationship between the selected former 20 pathways and the intersecting targets was visually analyzed by constructing a target-pathway network using Cytoscape software.

### 2.3. Experimental Validation

#### 2.3.1. Animals and Environment

A total of 24 six-to-eight-week-old SPF female BALB/c mice, weighing 20 ± 2 g, were purchased from Chengdu Dashuo Experimental Animal Co. Ltd (Animal license number SCXK (Sichuan) 2020-030). The experiments followed the “Guideline for the Care and Use of Laboratory Animals” published by the National Institutes of Health. The experiments on all animals were authorized by the Yunnan University of Traditional Chinese Medicine Animal Ethics Laboratory Committee (R-06202085). The experiment was initiated after seven days of adaptation, with free access to food and water for mice. The temperature was maintained at 22–25°C with 60% relative humidity.

#### 2.3.2. Establishment of Asthma Model and Treatment

**Figure 2:**
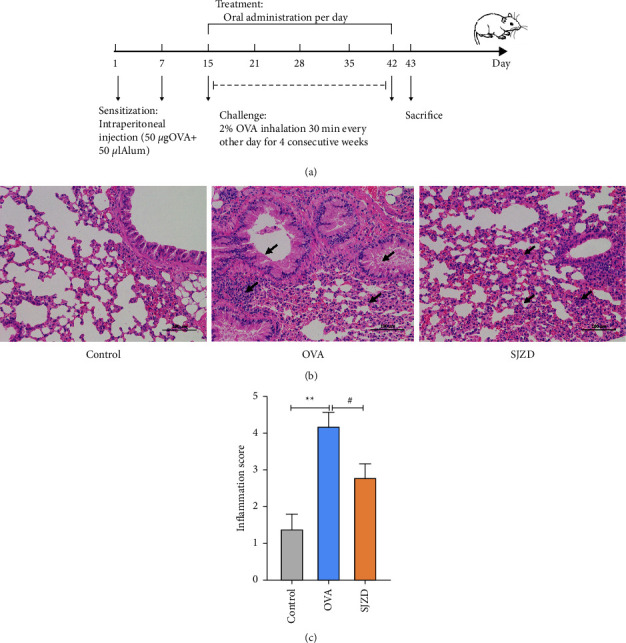
(a) Flowchart of the development of ovalbumin (OVA)-induced asthma mouse model and treatment. In the control group, animals were given saline instead of OVA. (b, c) Hematoxylin and eosin (H&E) staining and inflammation scores of lung histopathology. Magnification, 200x. Data are presented as mean ± SD by one-way ANOVA. *n* = five mice/group. ^*∗∗*^*P* < 0.01 vs control group. ^#^*P* < 0.05 vs OVA group.

We used an intraperitoneal injection of nebulized ovalbumin (OVA) to establish a mouse asthma model. We evaluated the success of the model by observing lung histopathological changes of the lungs and scoring the mice using the lung tissue inflammation scoring criteria presented by Underwood et al. [30]. Bronchial and vascular inflammatory cell infiltration, wall thickening, and degree of tracheal secretions were scored from 0 to 5. After one week of adaptation, the mice were divided into three groups according to the randomization principle (*n* = 8 in each group): control group (Control), OVA group (OVA), and SJZD group. During the sensitization phase on days one and seven, mice (except those in the control group) were injected intraperitoneally with 0.2 ml of 0.9% saline containing 50 *μ*g of OVA and 50 *μ*L of Imject Alum (Thermo Scientific, US). Nebulized excitation was initiated on day 15 with 2% OVA for 30 min and then performed every alternate day. The SJZD group was gavaged with SJZD (4.3 g/kg) one hour before excitation. SJZD was administered once daily for 28 days, and samples were collected on day 43 (Figure 2(a)). The control group was administered saline throughout the experiment.

The dose of SJZD was converted based on the clinical adult dose. The dose for mice was calculated according to the equivalent dose conversion ratio between human and mouse body surface area, which was 4.3 g/kg/d according to the conversion method of “Conversion of dose for experimental animals and human” listed in the Appendix of 《Methodology of Pharmacological Research on Chinese Medicine》 [31].

SJZD was prepared according to the “Pharmacological Experimental Methodology of Traditional Chinese Medicine” [32]. A total of 33 g of raw herbs of SJZD were steeped in 600 ml of water for 30 min and boiled and decocted for 1 h. The mixture was filtered via eight layers of a surgical gauze. The dregs were poured into 600 ml of water again, and the aforementioned operation was repeated twice. The collected solvents were mixed, and the volume was concentrated to 77 ml by a rotary evaporator to obtain SJZD preparation with a concentration of 0.43 g/ml.

#### 2.3.3. 16S rDNA Sequencing of Intestinal Contents

Stool samples were collected from the colon. FastDNA® Spin Kit (MP Biomedicals, GA, U.S.) was used to extract total DNA following the manufacturer's protocol, and the quality of the extracted DNA was analyzed using a 1% agarose gel. The concentration and purity of DNA were measured with NanoDrop2000. The highly variable region V3-V4 of the 16S rRNA gene of bacteria was amplified using primers 338F (5′-ACTCCTACGGGAGGCAGCAG-3′) and 806R (5′- GGACTACHVGGGTWTCTAAT-3′). The polymerase chain reaction (PCR) was completed using a thermal cycling PCR system (GeneAmp 9700, ABI, USA) with the following procedure: denaturation at 95°C for 3 min, and then denaturation at 95°C for 30 s, annealing at 55°C for 30 s, extension at 72°C for 45 s, and final extension at 72°C for 10 min, for 27 cycles. AxyPrep DNA Gel Extraction Kit (Axygen Biosciences, Union City, CA, USA) was used to purify the PCR products and quantitate them using a Quantus™ fluorometer (Promega, USA).

Sequencing was performed on the Illumina Miseq PE300 platform (Shanghai Meiji Biomedical Technology Co. Ltd.). OTU clustering of sequences was performed using UPARSE software and chimeras were eliminated based on 97% similarity (version 7.0). The OTU sequences were compared with the Silva database and analyzed for alpha diversity using Mothur (version 1.30) software. For beta diversity analysis, a principal coordinate analysis (PCoA) with the Bray–Curtis distance matrices was performed and visualized by evaluating the Shannon and Simpson indices, PCoA, and PLS-DA using the R software. To observe differences in microbial diversity among samples, a linear discriminant analysis (LDA) effect size (LEfSe) method was used for significance testing, setting the log LDA score at 2.0. In addition, the Spearman rank correlation between environmental variables and altered microbial genera was analyzed, the relationship between species and environmental variables was mined by correlation heat map and linear mode. Data were analyzed using the online Majorbio Bio-Pharm Cloud Platform (http://www.majorbio.com/).

#### 2.3.4. Histopathological Analysis of the Lung

The lung tissue was frozen and inserted in 4% paraformaldehyde and paraffin, stained with hematoxylin and eosin (H&E), and subjected to standard histopathological examination. H&E staining was mainly used to evaluate peribronchial inflammation. Pulmonary tissue inflammation scoring criteria were referenced from the study by Underwood et al. [30]. The samples were scored from 0 to 5 according to the degree of tracheal and perivascular inflammatory cell infiltration, wall thickening, and degree of tracheal secretions.

#### 2.3.5. Enzyme-Linked Immunosorbent Assay (ELISA)

The ELISA kit was used to measure the concentrations of interferon (IFN)-*γ*, IL-4, IL-17, and IL-10 in the mouse lung tissue homogenates. For the preparation of lung tissue homogenates, 50 *μ*g of lung tissue was added to 500 *μ*l of PBS and ground using a tissue grinder, followed by centrifugation at 2500 r/s for 20 min, and subsequently, the supernatant was collected. The ELISA kit was left to stand at room temperature for 20 minutes. In the standard wells, we added 50 *μ*l of standards of different concentrations. In the sample wells, 40 *μ*l of sample dilution and 10 *μ*l of sample to be tested were added (samples were diluted 5 times). Finally, adding 100 *μ*l of horseradish peroxidase-labeled antibody (except blank wells), then incubating at 37°C for 1 h. After removing the liquid, the samples were washed 5 times; then, 50 *μ*l of substrate A and B solutions were added. After incubation for 15 min at 37°C, a termination solution was added. The samples were analyzed at 450 nm within 15 minutes, and the concentrations were calculated.

#### 2.3.6. Real-Time Quantitative PCR Analysis

Following the extraction of total RNA with Trizol according to the manufacturer's instructions, cDNA was obtained by reverse transcription using the cDNA kit Hifair III 1st Strand cDNA Synthesis SuperMix (Yeasen Biotechnology Co., Ltd., Shanghai, China). Quantitative real-time PCR was performed using Hieff® qPCR SYBR Green Master Mix (Yeasen Biotechnology Co., Ltd., Shanghai, China). Initial denaturation was conducted at 95°C for 5 minutes, and then, 45 cycles of PCR, with 95°C for 10 seconds and 60°C for 30 seconds were conducted as cycling conditions. The primer sequences for PCR are shown in Table 1. The 2^−ΔΔ*Ct*^ method was used to calculate the relative expression of genes.

#### 2.3.7. Statistical Analysis

Statistics analysis was conducted by using SPSS 23.0. One-way ANOVA was used for data analysis based on normality tests and homogeneity of variances. Statistical analysis was performed using a nonparametric test if the data were not normally distributed or if the variance was not homogeneous. Values with *P* < 0.05 were considered statistically significant.

## 3. Results

### 3.1. Targets of SJZD Associated with Asthma

**Figure 3:**
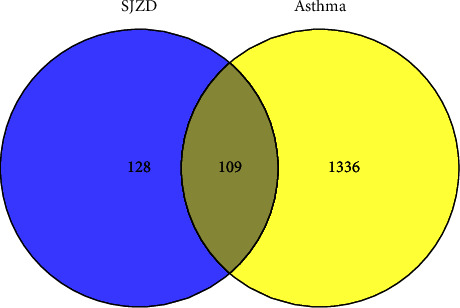
Venn diagram of the active compound-targets of SJZD and asthma-related targets.

**Figure 4:**
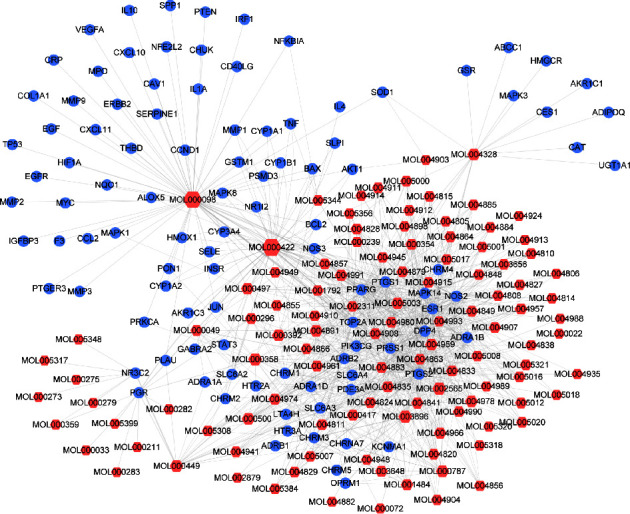
Target network of SZJD in asthma. The blue nodes represent potential targets in asthma. The red hexagonal nodes represent the active compounds screened in SZJD. The sizes of the nodes are proportional to the degree of association.

In this study, 137 compounds were obtained through database and literature research (Supplementary Table 1), including kaempferol, quercetin, and beta-sitosterol. The top 10 compounds were listed according to the degree value (Table 2). A total of 1445 genes associated with asthma were obtained from the four databases of DrugBank, GeneCards, TTD, and OMIM (Supplementary Table 2). A Venn diagram demonstrated an overlap of 109 genes related to both asthma and SJZD **(**Supplementary Table 3; Figure 3). The Cytoscape software was used to perform a network visual analysis of the data and construct a network of SJZD targets in asthma (Figure 4).

### 3.2. PPI Network Construction

**Figure 5:**
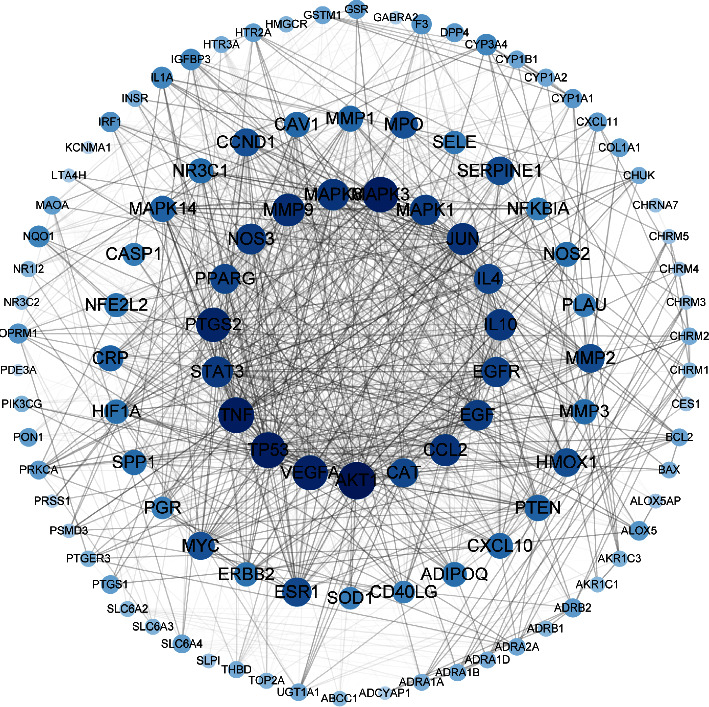
Network of protein-protein interactions (PPI). The circle size and color darkness indicate the importance of the node in the network.

The gene data corresponding to 109 targets were uploaded to the String database, and “Homo sapiens” was selected as the species to construct the PPI network and evaluate the key nodes. Further analysis of the correlation between these 109 targets and asthma was performed using the Cytoscape software (Figure 5). IL-4, IL-10, mitogen-associated protein kinase (MAPK)1, tumor necrosis factor (TNF), vascular endothelial growth factor (VEGF)A, AKT, matrix metalloproteinase (MMP)9, MAPK8, and prostaglandin-endoperoxide synthase (PTGS)2 were found to be the core targets in asthma prevention and control mediated by SJZD.

### 3.3. Target-Pathway Network Analysis

**Figure 6:**
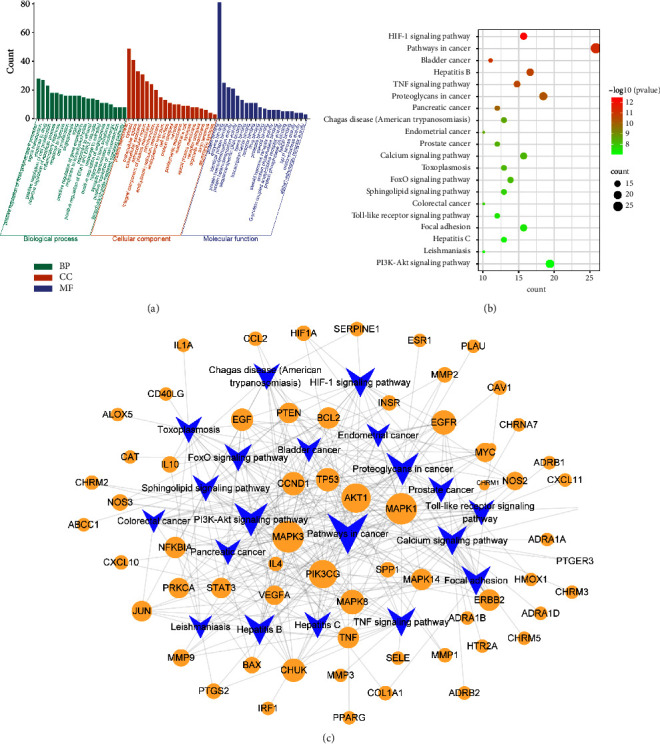
(a) Gene Ontology (GO) analysis of the top 20 target genes associated with the effect of SJZD on asthma. (b) Kyoto Encyclopedia of Genes and Genomes (KEGG) analysis of the top 20 target gene enrichment signaling pathways of SJZD associated with asthma target genes. (c) SJZD target-pathway network diagram. The blue diamond triangles represent signaling pathways, and the yellow nodes represent target genes.

GO and KEGG enrichment analyses were performed for 109 targets derived using the DAVID database. The results showed enrichment with respect to 354 biological processes (BPs), 48 cellular components (CCs), and 96 molecular functions (MF) (Supplementary Table 4). The top 20 most significantly enriched items (*P* < 0.05) were selected from BPs, CCs, and MFs. The main BPs involved responses to lipopolysaccharide, inflammation, toxic substances, lipopolysaccharide-mediated signaling pathway, positive regulation of ERK1 and ERK2 cascade, and cell proliferation (Figures 6(a) and 6(b)). SJZD may regulate the excessive secretion of inflammatory factors via enzyme binding, receptor binding, and protein-binding transcription factor binding in the extracellular space and plasma membrane. To further study the intervention pathways of SJZD in asthma, KEGG pathway analysis was performed. A total of 117 pathways were obtained (Supplementary Table 5), with the top 20 most significant pathways including the hypoxia-inducible factor-(HIF)1 signaling pathway, TNF signaling pathway, toll-like receptor (TLR) signaling pathway, and phosphatidylinositol-3-kinase (PI3K)-Akt signaling pathway (Figure 6(b)). To characterize the dynamics of gene targets at the cellular and molecular levels and their degree of importance in the network more clearly, a target-pathway network diagram was constructed using Cytoscape with the top 20 significantly enriched signaling pathways (Figure 6(c)).

### 3.4. Experimental Validation

#### 3.4.1. Effect of SJZD on the Pathological Tissues of Asthmatic Mice

In the control group, the pathological changes in the lung tissue were not obvious. The bronchial lumen was regular, the airway mucosal epithelium was intact, and no inflammatory cell infiltration around the bronchus and blood vessels was observed. The alveolar morphology was regular, and the thickening of the tracheal smooth muscle was not observed. In the OVA group, the mucus plugs in the lumen were increased, and a large number of inflammatory cells were observed. The bronchial tube wall was significantly thickened at the base, and some fusion and different degrees of fracture of the alveoli were visible. In the SJZD group, the inflammatory cell infiltrate was reduced, the epithelial structure was more intact, and mucus secretion was significantly reduced (Figures 2(b) and 2(c)).

#### 3.4.2. SJZD Regulates the Inflammatory Response in Asthmatic Mice

**Figure 7:**
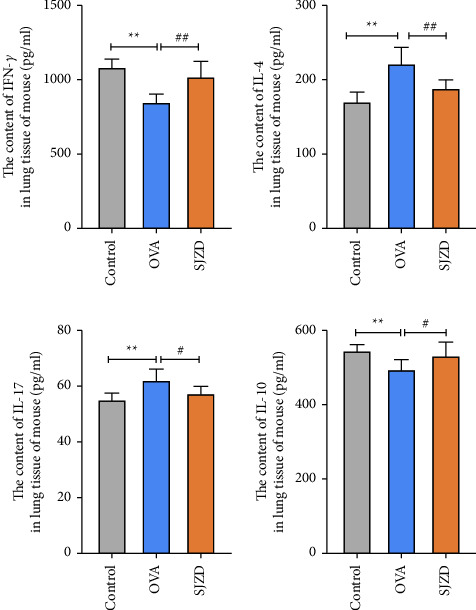
Effects of SJZD on the expression of IFN-*γ*, IL-4, IL-17, and IL-10 in the lung tissues of asthmatic mice. The graphs indicate the actual concentrations of the samples. Data are presented as mean ± SD derived by one-way ANOVA. n = six mice/group. ^*∗∗*^*P* < 0.01 vs control group. ^#^*P* < 0.05 vs OVA group.

The inflammatory response plays a vital role in the onset and development of asthma. IL-4, IL-17, IFN-*γ*, and IL-10 are the representative secretion factors of Th1, Th2, Th17, and Treg cells in asthma. IL-4 and IL-17 are critical inflammatory factors in asthma, and IFN-*γ* and IL-10 are essential anti-inflammatory factors. We found that compared with the control group, the expression of IFN-*γ* and IL-10 was significantly reduced (*P* < 0.01), and the expression of IL-4 and IL-17 was significantly increased (*P* < 0.01) in the lung tissues of the OVA group. Compared with the OVA group, the expression of IFN-*γ* and IL-10 was significantly increased (*P* < 0.05), and the expression of IL-4 and IL-17 was significantly decreased (*P* < 0.01) in the lung tissue of the SJZD group. The results showed that SJZD significantly improved the inflammatory response in asthmatic mice (Figure 7).

#### 3.4.3. SJZD Improves the Expression of TLR2 and TLR7 in Asthmatic Mice

**Figure 8:**
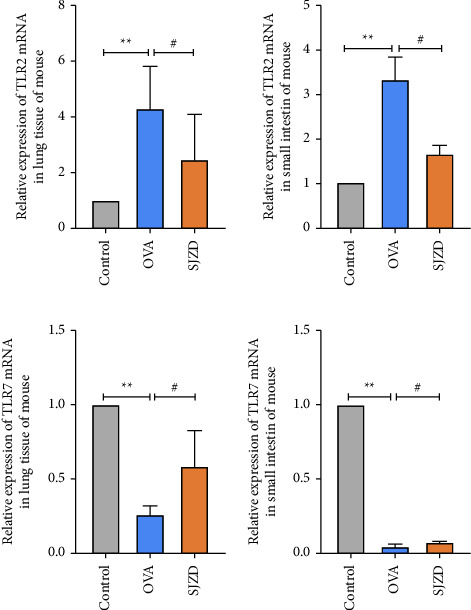
Effect of SJZD on mRNA expression levels of TLR2 and TLR7 in the lung and intestinal tissues of asthmatic mice. The mRNA levels were normalized to those of *β*-actin. Data are represented as mean ± SD and derived by one-way ANOVA. n = four mice/group. ^*∗∗*^*P* < 0.01 vs control group. ^#^*P* < 0.05 vs OVA group, ^##^*P* < 0.05 vs OVA group.

The TLR signaling pathway was identified as a target of SJZD in asthma treatment through predictive network pharmacology analysis. TLRs are transmembrane receptors that recognize pathogenic microorganisms. They can recognize molecular patterns related to various pathogenic microorganisms and trigger inflammatory responses. By detecting the expression levels of TLR2 and TLR7 mRNA, we found that compared with the control group, the expression level of TLR2 mRNA was significantly increased (*P* < 0.01), and the expression level of TLR7 mRNA was significantly decreased (*P* < 0.01) in the OVA group. After drug intervention, TLR2 mRNA expression levels were significantly lower (*P* < 0.01) (*P* < 0.01), and the expression level of TLR7 mRNA was significantly increased (*P* < 0.01) in the SJZD group compared to those in the OVA group (Figure 8).

#### 3.4.4. Effect of SJZD on the Structure and Abundance of Intestinal Flora in Asthmatic Mice

**Figure 9:**
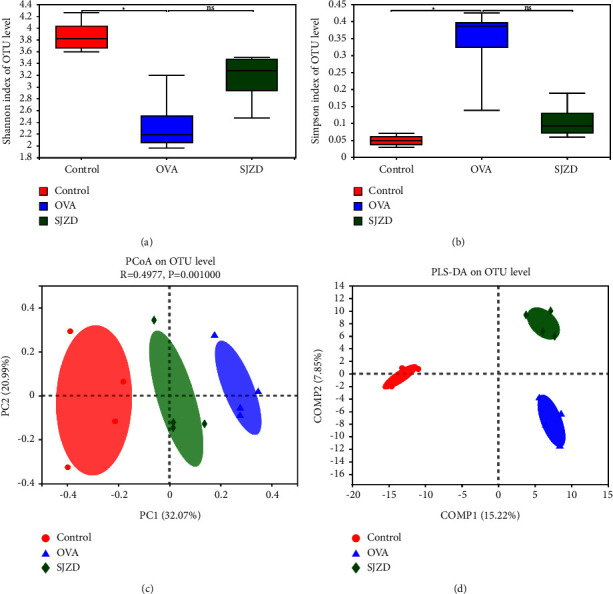
Effect of SJZD on the diversity and structure of microbial flora in asthmatic mice. (a, b) Analysis of alpha diversity (Chao1 and Shannon indices). (c) Principal coordinate (PCoA) analysis. (d) PLS-DA analysis. *n* = four mice/group. ns: nonsignificant, ^*∗*^*P* < 0.05 vs control group.

The Shannon and Simpson indices were used to analyze the species diversity of intestinal flora in each group. The larger the Shannon index, the higher the species diversity, and the larger the Simpson index, the lower the species diversity. The results of the alpha analysis showed that compared with the control group, the Shannon index decreased significantly, and the Simpson index increased in the OVA group. Compared with that of the OVA group, the Shannon index was significantly increased, and the Simpson index was decreased in the SJZD group. The results indicated that the species diversity of the intestinal flora in asthmatic mice was reduced, and the species diversity increased after the traditional Chinese medicine intervention. To further demonstrate the structural differences in species in each group, PCoA and PLS-DA analyses were used to show that bacterial diversity was quite different in the control and OVA groups. The diversity value of the drug intervention group was between that of the control and OVA groups, indicating that SJZD regulated the species structure of the intestinal flora to a certain extent (Figure 9).

#### 3.4.5. Effect of SJZD on Specific Bacteria

**Figure 10:**
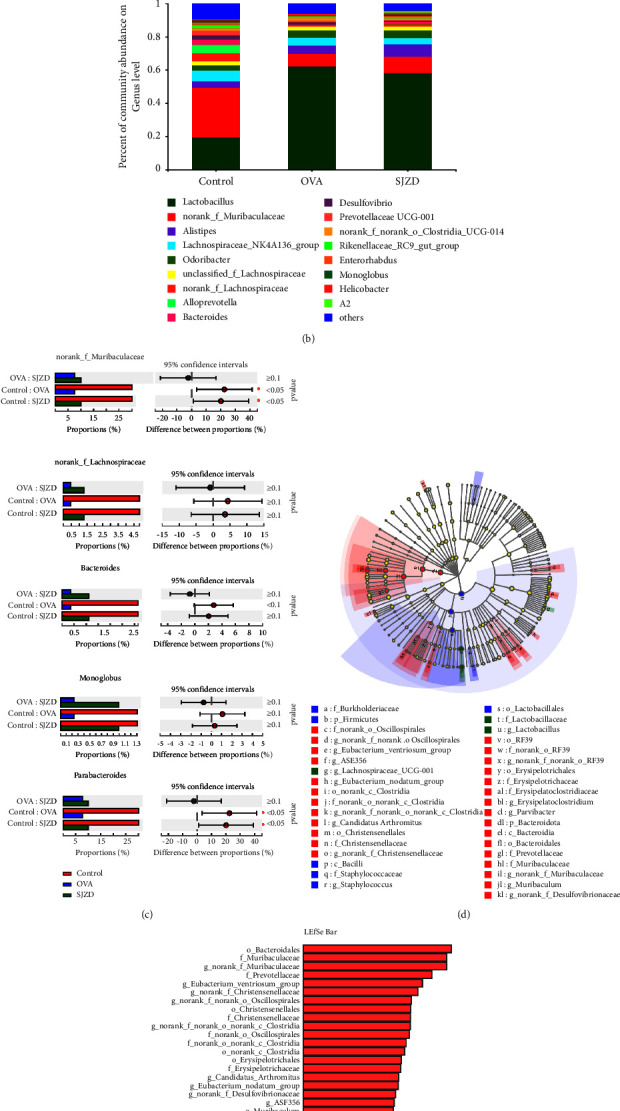
Effect of SJZD on the abundance and diversity of intestinal flora in mice. (a) Variations in intestinal flora at the phylum level. (b) Variations in intestinal flora at the genus level. (c) Bacteria with different relative abundance of intestinal flora at the genus level. (d) Linear discriminant analysis effect size (LEfSe) species difference analysis. (e) LEfSe-based distribution histogram; LDA scores for each group of colony samples.

We further analyzed the specific effect of SJZD on the structure of the intestinal flora of asthmatic mice and found that at the phylum level, *Firmicutes* and *Bacteroidota* were the dominant bacteria in all groups. Compared with the control group, *Firmicutes* showed different degrees of increase, and the abundance of *Bacteroidota* decreased in the OVA group. In the SJZD group, the relative abundance of *Firmicutes* was decreased and that of *Bacteroidota* was increased (Figure 10(a)). In order to observe the specific changes in the structure of the intestinal flora of mice treated with SJZD, further analysis was performed at the genus level. The results showed that the relative abundance of *norank_f__Muribaculacea*, *norank_f__Lachnospiraceae*, *Bacteroides*, *Monoglobus*, and *Parabacteroides* increased after drug intervention (Figures 10(b) and 10(c)). We used LEfSe analysis to generate cladograms to show the relevant specific bacterial genera in each group and applied linear discriminant analysis (LDA) histograms to analyze information of species that were significantly more abundant within one group than in other groups **(**Figures 10(d) and 10(e)).  Figure 10 shows the significant differences in bacteria in each group (LDA >2). *Norank_f__Muribaculacea* was the most enriched in the control group. *Staphylococcus* was the most abundant in the OVA group; *Gemella* and *Lachnospiraceae_UCG-001* were the most enriched in the drug intervention group. The aforementioned results indicated that SJZD could improve the relative abundance of some bacterial genera in the intestinal flora of mice to a certain extent and improve the composition of the intestinal flora in asthmatic mice.

#### 3.4.6. Correlation between the Relative Abundance of Mouse Intestinal Flora and the Expression Level of TLRs

**Figure 11:**
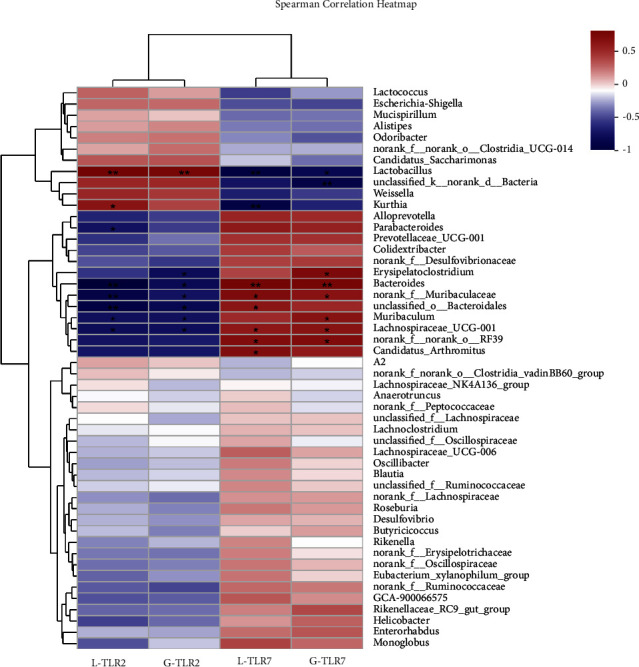
Correlation between the relative abundance of intestinal flora at the genus levels and the expression of TLR2 and TLR7. (^*∗*^*P* < 0.05, ^*∗∗*^*P* < 0.01; blue represents negative correlation; red represents positive correlation; L-TLR: TLR mRNA in lung tissues, G-TLR: TLR mRNA in the small intestine).

TLRs are nonspecific pattern recognition receptors that recognize various pathogenic microorganisms and directly mediate inflammatory responses. To further analyze the relationship between the expression of TLRs and intestinal flora, we performed a heat map analysis of the correlation between the intestinal flora and the expression of TLR2 and TLR7 receptors in each group. *Bacteroides*, *unclassified-o-Bacteroidates*, *norank-f-norank-o-RF39, norank_f__Muribaculacea*, *Lachnospiraceae-UCG-001*, and *Muribaculum, etc.*, were negatively correlated with TLR2 receptor expression, and *Bacteroides*, *unclassified-o-Bacteroidates*, *norank-f norank-o-RF39*, *norank_f__Muribaculacea*, and *Muribaculum, etc.*, were positively correlated with TLR7 receptor expression (Figure 11).

#### 3.4.7. Correlation between the Relative Abundance of Mouse Intestinal Flora and Levels of Characteristic Cytokines

**Figure 12:**
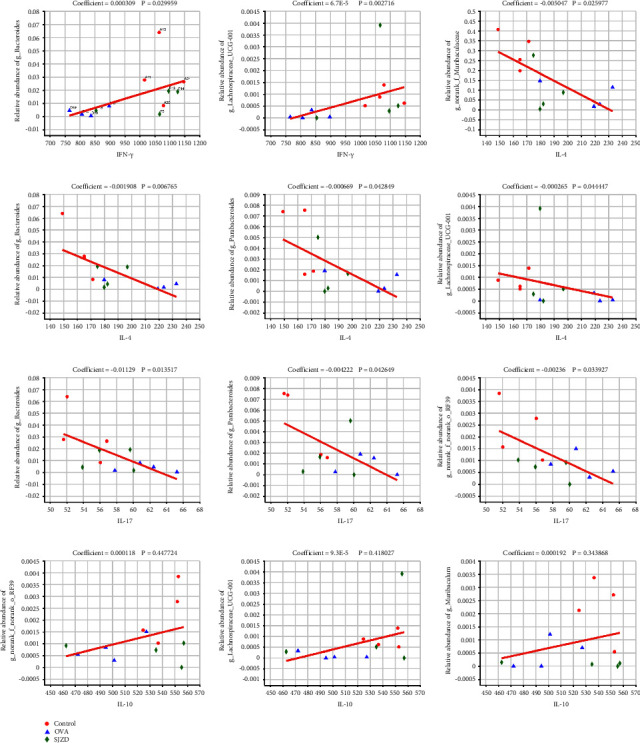
Correlation of differential bacterial genera and cytokine levels in the intestinal flora. The coefficient indicates the magnitude of the correlation coefficient between environmental variables and species (a value higher than 0 indicates a positive correlation, less than 0 indicates a negative correlation, and equal to 0 indicates no correlation). *P* value was used to measure the reliability of the test, and values with *P* < 0.05 indicate that environmental variables were significantly correlated with species.

Disorders of intestinal flora disorders can trigger various systemic chronic inflammatory conditions, and IL-4, IFN-*γ*, IL-17, and IL-10 are potentially correlated with the pathogenesis of asthma. To assess the correlation between intestinal flora and asthma inflammatory factors, we performed a linear correlation analysis between inflammatory factors and genera closely associated with TLR2 and TLR7 receptor expression in the intestinal flora. The results showed that *Bacteroides* and *Lachnospiraceae_UCG-001* were positively correlated with IFN-*γ* levels. *norank_f__Muribaculacea*, *Bacteroides*, *Parabacteroides*, *Lachnospiraceae_UCG-001*, and *norank-f-norank-o-RF39* were negatively correlated with IL-4 and IL-17 levels. *Norank-f-norank-o-RF39*, *Lachnospiraceae_UCG-001*, and *Muribaculacea* were positively correlated with IL-10 levels. These results suggest that alterations in the abundance of intestinal flora are closely associated with the expression of inflammatory factors in asthma (Figure 12).

## 4. Discussion

Episodes of allergic asthma, a common chronic inflammatory disease of the airway, often occur repeatedly and affect the quality of life. Modern medical treatment is often based on bronchodilators, such as *β*2 receptor agonists and anti-inflammatory drugs, such as glucocorticoids. However, repeated use of different medications over a long period of time can lead to a range of side effects, such as hyperglycemia, cardiac arrhythmias, and liver function damage [33]. Chinese traditional medicine practice is characterized by holistic treatment and is widely recognized clinically for its advantages, such as few side effects and a therapeutic approach involving multiple targets. SJZD, a classical formula inherited from Chinese medicine practiced for thousands of years, is often used as a basic formula that shows good therapeutic effects for the treatment of diseases such as asthma, ulcerative colitis, postoperative cancer, and gastritis. Researchers have explored the molecular mechanism of treatment effects in some diseases through network pharmacology analysis. However, the mechanism associated with SJZD for asthma is remains unclear. Structural changes in the intestinal flora are associated with the degree of sensitization and inflammation in asthma. Due to the complex composition of SJZD, we combined network pharmacology and intestinal flora analysis to research the molecular mechanisms underlying the effect of SJZD in treating allergic asthma.

A total of 137 predicted compounds, such as quercetin, kaempferol, beta-sitosterol, medicarpin, and 7-methoxy-2-methyl isoflavone, were derived by constructing an active compound-target network diagram. We concluded that quercetin and kaempferol may be important target compounds of SJZD associated with asthma. Quercetin can reduce airway inflammation and airway hyper-responsiveness by affecting *GATA3* and *T-bet* gene expression and inhibiting the secretion of IL-4, IL-6, and TNF-*α* inflammatory factors [34, 35]. It can also relieve bronchial constriction by blocking L-type voltage-dependent Ca^2+^ and STIM/Orai channels via inhibition of Ca^2+^ release [36]. Kaempferol plays an anti-inflammatory role, and oral kaempferol can inhibit the production of airway secretions and alleviate airway inflammation in asthmatic mice [37]. The aforementioned study also showed that quercetin and kaempferol, both of which are natural flavonoids, have antiallergic effects and can modulate changes in the intestinal flora, enhance intestinal microbial diversity, and increase F/B ratio, especially the relative abundance of the *Bacteroides* [38, 39]. In the experiment, we found that the mice in the drug intervention group were more active behaviorally, and the phenomenon of ear scratching and nose scratching was relatively reduced. The analysis of lung histopathology revealed that the lung inflammation in the allergic asthmatic mice was relieved after drug administration. This supports the predicted results of network pharmacology and demonstrates the success of network pharmacology in exploring the mechanisms of Chinese traditional medicine associated with the treatment of diseases.

The PPI network diagram showed that among the 109 target genes recorded in this study, such as those encoding IL-4, IL-10, MAPK1, VEGFA, CCL2, MYC, and MMP1, genes encoding IL-4 and 1L-10 were highly associated. GO and KEGG analysis as well as target pathway network map analysis indicated that asthma-related BPs closely influenced by SJZD were mainly associated with IL signaling. Furthermore, the signaling pathways included the HIF-1 signaling pathway, PI3K-Akt signaling pathway, TLR signaling pathway, T cell receptor signaling pathway, TNF signaling pathway, and nuclear factor (NF)-*κ*B signaling pathway. Noticeably, the aforementioned signaling pathways are associated with inflammation and can promote the secretion and release of inflammatory mediators, which have a critical effect in the asthma pathogenesis. Abnormally high levels of inflammatory factors have been observed in the pathogenesis of asthma [40]. As important proinflammatory cytokines, IL-4 and IL-17, mediate the aggregation of eosinophils in the respiratory tract [41] and they also activate and stimulate the generation of immunoglobulin (Ig)E, which binds to mast cells and basophil surface receptors, triggering inflammation [42]. IFN-*γ* stimulates the proliferation of Th1 cells and suppresses the secretion of the inflammatory factor IL-4, which significantly increases the inflammatory cells, such as eosinophils and neutrophils, infiltrating around the bronchi and blood vessels [43]. IL-10 can inhibit the secretion of IL-4 and IL-17, reduce the secretory activity of Th2 and Th17 cells, regulate the Th1/Th2 and Th17/Treg balance, effectively limit immune overreaction and inflammatory factors, maintain immune homeostasis, and reduce airway inflammation [33], as well as IFN-*γ*, IL-4, IL-17, and IL-10 as the functional cytokines of Th1, Th2, Th17, and Treg cells, which can reflect the inflammation in asthma [44]. Therefore, IFN-*γ*, IL-17, and asthma-related targets IL-4 and IL-10 were considered inflammatory factors for the evaluation of asthma. Among these predicted potential pathways, the TLR signaling pathway is the key pathway that plays an important role in T cell differentiation, inflammatory response, and lipopolysaccharide-mediated pathways. TLRs are expressed on the surface of airway epithelial cells, T lymphocytes, and other immune cells. These are nonspecific pattern recognition receptors that primarily recognize and combine pathogen-related molecular patterns [45]. The excitation of the TLR signaling pathway triggers multiple intracellular signaling cascades, activates downstream signaling pathways, induces secretion of chemokines and proinflammatory cytokines, and activates T lymphocytes to elicit immune responses [46]. TLRs play a very important role in mediating the immune response against pathogenic microorganisms. Furthermore, they are important proteins involved in stimulating various inflammatory processes and mediating immune imbalance [47].

The intestinal flora colonize the intestine and provide energy to the host by digesting nutrients via proteolytic and glycolytic metabolism. The microbial flora colonizing the intestine are important factors that maintain the overall immune function and immune defense of the host [48]. Living environment and antibiotic abuse can trigger intestinal flora disorders and mediate the development of various diseases [49]. The “hygiene hypothesis” was the first to suggest a relationship between the absence and presence of microbial exposure during early childhood and chronic inflammatory diseases such as asthma [50]. The proportion of *Proteus* in the intestinal flora of patients with asthma is increased and that of *bifidobacteria* and *Bacteroides* is decreased. The relative abundance of *Veillonellaceae* and *Prevotellariceae* in the intestinal flora of patients with severe asthma is higher [51, 52], suggesting that the changes in intestinal flora diversity in patients with asthma are related to the incidence rate of asthma. Alterations in the intestinal flora can lead to exposure or deficiency of certain microorganisms in the body, resulting in abnormal metabolism of the flora activating inflammatory pathways. By interfering with the signaling of pathogen-associated molecular patterns on the microbial surface and Toll-like pattern recognition receptors, inflammatory factors such as IL-4 and IL-17 are secreted to induce an inflammatory response [53, 54]. Therefore, by combining the predicted results of network pharmacology with the analysis of intestinal flora, we hypothesized that the intestinal flora may be involved in the regulation of asthma airway inflammation mediated by SJZD via the intervention of TLR expression to regulate the secretion of inflammatory factors.

We used 16S rDNA amplification sequencing technology to analyze the changes in intestinal flora in asthmatic mice. We found that the intestinal flora of asthmatic mice were structurally disordered. The relative abundance of *Firmicutes* and *Proteobacteria* was increased and that of *Bacteroidota* decreased. In the healthy human gut, *Bacteroidota* and *Firmicutes* account for 98% of the intestinal flora [55]. Furthermore, they play a crucial role in the distal gut [56]. By analyzing stool specimens obtained from patients with asthma and healthy individuals, Lesa Begley et al. found that at the phylum level, the abundance of *Bacteroidota* was low, and the abundance of *Firmicutes* and *Proteobacteria* was increased [57, 58]. After the herbal intervention, we found that SJZD improved the composition of intestinal flora and increased the relative abundance of genera dominated by *norank_f__Muribaculacea*, *Bacteroides*, *norank_f__Lachnospiraceae*, and *Parabacteroides. Norank_f__Muribaculaceae* and *Lachnospiraceae_UCG-001* exerted positive effects on immune regulation, stimulated immune development and defense, and showed anti-inflammatory effects [59–61]. *Bacteroides* have the capacity for immunomodulation, and their encoded bd oxidase renders their colonization of the intestine and positive alterations of the intestinal environment easier [54]. This microbial genus stimulates the differentiation of naïve CD4+ cells to Treg cells, increases IL-10 levels [62], and maintains the balance between Treg cells and proinflammatory Th17 and Th2 cells [63]. A reduction in the relative abundance of *Bacteroides* in the intestine triggers the onset of asthma [64]. *Bacteroides* and *Parabacteroides* are negatively correlated with airway inflammatory response. Increasing the abundance of these genera can improve microbial dysbiosis, regulate the diversity of intestinal microbiota, reduce the levels of inflammatory factors, namely, IL-4, IL-5, and IL-13, and alleviate the development of asthma airway inflammation [65–67]. These findings further suggest that SJZD can increase the relative abundance of beneficial bacteria in asthmatic mice to a certain extent and that the relative abundance levels of bacteria genera are positively correlated with the expression levels of the anti-inflammatory factors IFN-*γ* and IL-10 and negatively correlated with the expression levels of the proinflammatory factors IL-4 and IL-17. Hence, the changes in the structure of intestinal flora may be closely associated with the expression of inflammatory factors.

TLRs act as pattern recognition receptors for pathogenic microorganisms, and their high or low expression activates inflammatory cells to release inflammatory factors. Of these receptors, TLR2 and TLR7 play an important role in the pathogenesis of asthma [68]. Modern medicine suggests that the lungs and intestine are homologous in terms of tissue embryogenesis and maybe similar in terms of susceptibility genes. Furthermore, immune cells and immune molecules in the intestine are recirculated via the blood or lymphocytes. Hence, certain immune-related changes in the intestine may affect the whole body, and subsequently, the lungs [69]. Fu found that the altered relative expression of TLR in the intestinal tissues of asthmatic rats was accompanied by the same trend observed in the relative expression of TLR in the lung tissues, which was believed to be the result of a close association between the intestine and lungs [70]. Owing to this relationship between the lungs and intestine, we examined the expression of TLR2 and TLR7 in the lung and intestinal tissues simultaneously. We observed that the relative expression of TLR2 and TLR7 decreased or increased to different degrees after drug intervention. Based on this result, we further observed the relationship between TLR and intestinal flora. We correlated the genera with TLR expression and found that beneficial genera *Bacteroides, norank_f__Muribaculacea, unclassified-o-Bacteroidates, and Muribaculum*, whose relative abundance increased after drug intervention, showed a negative correlation with TLR2 expression levels and a positive correlation with TLR7 expression levels. TLR2 and TLR7 activate different signaling pathways in response to microbial stimulation and can regulate the secretion of cytokines, such as IFN-*γ*, IL-4, IL-5, and IL-10. Furthermore, they can mediate the differentiation of immune cells, such as Th1, Th2, and Th17 to decrease airway inflammation in patients with asthma [71]. In these patients, TLR2 is expressed at high levels [61], particularly in the large and small airways [72]. TLR2 can recognize many types of bacterial cell wall components. The Gram-positive bacteria *Firmicutes* are the main components of intestinal flora. The increase in the abundance of *Firmicutes* activates TLR2 to promote the secretion of inflammatory factors. This, in turn, triggers an inflammatory response during asthma, recruiting lung eosinophils and triggering airway inflammation [73, 74]. Polymorphisms in the TLR2-encoding gene are associated with the risk of asthma development. Furthermore, *TLR2* gene variants act as determining factors in asthma development [75]. TLR2 receptor activates NF-*κ*B and MAPK signaling pathways, increases infiltration and aggregation of eosinophils and inflammatory factors, such as IL-4 and IL-5, and increases allergic airway inflammation in asthmatic mice. Knockdown of the *TLR2* gene can alleviate airway inflammation in asthmatic mice [76, 77]. TLR7 can induce the expression of IFN-*γ*, decrease the proportion of Th2 cells, enhance Th1/Treg responses to aeroallergens, and reduce proeosinophil aggregation and chemokine and IgE synthesis [78, 79]. The activation of TLR7/8 can enhance IFN*λ* receptor expression and reduce the airway inflammatory response [80]. In this study, we measured the levels of IL-4, IL-17, IFN-*γ*, and IL-10. After drug administration, the levels of proinflammatory factors IL-4 and IL-17 decreased, and the levels of anti-inflammatory factors IFN-*γ* and IL-10 increased, followed by a reduction in airway inflammation and airway mucus secretion. The secretion levels of airway inflammatory factors are abnormally elevated in the pathogenesis of asthma, and these inflammatory factors play a key role in this process [81]. IL-4 stimulates IgE secretion by B lymphocytes, mediates the accumulation of mast cells and eosinophils in the airways, and induces the differentiation of Th2 cells, exacerbating airway hyper-reactivity [82, 83]. IFN-*γ* can inhibit IL-4 secretion and maintain Th1/Th2 balance during asthma by regulating the levels of IL-4 and IFN cytokines to relieve asthma symptoms [84]. IL-10 is a major cytokine secreted by Treg cells and maintains immune tolerance. It shows an anti-inflammatory effect and a powerful immunosuppressive effect during asthma with respect to the expression of the proinflammatory factor IL-17 and the secretory activity of Th2 cells. It can enhance immune tolerance to reduce inflammation [85, 86]. The aforementioned results suggest that while regulating intestinal flora, SJZD influenced the expression of TLR2 and TLR7 to a certain extent along with the secretion levels of proinflammatory and anti-inflammatory factors and decreased airway inflammation in asthmatic mice.

In conclusion, SJZD alleviated the inflammatory state of airways in mice with allergic asthma. We performed network pharmacology analysis to predict the key compounds, key targets, and pathways of SJZD associated with the treatment of asthma and combined the results with those of the analysis of intestinal flora. We studied the main mechanisms of SJZD involved in the treatment of allergic asthma. The mechanisms may be related to the regulation of the intestinal flora. SJZD may increase the relative abundance of beneficial bacteria in the intestine of asthmatic mice and interfere with the expression levels of TLR2 and TLR7 to regulate the secretion of inflammatory factors, thereby alleviating asthmatic airway inflammation. The present study initially explored the mechanism of action of SJZD in the treatment of asthma through network pharmacology and intestinal flora analysis, which provides a foundation for the prevention and treatment of asthma by SJZD and also provides a basis for the multitargeted treatment of asthma by traditional Chinese medicine.

## Figures and Tables

**Table 1:** Primers used in quantitative real-time PCR.

Gene	Forward primer	Reverse primer
TLR2	GCAAACGCTGTTCTGCTCAG	AGGCGTCTCCCTCTATTGTATT
TLR7	ATGTGGACACGGAAGAGACAA	GGTAAGGGTAAGATTGGTGGTG
*β*-actin	GATATCGCTGCGCTGGTCG	GGTAAGGGTAAGATTGGTGGTG

**Table 2:** Top 10 active compounds in Sijunzi decoction (SJZD) listed by degree.

Mol ID	Compounds	OB (%)	DL	Molecules structure
MOL000422	Kaempferol	41.88	0.24	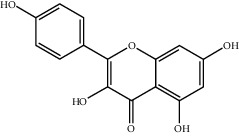

MOL000098	Quercetin	46.43	0.28	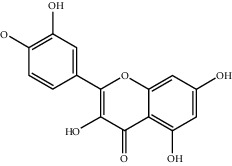

MOL000358	Beta-sitosterol	36.91	0.75	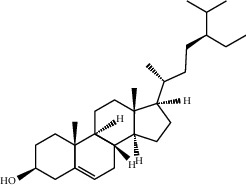

MOL002565	Medicarpin	49.22	0.34	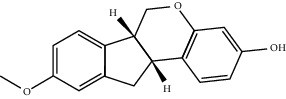

MOL003896	7-Methoxy-2-methyl isoflavone	42.56	0.20	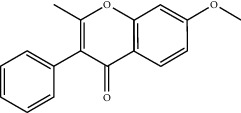

MOL000449	Stigmasterol	43.83	0.76	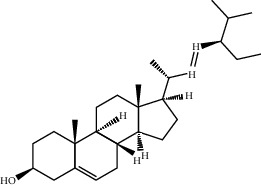

MOL000392	Formononetin	69.67	0.21	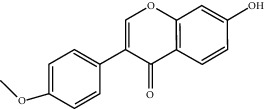

MOL000500	Vestitol	74.66	0.21	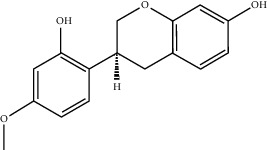

MOL004328	Naringenin	59.29	0.21	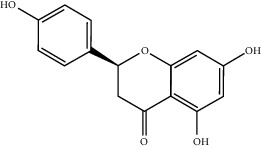

MOL000787	Fumarine	59.26	0.83	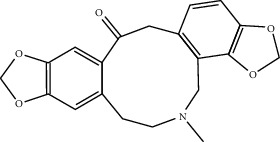 0

## Data Availability

The data used to support this work are available from the corresponding authors upon reasonable request.

## Supplementary Materials

Supplementary Materials Supplementary Table 1: active compounds from databases and literature in Sijunzi decoction. Supplementary Table 2: asthma-related genes in the database. Supplementary Table 3: common genes of asthma and Sijunzi decoction. Supplementary Table 4: GO functional categories. Supplementary Table 5: data of KEGG enrichment analysis.
