# Supplementary material for: Integrated Network Pharmacology and Gut Microbiota Analysis to Explore the Mechanism of Sijunzi Decoction Involved in Alleviating Airway Inflammation in a Mouse Model of Asthma
Source: Evid Based Complement Alternat Med. 2023 Jan 3;2023:1130893. doi: 10.1155/2023/1130893 (PMC9831717; doi:10.1155/2023/1130893)
Supplement: Supplementary Materials — Supplementary Table 1: active compounds from databases and literature in Sijunzi decoction. Supplementary Table 2: asthma-related genes in the database. Supplementary Table 3: common genes of asthma and Sijunzi decoction. Supplementary Table 4: GO functional categories. Supplementary Table 5: data of KEGG enrichment analysis. [file 1130893.f1.zip › Supplementary Material Description.docx]

Manuscript number 1130893: Supplementary Material Description

Supplementary Table 1: active compounds from databases and literature in Sijunzi Decoction

Supplementary Table 2: asthma related genes in the database

Supplementary Table 3: common genes of asthma and Sijunzi decoction

Supplementary Table 4: GO functional categories

Supplementary Table 5: data of KEGG enrichment analysis.
