# Supplementary material for: Integrated Network Pharmacology and Gut Microbiota Analysis to Explore the Mechanism of Sijunzi Decoction Involved in Alleviating Airway Inflammation in a Mouse Model of Asthma
Source: Evid Based Complement Alternat Med. 2023 Jan 3;2023:1130893. doi: 10.1155/2023/1130893 (PMC9831717; doi:10.1155/2023/1130893)
Supplement: Supplementary Materials — Supplementary Table 1: active compounds from databases and literature in Sijunzi decoction. Supplementary Table 2: asthma-related genes in the database. Supplementary Table 3: common genes of asthma and Sijunzi decoction. Supplementary Table 4: GO functional categories. Supplementary Table 5: data of KEGG enrichment analysis. [file 1130893.f1.zip › Supplementary Table 1.docx]

**Supplementary Table 1: active compounds from databases and literature in Sijunzi Decoction**

| **The document contains three data tables** | |
| --- | --- |
| Table A | The compounds from TCMSP and literature in Sijunzi Decotion |
| Table B | The compounds from ETCM in Sijunzi Decotion |
| Table 1 | 137 active compounds from TCMSP and ETCM databases and literature in Sijunzi Decoction |

| **Table A: The compounds from TCMSP and literature in Sijunzi Decotion** | | |
| --- | --- | --- |
| **Mol ID** | **Compound** | **Herb** |
| MOL001212 | Loxanol V | Ren Shen |
| MOL001218 | Pisol | Ren Shen |
| MOL001312 | 9-HEXADECENOIC ACID | Ren Shen |
| MOL001392 | Methyl myristate | Ren Shen |
| MOL001396 | PENTADECYLIC ACID | Ren Shen |
| MOL001641 | METHYL LINOLEATE | Ren Shen |
| MOL001706 | 2,6-dimethyl-3,7-octadiene-2,6-diol | Ren Shen |
| MOL001738 | MLI | Ren Shen |
| MOL001817 | Methyl stearate | Ren Shen |
| MOL001818 | Methyl palmitelaidate | Ren Shen |
| MOL001819 | METHYL PENTADECANOATE | Ren Shen |
| MOL001949 | panaxynol | Ren Shen |
| MOL001965 | Dauricine (8CI) | Ren Shen |
| MOL002121 | (1S,4E,8E,10R)-4,8,11,11-tetramethylbicyclo[8.1.0]undeca-4,8-diene | Ren Shen |
| MOL002136 | neocnidilide | Ren Shen |
| MOL002137 | OCT | Ren Shen |
| MOL002307 | 20-Hexadecanoylingenol | Ren Shen |
| MOL002312 | [(3S,4R,5R)-5-[[(2R,3S,4S,5R,6S)-6-(2-acetyl-5-methoxyphenoxy)-3,4,5-trihydroxyoxan-2-yl]methoxy]-3,4-dihydroxyoxolan-3-yl]methyl 3,4,5-trihydroxybenzoate | Ren Shen |
| MOL002323 | L-Adenosine | Ren Shen |
| MOL002377 | Kaempferol-3-arabofuranoside | Ren Shen |
| MOL002526 | 3691-11-0 | Ren Shen |
| MOL002669 | Campesteryl ferulate | Ren Shen |
| MOL000269 | Elemicin | Ren Shen |
| MOL002879 | Diop | Ren Shen |
| MOL000029 | beta-Humulene | Ren Shen |
| MOL000449 | Stigmasterol | Ren Shen |
| MOL003346 | Psuedohypericin | Ren Shen |
| MOL000358 | beta-sitosterol | Ren Shen |
| MOL000036 | beta-caryophyllene | Ren Shen |
| MOL003648 | Inermin | Ren Shen |
| MOL003845 | Folinic acid | Ren Shen |
| MOL003902 | methyl (Z)-icos-11-enoate | Ren Shen |
| MOL004100 | N-Salicylidene-salicylamine | Ren Shen |
| MOL004174 | epsilon-Cadinene | Ren Shen |
| MOL000422 | kaempferol | Ren Shen |
| MOL004237 | δ-elemene | Ren Shen |
| MOL004275 | (1R,4E,7E,11R)-1,5,9,9-tetramethyl-12-oxabicyclo[9.1.0]dodeca-4,7-diene | Ren Shen |
| MOL004492 | Chrysanthemaxanthin | Ren Shen |
| MOL004498 | 12-O-Nicotinoylisolineolone | Ren Shen |
| MOL004647 | TDA | Ren Shen |
| MOL005155 | ginsenoside Ro_qt | Ren Shen |
| MOL005269 | (+)-Maalioxide | Ren Shen |
| MOL005270 | n-Heptadecanol | Ren Shen |
| MOL005271 | 1-HEXADECYNE | Ren Shen |
| MOL005272 | 13-Tetradecenyl acetate | Ren Shen |
| MOL005273 | 16-Oxoseratenediol | Ren Shen |
| MOL005274 | Neohexane | Ren Shen |
| MOL005275 | 2,3,4-Trimethyldecane | Ren Shen |
| MOL005276 | 2,3,8-Trimethyldecane | Ren Shen |
| MOL005277 | 2,6,10,15-tetramethylheptadecane | Ren Shen |
| MOL005278 | 2-METHYLTRIDECANE | Ren Shen |
| MOL005279 | ginsenoside-Rh1 | Ren Shen |
| MOL005280 | ginsenoside-Rh1_qt | Ren Shen |
| MOL005281 | 20(S)-Ginsenoside-Rh1 | Ren Shen |
| MOL005282 | 20(S)-Ginsenoside-Rh1_qt | Ren Shen |
| MOL005283 | 20(S)-ginsenoside-Rg2 | Ren Shen |
| MOL005284 | (3S,5R,6S,8R,9R,10R,12R,13R,14R,17S)-17-[(2S)-2-hydroxy-6-methylhept-5-en-2-yl]-4,4,8,10,14-pentamethyl-2,3,5,6,7,9,11,12,13,15,16,17-dodecahydro-1H-cyclopenta[a]phenanthrene-3,6,12-triol | Ren Shen |
| MOL005285 | 20(s)-protopanaxadiol | Ren Shen |
| MOL005286 | 20(R)-ginsenoside Rg2 | Ren Shen |
| MOL005287 | 20-(S)-Ginsenoside-Rg3 | Ren Shen |
| MOL005288 | 20-(S)-Ginsenoside-Rg3_qt | Ren Shen |
| MOL005289 | 3,4-Dimethylheptane | Ren Shen |
| MOL005290 | 3,5-Dimethyl-p-anisic acid | Ren Shen |
| MOL005291 | 3-O-beta-D-Glucuronopyranosyl gypsogenin | Ren Shen |
| MOL005292 | 3-O-beta-D-Glucuronopyranosyl gypsogenin_qt | Ren Shen |
| MOL005293 | 3-Ethyl-3-methylheptane | Ren Shen |
| MOL005294 | 3-methylheptane | Ren Shen |
| MOL005295 | 3-methylundecane | Ren Shen |
| MOL005296 | 4-Methyldodecane | Ren Shen |
| MOL005297 | 5-Isobutylnonane | Ren Shen |
| MOL005298 | 5-heptadec-12-enylresorcinol | Ren Shen |
| MOL005299 | 5-methyl-tetradecane | Ren Shen |
| MOL005300 | 6'-Malonylginsenoside Rd1 | Ren Shen |
| MOL005301 | 6'-Malonylginsenoside Rd1_qt1 | Ren Shen |
| MOL005302 | 7-(beta-Xylosyl)cephalomannine | Ren Shen |
| MOL005303 | 7-Tetradecyne | Ren Shen |
| MOL005304 | 7alpha-L-Rhamnosyl-6-methoxylutcolin | Ren Shen |
| MOL005305 | Nepetin | Ren Shen |
| MOL005306 | Acetal | Ren Shen |
| MOL005307 | Adenosine triphosphate | Ren Shen |
| MOL005308 | Aposiopolamine | Ren Shen |
| MOL005309 | Araloside A | Ren Shen |
| MOL005310 | (4aS,6aR,6aS,6bR,8aR,10S,12aR,14bR)-10-hydroxy-2,2,6a,6b,9,9,12a-heptamethyl-1,3,4,5,6,6a,7,8,8a,10,11,12,13,14b-tetradecahydropicene-4a-carboxylic acid | Ren Shen |
| MOL005311 | Argininyl-fructosyl-glucose | Ren Shen |
| MOL005312 | Argininyl-fructosyl-glucose_qt | Ren Shen |
| MOL005313 | 5-[(3aS,6R,6aR)-2-keto-1,3,3a,4,6,6a-hexahydrothieno[3,4-d]imidazol-6-yl]valeric acid | Ren Shen |
| MOL005314 | Celabenzine | Ren Shen |
| MOL005315 | (R)-()-Citronellal | Ren Shen |
| MOL005316 | MAV | Ren Shen |
| MOL005317 | Deoxyharringtonine | Ren Shen |
| MOL005318 | Dianthramine | Ren Shen |
| MOL005319 | Ditertbutyl phthalate | Ren Shen |
| MOL005320 | arachidonate | Ren Shen |
| MOL005321 | Frutinone A | Ren Shen |
| MOL005322 | Gamma-Selinene | Ren Shen |
| MOL005323 | ginsenoside La | Ren Shen |
| MOL005324 | ginsenoside La_qt | Ren Shen |
| MOL005325 | ginsenoside Ro | Ren Shen |
| MOL005326 | Ginsenoside-Ra0 | Ren Shen |
| MOL005327 | Gypnoside V_qt | Ren Shen |
| MOL005328 | Ginsenoside-Ra1 | Ren Shen |
| MOL005329 | Ginsenoside-Ra2 | Ren Shen |
| MOL005330 | Ginsenoside-Ra3 | Ren Shen |
| MOL005331 | ginsenoside Rb1 | Ren Shen |
| MOL005332 | (3R,5R,8R,9R,10R,12R,13R,14R,17S)-17-[(2S)-2-hydroxy-6-methylhept-5-en-2-yl]-4,4,8,10,14-pentamethyl-2,3,5,6,7,9,11,12,13,15,16,17-dodecahydro-1H-cyclopenta[a]phenanthrene-3,12-diol | Ren Shen |
| MOL005333 | ginsenoside-Rb2 | Ren Shen |
| MOL005334 | (3S,5R,8R,9R,10R,12R,13R,14R,17S)-17-[(2S)-2-hydroxy-6-methylhept-5-en-2-yl]-4,4,8,10,14-pentamethyl-2,3,5,6,7,9,11,12,13,15,16,17-dodecahydro-1H-cyclopenta[a]phenanthrene-3,12-diol | Ren Shen |
| MOL005335 | Gypenoside LXIX | Ren Shen |
| MOL005336 | ginsenoside-Rc | Ren Shen |
| MOL005337 | (2S,3R,4S,5S,6R)-2-[(2S)-2-[(3S,5R,8R,9R,10R,12R,13R,14R,17S)-3-[(2R,3R,4S,5S,6R)-4,5-dihydroxy-6-(hydroxymethyl)-3-[(2S,3R,4S,5S,6R)-3,4,5-trihydroxy-6-(hydroxymethyl)oxan-2-yl]oxyoxan-2-yl]oxy-12-hydroxy-4,4,8,10,14-pentamethyl-2,3,5,6,7,9,11,12,13,15,1 | Ren Shen |
| MOL005338 | Ginsenoside Re | Ren Shen |
| MOL011400 | ginsenoside rf | Ren Shen |
| MOL005340 | (3S,5R,6S,8R,9R,10R,12R,13R,14R,17S)-17-[(2R)-2-hydroxy-6-methylhept-5-en-2-yl]-4,4,8,10,14-pentamethyl-2,3,5,6,7,9,11,12,13,15,16,17-dodecahydro-1H-cyclopenta[a]phenanthrene-3,6,12-triol | Ren Shen |
| MOL005341 | Sanchinoside C1 | Ren Shen |
| MOL005342 | Ginsenoside-Rg3 | Ren Shen |
| MOL005343 | Ginsenoside-Rg3_qt | Ren Shen |
| MOL005344 | ginsenoside rh2 | Ren Shen |
| MOL005345 | (2R,3S,4S,5R,6R)-2-(hydroxymethyl)-6-[[(3S,5R,8R,9R,10R,12R,13R,14R,17S)-12-hydroxy-4,4,8,10,14-pentamethyl-17-[(2Z)-6-methylhepta-2,5-dien-2-yl]-2,3,5,6,7,9,11,12,13,15,16,17-dodecahydro-1H-cyclopenta[a]phenanthren-3-yl]oxy]oxane-3,4,5-triol | Ren Shen |
| MOL005346 | Ginsenoside-Rh3_qt | Ren Shen |
| MOL005347 | Ginsenoside-Rh4 | Ren Shen |
| MOL005348 | Ginsenoside-Rh4_qt | Ren Shen |
| MOL005349 | Ginsenoside-Rs1 | Ren Shen |
| MOL005350 | Ginsenoside-Rs2 | Ren Shen |
| MOL005351 | Ginsenoyne A | Ren Shen |
| MOL005352 | Ginsenoyne B | Ren Shen |
| MOL005353 | Ginsenoyne C | Ren Shen |
| MOL005354 | Ginsenoyne D | Ren Shen |
| MOL005355 | Ginsenoyne E | Ren Shen |
| MOL005356 | Girinimbin | Ren Shen |
| MOL005357 | Gomisin B | Ren Shen |
| MOL005358 | L-erythro-isocitric acid | Ren Shen |
| MOL005359 | D-erythro-Isocitric acid | Ren Shen |
| MOL005360 | malkangunin | Ren Shen |
| MOL005361 | Malonylginsenoside Rc | Ren Shen |
| MOL005362 | Malonylginsenoside Rc_qt1 | Ren Shen |
| MOL005363 | Malonylginsenoside Rd | Ren Shen |
| MOL005364 | Malonylginsenoside Rd_qt | Ren Shen |
| MOL005365 | MAL | Ren Shen |
| MOL005366 | Malvic acid | Ren Shen |
| MOL005367 | GUP | Ren Shen |
| MOL005368 | Methyl tricosanoate | Ren Shen |
| MOL005369 | Mycosinol | Ren Shen |
| MOL005370 | NN-Dimethyldecanamide | Ren Shen |
| MOL005371 | Nonacosanediol-6,8 | Ren Shen |
| MOL005372 | notoginsenoside R2 | Ren Shen |
| MOL005373 | notoginsenoside R2_qt | Ren Shen |
| MOL005374 | Notoginsenoside R6 | Ren Shen |
| MOL005375 | Stearyl acetate | Ren Shen |
| MOL005376 | Panaxadiol | Ren Shen |
| MOL007500 | panaxatriol | Ren Shen |
| MOL005378 | Panaxytriol | Ren Shen |
| MOL005379 | Pancratistatin | Ren Shen |
| MOL005380 | Pandamine | Ren Shen |
| MOL005381 | 2-Formylpyrrole | Ren Shen |
| MOL005382 | Ramalic acid | Ren Shen |
| MOL005383 | Methylselenocysteine | Ren Shen |
| MOL005384 | suchilactone | Ren Shen |
| MOL005385 | Suffruticoside A_qt1 | Ren Shen |
| MOL005386 | Vulgarin | Ren Shen |
| MOL006651 | Trifolirhizin | Ren Shen |
| MOL005388 | Undecane, 3,6-dimethyl | Ren Shen |
| MOL005389 | PANGAMIC ACID | Ren Shen |
| MOL005390 | 3-[[(2S)-2,4-dihydroxy-3,3-dimethylbutanoyl]amino]propanoic acid | Ren Shen |
| MOL005391 | (Z,Z)-alpha-farnesene | Ren Shen |
| MOL005392 | alpha-Guttiferin | Ren Shen |
| MOL000908 | beta-elemene | Ren Shen |
| MOL005394 | (Z)-2-methyl-5-[(1S,2R,4R)-2-methyl-3-methylene-2-norbornanyl]pent-2-en-1-ol | Ren Shen |
| MOL000035 | beta-Selinene | Ren Shen |
| MOL005396 | cis-Widdrol alpha-epoxide | Ren Shen |
| MOL005397 | Dammarane | Ren Shen |
| MOL005398 | alexandrin | Ren Shen |
| MOL005399 | alexandrin_qt | Ren Shen |
| MOL005400 | ginsenoside Rg5 | Ren Shen |
| MOL005401 | ginsenoside Rg5_qt | Ren Shen |
| MOL005402 | Methyl margarate | Ren Shen |
| MOL005403 | oleanane | Ren Shen |
| MOL005404 | p-Glucosyloxymandelonitrile | Ren Shen |
| MOL000628 | darutoside | Ren Shen |
| MOL000066 | alloaromadedrene | Ren Shen |
| MOL000676 | DBP | Ren Shen |
| MOL000069 | palmitic acid | Ren Shen |
| MOL000749 | Linoleic | Ren Shen |
| MOL000787 | Fumarine | Ren Shen |
| MOL000864 | MYS | Ren Shen |
| MOL000874 | paeonol | Ren Shen |
| MOL000879 | methyl palmitate | Ren Shen |
| MOL000886 | tetradecane | Ren Shen |
| MOL000935 | Hepanal | Ren Shen |
| MOL000942 | (1R,4S,4aR,8aR)-4-isopropyl-1,6-dimethyl-3,4,4a,7,8,8a-hexahydro-2H-naphthalen-1-ol | Ren Shen |
| MOL000968 | beta-Bisabolene | Ren Shen |
| MOL000018 | (+/-)-Isoborneol | Bai Zhu |
| MOL000019 | D-Camphene | Bai Zhu |
| MOL000020 | 12-senecioyl-2E,8E,10E-atractylentriol | Bai Zhu |
| MOL000021 | 14-acetyl-12-senecioyl-2E,8E,10E-atractylentriol | Bai Zhu |
| MOL000022 | 14-acetyl-12-senecioyl-2E,8Z,10E-atractylentriol | Bai Zhu |
| MOL000023 | Hemo-sol | Bai Zhu |
| MOL000024 | alpha-humulene | Bai Zhu |
| MOL000025 | α-Longipinene | Bai Zhu |
| MOL000026 | stigmast-22E-en-3beta-ol | Bai Zhu |
| MOL000027 | alpha-Curcumene | Bai Zhu |
| MOL000028 | α-Amyrin | Bai Zhu |
| MOL000029 | beta-Humulene | Bai Zhu |
| MOL000030 | (1R)-2-methyl-1-phenylprop-2-en-1-ol | Bai Zhu |
| MOL000031 | (3S)-3-[(1R)-1,5-dimethylhex-4-enyl]-6-methylenecyclohexene | Bai Zhu |
| MOL000032 | beta-Eudesmol | Bai Zhu |
| OL000033 | (3S,8S,9S,10R,13R,14S,17R)-10,13-dimethyl-17-[(2R,5S)-5-propan-2-yloctan-2-yl]-2,3,4,7,8,9,11,12,14,15,16,17-dodecahydro-1H-cyclopenta[a]phenanthren-3-ol | Bai Zhu |
| MOL000034 | 2-[(1R,3S,4S)-3-isopropenyl-4-methyl-4-vinylcyclohexyl]propan-2-ol | Bai Zhu |
| MOL000035 | beta-Selinene | Bai Zhu |
| MOL000036 | beta-caryophyllene | Bai Zhu |
| MOL000037 | γ-elemene | Bai Zhu |
| MOL000038 | Akridin | Bai Zhu |
| MOL000039 | (1S,2R,4R)-Neoiso-dihydrocarveol | Bai Zhu |
| MOL000040 | Scopoletol | Bai Zhu |
| MOL000041 | PHA | Bai Zhu |
| MOL000042 | LPG | Bai Zhu |
| MOL000043 | atractylenolide i | Bai Zhu |
| MOL000044 | atractylenolideII | Bai Zhu |
| MOL000045 | atractylenolide iii | Bai Zhu |
| MOL000046 | atractylone | Bai Zhu |
| MOL000047 | juniper camphor | Bai Zhu |
| MOL000048 | (5E,9Z)-3,6,10-trimethyl-4,7,8,11-tetrahydrocyclodeca[b]furan | Bai Zhu |
| MOL000049 | 3β-acetoxyatractylone | Bai Zhu |
| MOL000050 | GLY | Bai Zhu |
| MOL000051 | Polymannose | Bai Zhu |
| MOL000052 | Gulutamine | Bai Zhu |
| MOL000053 | Methose | Bai Zhu |
| MOL000054 | L- | Bai Zhu |
| MOL000055 | L-Lysin | Bai Zhu |
| MOL000056 | DTY | Bai Zhu |
| MOL000057 | DIBP | Bai Zhu |
| MOL000058 | 2-[(2R,5S,6S)-6,10-dimethylspiro[4.5]dec-9-en-2-yl]propan-2-ol | Bai Zhu |
| MOL000059 | uridine | Bai Zhu |
| MOL000060 | selina-4(14),7(11)-dien-8-one | Bai Zhu |
| MOL000061 | Prolinum | Bai Zhu |
| MOL000062 | biatractylolide | Bai Zhu |
| MOL000063 | ATRACTYLODES MACROCEPHALA | Bai Zhu |
| MOL000064 | D-Serin | Bai Zhu |
| MOL000065 | ASI | Bai Zhu |
| MOL000066 | alloaromadedrene | Bai Zhu |
| MOL000067 | L-Valin | Bai Zhu |
| MOL000068 | L-Ile | Bai Zhu |
| MOL000069 | palmitic acid | Bai Zhu |
| MOL000070 | Ethyl pivaloylacetate | Bai Zhu |
| MOL000071 | Istidina | Bai Zhu |
| MOL000072 | 8β-ethoxy atractylenolide Ⅲ | Bai Zhu |
| MOL000273 | (2R)-2-[(3S,5R,10S,13R,14R,16R,17R)-3,16-dihydroxy-4,4,10,13,14-pentamethyl-2,3,5,6,12,15,16,17-octahydro-1H-cyclopenta[a]phenanthren-17-yl]-6-methylhept-5-enoic acid | Fu Ling |
| MOL000274 | 3β-hydroxylanosta-7,9(11),24-trien-21-oic acid | Fu Ling |
| MOL000275 | trametenolic acid | Fu Ling |
| MOL000276 | 7,9(11)-dehydropachymic acid | Fu Ling |
| MOL000277 | tumulosic acid | Fu Ling |
| MOL000278 | Beta-Glucan | Fu Ling |
| MOL000279 | Cerevisterol | Fu Ling |
| MOL000280 | (2R)-2-[(3S,5R,10S,13R,14R,16R,17R)-3,16-dihydroxy-4,4,10,13,14-pentamethyl-2,3,5,6,12,15,16,17-octahydro-1H-cyclopenta[a]phenanthren-17-yl]-5-isopropyl-hex-5-enoic acid | Fu Ling |
| MOL000281 | Dimethyl L-malate | Fu Ling |
| MOL000282 | ergosta-7,22E-dien-3beta-ol | Fu Ling |
| MOL000283 | Ergosterol peroxide | Fu Ling |
| MOL000284 | L-uridine | Fu Ling |
| MOL000285 | (2R)-2-[(5R,10S,13R,14R,16R,17R)-16-hydroxy-3-keto-4,4,10,13,14-pentamethyl-1,2,5,6,12,15,16,17-octahydrocyclopenta[a]phenanthren-17-yl]-5-isopropyl-hex-5-enoic acid | Fu Ling |
| MOL000286 | β-amyrin acetate | Fu Ling |
| MOL000287 | 3beta-Hydroxy-24-methylene-8-lanostene-21-oic acid | Fu Ling |
| MOL000288 | pachyman | Fu Ling |
| MOL000289 | pachymic acid | Fu Ling |
| MOL000290 | Poricoic acid A | Fu Ling |
| MOL000291 | Poricoic acid B | Fu Ling |
| MOL000292 | poricoic acid C | Fu Ling |
| MOL000293 | poricoic acid D | Fu Ling |
| MOL000294 | poricoic acid DM | Fu Ling |
| MOL000295 | alexandrin | Fu Ling |
| MOL000296 | hederagenin | Fu Ling |
| MOL000297 | Tumulosic acid | Fu Ling |
| MOL000298 | ergosterol | Fu Ling |
| MOL000299 | Trimethyl citrate | Fu Ling |
| MOL000300 | dehydroeburicoic acid | Fu Ling |
| MOL000301 | 2-lauroleic acid | Fu Ling |
| MOL000302 | Undekansaeure | Fu Ling |
| MOL000303 | caprylic acid | Fu Ling |
| MOL000304 | Ethyl glucoside | Fu Ling |
| MOL000305 | lauric acid | Fu Ling |
| MOL000069 | palmitic acid | Fu Ling |
| MOL000105 | protocatechuic acid | Gan Cao |
| MOL001097 | o-xylene | Gan Cao |
| MOL001098 | m-xylene | Gan Cao |
| MOL001099 | p-xylene | Gan Cao |
| MOL000118 | (L)-alpha-Terpineol | Gan Cao |
| MOL000012 | Arachic acid | Gan Cao |
| MOL001484 | Inermine | Gan Cao |
| MOL001543 | Vicenin-2 | Gan Cao |
| MOL001599 | α-cubebol | Gan Cao |
| MOL001696 | Morusin | Gan Cao |
| MOL001737 | ICO | Gan Cao |
| MOL001789 | isoliquiritigenin | Gan Cao |
| MOL001792 | DFV | Gan Cao |
| MOL001850 | Izoforon | Gan Cao |
| MOL000211 | Mairin | Gan Cao |
| MOL002137 | OCT | Gan Cao |
| MOL002166 | ISOHEPTANE | Gan Cao |
| MOL002198 | Heptan | Gan Cao |
| MOL002311 | Glycyrol | Gan Cao |
| MOL000239 | Jaranol | Gan Cao |
| MOL002547 | 21987_FLUKA | Gan Cao |
| MOL002565 | Medicarpin | Gan Cao |
| MOL000263 | oleanolic acid | Gan Cao |
| MOL002678 | EB | Gan Cao |
| MOL002693 | nicotiflorin | Gan Cao |
| MOL002844 | Pinocembrin | Gan Cao |
| MOL002850 | butylated hydroxytoluene | Gan Cao |
| MOL002943 | BuOH | Gan Cao |
| MOL003218 | Neouralenol | Gan Cao |
| MOL000354 | isorhamnetin | Gan Cao |
| MOL000359 | sitosterol | Gan Cao |
| MOL003656 | Lupiwighteone | Gan Cao |
| MOL003662 | 7,4'-Dihydroxyflavone | Gan Cao |
| MOL003686 | Narcissoside | Gan Cao |
| MOL003896 | 7-Methoxy-2-methyl isoflavone | Gan Cao |
| MOL000392 | formononetin | Gan Cao |
| MOL003985 | 2-Caren-10-al | Gan Cao |
| MOL000040 | Scopoletol | Gan Cao |
| MOL000415 | rutin | Gan Cao |
| MOL000417 | Calycosin | Gan Cao |
| MOL000422 | kaempferol | Gan Cao |
| MOL004328 | naringenin | Gan Cao |
| MOL000437 | Hirsutrin | Gan Cao |
| MOL000445 | 8-Prenylwighteone | Gan Cao |
| MOL004589 | Methylheptane | Gan Cao |
| MOL000467 | Castanin | Gan Cao |
| MOL004723 | beta-Terpinene | Gan Cao |
| MOL000475 | anethole | Gan Cao |
| MOL004801 | 2',7-Dihydroxy-4'-methoxyisoflavan-7-O-β-d-glucopyranoside | Gan Cao |
| MOL004802 | (E)-1-butoxyhex-2-ene | Gan Cao |
| MOL004803 | 3-Hydroxyglabrol | Gan Cao |
| MOL004804 | 18beta-glycyrrhetinic acid | Gan Cao |
| MOL004805 | (2S)-2-[4-hydroxy-3-(3-methylbut-2-enyl)phenyl]-8,8-dimethyl-2,3-dihydropyrano[2,3-f]chromen-4-one | Gan Cao |
| MOL004806 | euchrenone | Gan Cao |
| MOL004807 | glucuronic acid | Gan Cao |
| MOL004808 | glyasperin B | Gan Cao |
| MOL004809 | glyasperin E | Gan Cao |
| MOL004810 | glyasperin F | Gan Cao |
| MOL004811 | Glyasperin C | Gan Cao |
| MOL004812 | glyasperins D | Gan Cao |
| MOL004813 | glyasperins Z | Gan Cao |
| MOL004814 | Isotrifoliol | Gan Cao |
| MOL004815 | (E)-1-(2,4-dihydroxyphenyl)-3-(2,2-dimethylchromen-6-yl)prop-2-en-1-one | Gan Cao |
| MOL004816 | (2R)-1-[2,4-dihydroxy-5-(3-methylbut-2-enyl)phenyl]-2-hydroxy-3-[4-hydroxy-3-(3-methylbut-2-enyl)phenyl]propan-1-one | Gan Cao |
| MOL004817 | kanzonols K | Gan Cao |
| MOL004818 | kanzonols L | Gan Cao |
| MOL004819 | kanzonols T | Gan Cao |
| MOL004820 | kanzonols W | Gan Cao |
| MOL004821 | kanzonols X | Gan Cao |
| MOL004822 | (E)-1-(2,4-dihydroxyphenyl)-3-[4-hydroxy-3-(3-methylbut-2-enyl)phenyl]prop-2-en-1-one | Gan Cao |
| MOL004823 | licoagropin | Gan Cao |
| MOL004824 | (2S)-6-(2,4-dihydroxyphenyl)-2-(2-hydroxypropan-2-yl)-4-methoxy-2,3-dihydrofuro[3,2-g]chromen-7-one | Gan Cao |
| MOL004825 | glyinflanin A | Gan Cao |
| MOL005812 | naringin | Gan Cao |
| MOL004827 | Semilicoisoflavone B | Gan Cao |
| MOL004828 | Glepidotin A | Gan Cao |
| MOL004829 | Glepidotin B | Gan Cao |
| MOL004830 | Octadiene | Gan Cao |
| MOL004831 | (E)-1-[2,4-dihydroxy-3-(3-methylbut-2-enyl)phenyl]-3-[4-hydroxy-3-(3-methylbut-2-enyl)phenyl]prop-2-en-1-one | Gan Cao |
| MOL004832 | WLN: 4OVR | Gan Cao |
| MOL004833 | Phaseolinisoflavan | Gan Cao |
| MOL004834 | 3-(2-hydroxy-4-methoxyphenyl)-2H-chromen-7-ol | Gan Cao |
| MOL004835 | Glypallichalcone | Gan Cao |
| MOL004836 | echinatin | Gan Cao |
| MOL004837 | Karenzu DK2 | Gan Cao |
| MOL004838 | 8-(6-hydroxy-2-benzofuranyl)-2,2-dimethyl-5-chromenol | Gan Cao |
| MOL004839 | (1S,2S)-1,2-dimethylcyclopentane | Gan Cao |
| MOL004840 | Liconeolignan | Gan Cao |
| MOL004841 | Licochalcone B | Gan Cao |
| MOL004842 | licochalcone C | Gan Cao |
| MOL004843 | licochalconeD | Gan Cao |
| MOL004844 | glabrol | Gan Cao |
| MOL004845 | apioglycyrrhizin | Gan Cao |
| MOL004846 | apioglycyrrhizin_qt | Gan Cao |
| MOL004847 | 2,2-DIMETHYLPENTANE | Gan Cao |
| MOL004848 | licochalcone G | Gan Cao |
| MOL004849 | 3-(2,4-dihydroxyphenyl)-8-(1,1-dimethylprop-2-enyl)-7-hydroxy-5-methoxy-coumarin | Gan Cao |
| MOL004850 | liquoric acid | Gan Cao |
| MOL004851 | Licoflavone | Gan Cao |
| MOL004852 | 7-hydroxy-2-[4-hydroxy-3-(3-methylbut-2-enyl)phenyl]-6-(3-methylbut-2-enyl)chromone | Gan Cao |
| MOL004853 | Licoflavonol | Gan Cao |
| MOL004385 | Yinyanghuo D | Gan Cao |
| MOL004855 | Licoricone | Gan Cao |
| MOL004856 | Gancaonin A | Gan Cao |
| MOL004857 | Gancaonin B | Gan Cao |
| MOL004858 | Gancaonin C | Gan Cao |
| MOL004859 | 2,3-dimethylhexane | Gan Cao |
| MOL000486 | Prunetin | Gan Cao |
| MOL004860 | licorice glycoside E | Gan Cao |
| MOL004861 | Gancaonin D | Gan Cao |
| MOL004862 | (2R)-2-[3,4-dihydroxy-5-(3-methylbut-2-enyl)phenyl]-5,7-dihydroxy-8-(3-methylbut-2-enyl)chroman-4-one | Gan Cao |
| MOL004863 | 3-(3,4-dihydroxyphenyl)-5,7-dihydroxy-8-(3-methylbut-2-enyl)chromone | Gan Cao |
| MOL004864 | 5,7-dihydroxy-3-(4-methoxyphenyl)-8-(3-methylbut-2-enyl)chromone | Gan Cao |
| MOL004865 | 5,7-dihydroxy-3-(2-hydroxy-4-methoxy-phenyl)-6-(3-methylbut-2-enyl)chromone | Gan Cao |
| MOL004866 | 2-(3,4-dihydroxyphenyl)-5,7-dihydroxy-6-(3-methylbut-2-enyl)chromone | Gan Cao |
| MOL004867 | Gancaonin P | Gan Cao |
| MOL004868 | Gancaonin Q | Gan Cao |
| MOL004869 | Gancaonin R | Gan Cao |
| MOL004870 | Gancaonin S | Gan Cao |
| MOL004871 | (3S)-2,3-dimethylpentane | Gan Cao |
| MOL004872 | gancaonin T | Gan Cao |
| MOL004873 | Gancaonin U | Gan Cao |
| MOL004874 | Gancaonin V | Gan Cao |
| MOL004875 | 3-[4,6-dihydroxy-2-methoxy-3-(3-methylbut-2-enyl)phenyl]-7-hydroxy-chromone | Gan Cao |
| MOL004876 | Glycyram | Gan Cao |
| MOL004877 | Licoricidin | Gan Cao |
| MOL004878 | Glycycoumarin | Gan Cao |
| MOL004879 | Glycyrin | Gan Cao |
| MOL004880 | 5,6,7,8-Tetrahydro-2,4-dimethylquinoline | Gan Cao |
| MOL004881 | (E)-1-[2,4-dihydroxy-3-(3-methylbut-2-enyl)phenyl]-3-(2,4-dihydroxyphenyl)prop-2-en-1-one | Gan Cao |
| MOL004882 | Licocoumarone | Gan Cao |
| MOL004883 | Licoisoflavone | Gan Cao |
| MOL004884 | Licoisoflavone B | Gan Cao |
| MOL004885 | licoisoflavanone | Gan Cao |
| MOL004886 | licorice-saponin C2 | Gan Cao |
| MOL004887 | licorice-saponin C2_qt | Gan Cao |
| MOL004888 | licorice-saponin F3 | Gan Cao |
| MOL004889 | licorice-saponin F3_qt | Gan Cao |
| MOL004890 | (4S)-2,4-dimethylhexane | Gan Cao |
| MOL004891 | shinpterocarpin | Gan Cao |
| MOL004892 | licorice-saponin G2 | Gan Cao |
| MOL004893 | licorice-saponin G2_qt | Gan Cao |
| MOL004894 | licorice-saponin H2 | Gan Cao |
| MOL004895 | licorice-saponin H2_qt | Gan Cao |
| MOL004896 | licorice-saponin J2 | Gan Cao |
| MOL004897 | licorice-saponin J2_qt | Gan Cao |
| MOL004898 | (E)-3-[3,4-dihydroxy-5-(3-methylbut-2-enyl)phenyl]-1-(2,4-dihydroxyphenyl)prop-2-en-1-one | Gan Cao |
| MOL004899 | licorice-saponin B2 | Gan Cao |
| MOL004900 | licorice-saponin K2 | Gan Cao |
| MOL004901 | licorice-saponin K2_qt | Gan Cao |
| MOL004902 | glycyrrhetol | Gan Cao |
| MOL004903 | liquiritin | Gan Cao |
| MOL004904 | licopyranocoumarin | Gan Cao |
| MOL004905 | 3,22-Dihydroxy-11-oxo-delta(12)-oleanene-27-alpha-methoxycarbonyl-29-oic acid | Gan Cao |
| MOL004906 | Hispaglabridin B | Gan Cao |
| MOL004907 | Glyzaglabrin | Gan Cao |
| MOL004908 | Glabridin | Gan Cao |
| MOL004909 | glabrolide | Gan Cao |
| MOL004910 | Glabranin | Gan Cao |
| MOL004911 | Glabrene | Gan Cao |
| MOL004912 | Glabrone | Gan Cao |
| MOL004913 | 1,3-dihydroxy-9-methoxy-6-benzofurano[3,2-c]chromenone | Gan Cao |
| MOL004914 | 1,3-dihydroxy-8,9-dimethoxy-6-benzofurano[3,2-c]chromenone | Gan Cao |
| MOL004915 | Eurycarpin A | Gan Cao |
| MOL004916 | 2-methyl-5-propyl -nonane | Gan Cao |
| MOL004917 | glycyroside | Gan Cao |
| MOL004918 | HEX | Gan Cao |
| MOL004919 | Sextone B | Gan Cao |
| MOL004920 | Methylcyclopentane | Gan Cao |
| MOL004921 | Docosyl caffeate | Gan Cao |
| MOL004922 | 2-methyl-6-ethyl decane | Gan Cao |
| MOL000391 | Ononin | Gan Cao |
| MOL004924 | (-)-Medicocarpin | Gan Cao |
| MOL004925 | vitexin | Gan Cao |
| MOL004926 | 4H-1-Benzopyran-4-one, 2-(4-(beta-D-glucopyranosyloxy)phenyl)-2,3-dihydro-5,7-dihydroxy-, (2S)- | Gan Cao |
| MOL004927 | Hispaglabridin A | Gan Cao |
| MOL004928 | violanthin | Gan Cao |
| MOL004929 | Pentadecanol | Gan Cao |
| MOL004930 | Uralenol | Gan Cao |
| MOL004931 | Uralenol-3-methylether | Gan Cao |
| MOL004932 | glycyrrhizin | Gan Cao |
| MOL004933 | uralsaponin B | Gan Cao |
| MOL004934 | Isohexane | Gan Cao |
| MOL004935 | Sigmoidin-B | Gan Cao |
| MOL004936 | Uralene | Gan Cao |
| MOL004937 | uralenneoside | Gan Cao |
| MOL004938 | schaftoside | Gan Cao |
| MOL004939 | Nortangeretin | Gan Cao |
| MOL004940 | neoliquiritin | Gan Cao |
| MOL004941 | (2R)-7-hydroxy-2-(4-hydroxyphenyl)chroman-4-one | Gan Cao |
| MOL004942 | (E)-dodec-2-ene | Gan Cao |
| MOL004943 | neoisoliquiritin | Gan Cao |
| MOL004944 | Cyclobutanol, 1-ethyl- | Gan Cao |
| MOL004945 | (2S)-7-hydroxy-2-(4-hydroxyphenyl)-8-(3-methylbut-2-enyl)chroman-4-one | Gan Cao |
| MOL004946 | 2-Tetradecanone | Gan Cao |
| MOL004947 | Isoviolanthin | Gan Cao |
| MOL004948 | Isoglycyrol | Gan Cao |
| MOL004949 | Isolicoflavonol | Gan Cao |
| MOL004950 | isoglycycoumarin | Gan Cao |
| MOL004951 | Isoliquiritin | Gan Cao |
| MOL004952 | licuraside | Gan Cao |
| MOL004953 | Liquiritin apioside | Gan Cao |
| MOL004954 | isograbrol | Gan Cao |
| MOL004955 | isoglabrolide | Gan Cao |
| MOL004956 | Isoononin | Gan Cao |
| MOL004957 | HMO | Gan Cao |
| MOL004958 | Isoschaftoside | Gan Cao |
| MOL004959 | 1-Methoxyphaseollidin | Gan Cao |
| MOL004960 | 22β-acetylglabric acid | Gan Cao |
| MOL004961 | Quercetin der. | Gan Cao |
| MOL004962 | 24-Hydroxy-11-deoxyglycyrrhetic acid | Gan Cao |
| MOL004963 | 24-Hydroxyglycyrrhetic acid | Gan Cao |
| MOL004964 | (Z)-1-(2,4-dihydroxyphenyl)-3-phenylprop-2-en-1-one | Gan Cao |
| MOL004965 | 3'(γ,γ-dimethylallyl)-kievitone | Gan Cao |
| MOL004966 | 3'-Hydroxy-4'-O-Methylglabridin | Gan Cao |
| MOL004967 | 3,3-Dimethylpentane | Gan Cao |
| MOL004968 | 3,4,3',4'-Tetrahydroxy-2-methoxychalcone | Gan Cao |
| MOL004969 | 2-Ethyl-p-xylene | Gan Cao |
| MOL000497 | licochalcone a | Gan Cao |
| MOL004970 | 3-methylheptane | Gan Cao |
| MOL004971 | 3-methylhexane | Gan Cao |
| MOL004972 | 3-Methylpentane | Gan Cao |
| MOL004973 | 3-Ethylpentane | Gan Cao |
| MOL004974 | 3'-Methoxyglabridin | Gan Cao |
| MOL004975 | 3β-formylglabrolide | Gan Cao |
| MOL004976 | Daidzein dimethyl ether | Gan Cao |
| MOL004977 | 1-Methoxyficifolinol | Gan Cao |
| MOL004978 | 2-[(3R)-8,8-dimethyl-3,4-dihydro-2H-pyrano[6,5-f]chromen-3-yl]-5-methoxyphenol | Gan Cao |
| MOL004979 | 4,2',4',alpha-Tetrahydroxydihydrochalcone | Gan Cao |
| MOL004980 | Inflacoumarin A | Gan Cao |
| MOL004981 | 1-(5-hydroxy-2,2-dimethylchromen-6-yl)-3-(4-hydroxyphenyl)prop-2-en-1-one | Gan Cao |
| MOL004982 | 2,6,10-trimethyl-dodecane | Gan Cao |
| MOL004983 | 5,6,7,8-Tetrahydro-4-methylquinoline | Gan Cao |
| MOL005015 | Licoriisoflavan A | Gan Cao |
| MOL004985 | icos-5-enoic acid | Gan Cao |
| MOL004986 | 6″-O-acetylliquiritin | Gan Cao |
| MOL004987 | 11-deoxyglycyrrhetic acid | Gan Cao |
| MOL004988 | Kanzonol F | Gan Cao |
| MOL004989 | 6-prenylated eriodictyol | Gan Cao |
| MOL004990 | 7,2',4'-trihydroxy－5-methoxy-3－arylcoumarin | Gan Cao |
| MOL004991 | 7-Acetoxy-2-methylisoflavone | Gan Cao |
| MOL004992 | 7-hydroxy-2-methyl-3-phenyl-chromone | Gan Cao |
| MOL004993 | 8-prenylated eriodictyol | Gan Cao |
| MOL004994 | 12-methyltetradecanoate | Gan Cao |
| MOL004995 | Kanzonol H | Gan Cao |
| MOL004996 | gadelaidic acid | Gan Cao |
| MOL004997 | Araboglycyrrhizin | Gan Cao |
| MOL004998 | Araboglycyrrhizin_qt | Gan Cao |
| MOL004999 | Artonin E | Gan Cao |
| MOL000500 | Vestitol | Gan Cao |
| MOL005000 | Gancaonin G | Gan Cao |
| MOL005001 | Gancaonin H | Gan Cao |
| MOL005002 | beta-Glycyrrhetinic acid | Gan Cao |
| MOL005003 | Licoagrocarpin | Gan Cao |
| MOL005004 | Gancaonin I | Gan Cao |
| MOL005005 | Glyasperin A | Gan Cao |
| MOL005006 | Glyasperins K | Gan Cao |
| MOL005007 | Glyasperins M | Gan Cao |
| MOL005008 | Glycyrrhiza flavonol A | Gan Cao |
| MOL005009 | Corylifolinin | Gan Cao |
| MOL005010 | Kanzonol E | Gan Cao |
| MOL005011 | Kanzonol Z | Gan Cao |
| MOL005012 | Licoagroisoflavone | Gan Cao |
| MOL005013 | 18α-hydroxyglycyrrhetic acid | Gan Cao |
| MOL005014 | Licorice glycoside A | Gan Cao |
| MOL005016 | Odoratin | Gan Cao |
| MOL005017 | Phaseol | Gan Cao |
| MOL005018 | Xambioona | Gan Cao |
| MOL005019 | (2R)-7-hydroxy-2-[4-hydroxy-3-(3-methylbut-2-enyl)phenyl]chroman-4-one | Gan Cao |
| MOL005020 | dehydroglyasperins C | Gan Cao |
| MOL005021 | Mipax | Gan Cao |
| MOL000511 | ursolic acid | Gan Cao |
| MOL000561 | Astragalin | Gan Cao |
| MOL000057 | DIBP | Gan Cao |
| MOL000668 | PENTYLFURAN | Gan Cao |
| MOL000671 | ()-Menthol | Gan Cao |
| MOL000676 | DBP | Gan Cao |
| MOL000703 | 2-heptanone | Gan Cao |
| MOL000705 | WLN: VH6 | Gan Cao |
| MOL000098 | quercetin | Gan Cao |
| MOL004876 | glycyrrhizic acid | Gan Cao |
|  |  |  |
|  |  |  |

| **Table B: The compounds from ETCM in Sijunzi Decotion** | |
| --- | --- |
| **Compound** | **Herb** |
| Sitosterol | Ren Shen |
| Cis-9,Cis-12-Linoleic Acid,Inositol,Linoleic,Linoleic Acid | Ren Shen |
| Stigmasterol | Ren Shen |
| Kaempferol | Ren Shen |
| Trifolin | Ren Shen |
| Cedrol,Eudesmol,Î‘-Cedrol | Ren Shen |
| 3,4-Dihydroxybenzaldehyde,Hydroxybenzoic Acid,M-Hydroxybenzoic Acid,P-Hydroxybenzoic Acid,Salicylic Acid | Ren Shen |
| Cetylic Acid,Hexadecanoic Acid,Palmitic Acid | Ren Shen |
| Sucrose | Ren Shen |
| Succinic Acid | Ren Shen |
| Alexandrin,Daucosterol,Caproic Acid,Eleutheroside A,Sitogluside,Strumaroside,Î’-Sitosterol-Î’-D-Glucoside | Ren Shen |
| Riboflavine | Ren Shen |
| Xylose | Ren Shen |
| Citric Acid | Ren Shen |
| Campesterol,M-Cresol | Ren Shen |
| Adenosine,Adenine Nucleoside | Ren Shen |
| 1-Heptadecanol | Ren Shen |
| Malic Acid | Ren Shen |
| Choline | Ren Shen |
| Raffinose | Ren Shen |
| Niacin,Nicotinic Acid | Ren Shen |
| 3-Hydroxycoumarin,Folic Acid | Ren Shen |
| Tartaric Acid | Ren Shen |
| Vitamin B1 | Ren Shen |
| Vitamin B12 | Ren Shen |
| Fructose | Ren Shen |
| Mannose | Ren Shen |
| Rhamnose | Ren Shen |
| Octanal | Ren Shen |
| 2,5-Dimethyl-7-Hydroxy Chromone | Ren Shen |
| Glucuronic Acid | Ren Shen |
| Mannose-B | Ren Shen |
| Adenosine Triphosphate | Ren Shen |
| Araloside A | Ren Shen |
| Biotin | Ren Shen |
| Chikusetsusaponin Iii | Ren Shen |
| Chikusetsusaponin Iv | Ren Shen |
| Deoxygomisin A | Ren Shen |
| Dianthoside | Ren Shen |
| 2,6-Ditertbutyl-4-Methyl Phenol | Ren Shen |
| Ginsenol | Ren Shen |
| Ginsenoside F1 | Ren Shen |
| Ginsenoside F4 | Ren Shen |
| Ginsenoside I | Ren Shen |
| Ginsenoside Ia | Ren Shen |
| Ginsenoside Ib | Ren Shen |
| Ginsenoside Ic | Ren Shen |
| Ginsenoside Ii | Ren Shen |
| Ginsenoside Iii | Ren Shen |
| Ginsenoside La | Ren Shen |
| Ginsenoside Ra0 | Ren Shen |
| Ginsenoside Ra1 | Ren Shen |
| Ginsenoside Ra2 | Ren Shen |
| Ginsenoside Ra3 | Ren Shen |
| Ginsenoside Rb1 | Ren Shen |
| Ginsenoside Rb2 | Ren Shen |
| Ginsenoside Rb3 | Ren Shen |
| Ginsenoside Rc | Ren Shen |
| Ginsenoside Rd | Ren Shen |
| Ginsenoside Re | Ren Shen |
| Ginsenoside Rf | Ren Shen |
| Ginsenoside Rg1 | Ren Shen |
| Ginsenoside Rg2 | Ren Shen |
| 20(S)-Ginsenoside Rg3 | Ren Shen |
| 20(R)-Ginsenoside Rh1 | Ren Shen |
| Ginsenoside-Rh1 | Ren Shen |
| 20(R)-Ginsenoside-Rh2 | Ren Shen |
| Ginsenoside Rh2 | Ren Shen |
| Ginsenoside Rh3 | Ren Shen |
| Ginsenoside Rh4 | Ren Shen |
| Ginsenoside R0 | Ren Shen |
| Ginsenoside Rs1 | Ren Shen |
| Ginsenoside Rs2 | Ren Shen |
| 20-Glucosylginsenoside Rf | Ren Shen |
| Isocitric Acid | Ren Shen |
| Isocitric Acid B | Ren Shen |
| Isocitric Acid C | Ren Shen |
| Isocitric Acid D | Ren Shen |
| Malonylginsenoside Rb2 | Ren Shen |
| Malonylginsenoside Rc | Ren Shen |
| 6'-Malonylginsenoside Rd1 | Ren Shen |
| Maltose | Ren Shen |
| Neointermedeol | Ren Shen |
| Notoginsenoside R1 | Ren Shen |
| Notoginsenoside R4 | Ren Shen |
| Palmitin | Ren Shen |
| Panacon | Ren Shen |
| Panasinsanol A | Ren Shen |
| Panasinsanol B | Ren Shen |
| Panaxacol | Ren Shen |
| Panaxadiol | Ren Shen |
| Panaxatriol | Ren Shen |
| Panaxytriol | Ren Shen |
| Protopanaxadiol | Ren Shen |
| Protopanaxatriol | Ren Shen |
| Pseudoginsenoside F11 | Ren Shen |
| Pyruvic Acid | Ren Shen |
| Quinquenoside R1 | Ren Shen |
| Î’-Sitosterol-3-(6-Linoleoyl)Glucopyranoside | Ren Shen |
| Î’-Sitosterol-3-(6-Palmitoleoyl)Glucopyranoside | Ren Shen |
| Î’-Sitosterol-3-(6-Stearoyl)Glucopyranoside | Ren Shen |
| Stigmasterol-3-(6-Linoleoyl)Glucopyranoside | Ren Shen |
| Stigmasterol-3-(6-Oleoyl)Glucopyranoside | Ren Shen |
| Stigmasterol-3-(6-Stearoyl)Glucopyranoside | Ren Shen |
| Stigmast-3-O-Î’-D-Glucopyanosyl-6-Hexadecanoate | Ren Shen |
| Alpha-Humulene,Humulene,Î‘-Humulene | Bai Zhu |
| Î‘-Curcumene,Î‘-Curcumene(R-) | Bai Zhu |
| Pavilion,Scopoletin,Scopoletol,Trigonelline | Bai Zhu |
| Cedrol,Eudesmol,Î‘-Cedrol | Bai Zhu |
| Fructose | Bai Zhu |
| Atractylenolide III | Bai Zhu |
| Agarospirol,Hinesol | Bai Zhu |
| Î’-Humulene | Bai Zhu |
| Î’-Elemol | Bai Zhu |
| 6-Methoxy-7-Hydroxycoumarin | Bai Zhu |
| Atractylenolide I | Bai Zhu |
| Atractylentriol | Bai Zhu |
| Atractylone | Bai Zhu |
| 8Î’-Ethoxy Atractylenolide Iii | Bai Zhu |
| (+)-Eudesma-4(15),7(11)-Dien-8-One | Bai Zhu |
| Juniper Camphor | Bai Zhu |
| 12-(Î‘-Methyl Butyryl)-14-Acetyl-2E,8E,10E-Atractylentriol | Bai Zhu |
| 12-(Î‘-Methyl Butyryl)-14-Acetyl-2E,8Z,10E-Atractylentriol | Bai Zhu |
| 14(Î‘-Methyl Butyryl)-2E,8E,10E-Atractylentriol | Bai Zhu |
| 14(Î‘-Methyl Butyryl)-2E,8Z,10E-Atractylentriol | Bai Zhu |
| Cetylic Acid,Hexadecanoic Acid,Palmitic Acid | Fu Ling |
| Lauric Acid | Fu Ling |
| Caprylic Acid | Fu Ling |
| Ergosterol | Fu Ling |
| Choline | Fu Ling |
| Dodecenoic Acid | Fu Ling |
| Adenine | Fu Ling |
| Î’-Amyrin Acetate | Fu Ling |
| O-Acetylpachymic Acid-25-Ol | Fu Ling |
| Dehydroabietic Acid Methyl Ester | Fu Ling |
| Dehydroeburicoic Acid | Fu Ling |
| Dehydrotumulosic Acid | Fu Ling |
| Eburicoic Acid | Fu Ling |
| 3-Epidehydrotumulosic Acid | Fu Ling |
| 3Î’-Hydroxy-16Î‘-Acetoxy-Lanosta-7,9(11),24-Trien-21-Oic Acid | Fu Ling |
| 3Î’-P-Hydroxybenzoyldehydrotumulosic Acid | Fu Ling |
| 25-Hydroxy-3-Epidehydrotumulosic Acid | Fu Ling |
| 3Î’-Hydroxylanosta-7,9(11),24-Trien-21-Oic Acid | Fu Ling |
| Pachymic Acid | Fu Ling |
| Pachymic Acid Methyl Ester | Fu Ling |
| Pinicolic Acid A | Fu Ling |
| Polyporenic Acid C | Fu Ling |
| Poricoic Acid A | Fu Ling |
| Poricoic Acid B | Fu Ling |
| Poricoic Acid C | Fu Ling |
| Poricoic Acid D | Fu Ling |
| Poricoic Acid Dm | Fu Ling |
| Poricoic Acid G | Fu Ling |
| Poricoic Acid H | Fu Ling |
| Trametenolic Acid | Fu Ling |
| Tumulosic Acid | Fu Ling |
| Tumulosic Acid Methyl Ester | Fu Ling |
| Undecanoic Acid | Fu Ling |
| Sitosterol,Î’-Sitosterol | Gan Cao |
| Rutin,Rutoside,Vitamin P | Gan Cao |
| Isoquercitrin,Isoquercetrin,Kuwanon H | Gan Cao |
| Nicotiflorin | Gan Cao |
| Corylifolinin,Isobavachalcone | Gan Cao |
| (S)-5,7-Dihydroxy-2-Phenylchroman-4-One,Pinocembrin | Gan Cao |
| Lupiwighteone | Gan Cao |
| 3,3'-Dimethylquercetin | Gan Cao |
| 3-O-Acetyl-Glycyrrhetinic Acid | Gan Cao |
| 6,8-Bis(C-Î’-Glucosyl)-Apigenin,Vicenin-2 | Gan Cao |
| Dibutyl Uralsaponin A Ester | Gan Cao |
| (E)-1-[2,4-Dihydroxy-3-(3-Methyl-2-Butenyl)Phenyl]-3-(2,2-Dimethyl-8-Hydroxy-2H-Benzopyran-6-Yl)-2-Propen-1-One | Gan Cao |
| (E)-1-[2,4-Dihydroxy-3-(3-Methyl-2-Butenyl)Phenyl]-3-(4-Hydroxy-3-[3-Methyl-2-Butenyl)Phenyl]-2-Propen-1-One | Gan Cao |
| 3'-(Î“,Î“-Dimethylallyl)-Kievitone | Gan Cao |
| Ethyl-N-Buthy-Uralsaponin A Esters | Gan Cao |
| Formononetin | Gan Cao |
| Formononetin-7-Glucoside | Gan Cao |
| Gancaonin A | Gan Cao |
| Gancaonin B | Gan Cao |
| Gancaonin C | Gan Cao |
| Gancaonin D | Gan Cao |
| Gancaonin E | Gan Cao |
| Gancaonin F | Gan Cao |
| Gancaonin I | Gan Cao |
| Gancaonin P-3'-Methylether | Gan Cao |
| Gancaonin X | Gan Cao |
| Glabrolide | Gan Cao |
| Glisoflavanone | Gan Cao |
| 3-O-[Î’-D-Glucuronopyranosyl-(1â†’2)-O-Î’-D-Glucuronopyranosyl]-24-Hydroxyglabrolide | Gan Cao |
| Glyarallin B | Gan Cao |
| Glycycoumarin | Gan Cao |
| Glycyrin | Gan Cao |
| Glycyrol | Gan Cao |
| Glycyrrhetinic Acid | Gan Cao |
| Glycyrrhetol | Gan Cao |
| Glycyrrhisoflavanone | Gan Cao |
| Glycyrrhisoflavone | Gan Cao |
| Glycyrrhiza-Flavonol A | Gan Cao |
| Glycyrrhizic Acid | Gan Cao |
| Glyurallin A | Gan Cao |
| Glyuranolide | Gan Cao |
| Glyzaglabrin | Gan Cao |
| Hispaglabridin A | Gan Cao |
| Hispaglabridin B | Gan Cao |
| 3-Hydroxyglabrol | Gan Cao |
| Isoglycyrol | Gan Cao |
| Isolicoflavonol | Gan Cao |
| Isoliquiritigenin | Gan Cao |
| Isoliquiritin | Gan Cao |
| Isoononin | Gan Cao |
| Isotrifoliol | Gan Cao |
| Kanzonol K | Gan Cao |
| Kanzonol L | Gan Cao |
| Licobenzofuran | Gan Cao |
| Licobichalcone | Gan Cao |
| Licocoumarone | Gan Cao |
| Licofuranocoumarin | Gan Cao |
| Licoisoflavone | Gan Cao |
| Licoleafol | Gan Cao |
| Licopyranocoumarin | Gan Cao |
| Licoricesaponin A3 | Gan Cao |
| Licoricesaponin B2 | Gan Cao |
| Licoricesaponin C2 | Gan Cao |
| Licoricesaponin D3 | Gan Cao |
| Licoricesaponin E2 | Gan Cao |
| Licoricesaponin F3 | Gan Cao |
| Licoricesaponin G2 | Gan Cao |
| Licoricesaponin H2 | Gan Cao |
| Licoricesaponin J2 | Gan Cao |
| Licoricesaponin K2 | Gan Cao |
| Licoricidin | Gan Cao |
| Licoricone | Gan Cao |
| Licorisoflavan A | Gan Cao |
| Liquiritigenin | Gan Cao |
| Liquiritigenin 4'-O-Î’-D-Apio-D-Furanosyl(1â†’2)-Î’-D-Glucopyranoside | Gan Cao |
| Liquiritigenin-7,4'-Diglucoside | Gan Cao |
| Liquiritin | Gan Cao |
| 3'-Methoxyglabridin | Gan Cao |
| Methyl-N-Butyl-Uralsaponin A Esters | Gan Cao |
| 4'-O-Methylglabridin | Gan Cao |
| Methylglycyrrhetate | Gan Cao |
| Methyl-24-Hydroxy-11-Deoxoglycyrrhetate | Gan Cao |
| Methyl 18Î‘-Hydroxyglycyrrhetate | Gan Cao |
| Methyl-24-Hydroxyglycyrrhetate | Gan Cao |
| 3-Methyl-6,7,8-Trihydropyrrolo[1,2-A]Pyrimidin-2-One | Gan Cao |
| Narcissin | Gan Cao |
| Neoisoliquiritin | Gan Cao |
| Neoliquiritin | Gan Cao |
| Neouralenol | Gan Cao |
| Phaseollinisoflavan | Gan Cao |
| Sigmoidin B | Gan Cao |
| 5,6,7,8-Tetrahydro-2,4-Dimethylquinoline | Gan Cao |
| 5,6,7,8-Tetrahydro-4-Methylquinoline | Gan Cao |
| N-Tricosane | Gan Cao |
| 2,4,4'-Trihydroxychalcone | Gan Cao |
| Uralene | Gan Cao |
| Uralenin | Gan Cao |
| Uralenneoside | Gan Cao |
| Uralenol | Gan Cao |
| Uralenol-3-Methylether | Gan Cao |
| Uralsaponin A | Gan Cao |
| Uralsaponin B | Gan Cao |
| Uralstilbene | Gan Cao |
| Kanzonol B | Gan Cao |
| Choerospondin | Gan Cao |
| 3',7-Dihydroxy-4',6-Dimethoxyisoflavone | Gan Cao |
| [6',6''-Dimethylpyrano-(2',3':7,8)]-4'-Methoxy-3-Arylcoumarin | Gan Cao |
| Erythrinin C | Gan Cao |
| Gancaonin Y | Gan Cao |
| Gancaonin Z | Gan Cao |
| Glicoricone | Gan Cao |
| Glycyrdione D | Gan Cao |
| 4-Hydroxyonchocarpin | Gan Cao |
| Isograbrol | Gan Cao |
| Isoschaftoside | Gan Cao |
| Kumatakenin | Gan Cao |
| Licoagrochalcone A | Gan Cao |
| Licoagroisoflavone | Gan Cao |
| Licoagropin | Gan Cao |
| Licoagroside C | Gan Cao |
| Licoarylcoumarin | Gan Cao |
| Licoflavonal | Gan Cao |
| Licuroside | Gan Cao |
| Maackiain | Gan Cao |
| Ordoritin-Glucoside | Gan Cao |
| Phaseol | Gan Cao |
| 6-Prenylated Eriodictyol | Gan Cao |
| 8-Prenylated Eriodictyol | Gan Cao |
| Semilicoisoflavone B | Gan Cao |
| Violanthin | Gan Cao |
| Xambioona | Gan Cao |
| Isoviolanthin | Gan Cao |
| Licoisoflavone B | Gan Cao |

| **Table1:** **137 active compounds from databases and literature in Sijunzi Decoction** | | | |
| --- | --- | --- | --- |
| **Mol ID** | **Molecule Name** | **OB** | **DL** |
| MOL002879 | Diop | 43.59 | 0.39 |
| MOL000449 | Stigmasterol | 43.83 | 0.76 |
| MOL000358 | beta-sitosterol | 36.91 | 0.75 |
| MOL003648 | Inermin | 65.83 | 0.54 |
| MOL000422 | kaempferol | 41.88 | 0.24 |
| MOL004492 | Chrysanthemaxanthin | 38.72 | 0.58 |
| MOL005308 | Aposiopolamine | 66.65 | 0.22 |
| MOL005314 | Celabenzine | 101.88 | 0.49 |
| MOL005317 | Deoxyharringtonine | 39.27 | 0.81 |
| MOL005318 | Dianthramine | 40.45 | 0.2 |
| MOL005320 | arachidonate | 45.57 | 0.2 |
| MOL005321 | Frutinone A | 65.9 | 0.34 |
| MOL005344 | ginsenoside rh2 | 36.32 | 0.56 |
| MOL005348 | Ginsenoside-Rh4_qt | 31.11 | 0.78 |
| MOL005356 | Girinimbin | 61.22 | 0.31 |
| MOL005357 | Gomisin B | 31.99 | 0.83 |
| MOL005360 | malkangunin | 57.71 | 0.63 |
| MOL005376 | Panaxadiol | 33.09 | 0.79 |
| MOL005384 | suchilactone | 57.52 | 0.56 |
| MOL005399 | alexandrin_qt | 36.91 | 0.75 |
| MOL005401 | ginsenoside Rg5_qt | 39.56 | 0.79 |
| MOL000787 | Fumarine | 59.26 | 0.83 |
| MOL000020 | 12-senecioyl-2E,8E,10E-atractylentriol | 62.4 | 0.22 |
| MOL000021 | 14-acetyl-12-senecioyl-2E,8E,10E-atractylentriol | 60.31 | 0.31 |
| MOL000022 | 14-acetyl-12-senecioyl-2E,8Z,10E-atractylentriol | 63.37 | 0.3 |
| MOL000028 | α-Amyrin | 39.51 | 0.76 |
| MOL000033 | (3S,8S,9S,10R,13R,14S,17R)-10,13-dimethyl-17-[(2R,5S)-5-propan-2-yloctan-2-yl]-2,3,4,7,8,9,11,12,14,15,16,17-dodecahydro-1H-cyclopenta[a]phenanthren-3-ol | 36.23 | 0.78 |
| MOL000049 | 3β-acetoxyatractylone | 54.07 | 0.22 |
| MOL000072 | 8β-ethoxy atractylenolide Ⅲ | 35.95 | 0.21 |
| MOL000273 | (2R)-2-[(3S,5R,10S,13R,14R,16R,17R)-3,16-dihydroxy-4,4,10,13,14-pentamethyl-2,3,5,6,12,15,16,17-octahydro-1H-cyclopenta[a]phenanthren-17-yl]-6-methylhept-5-enoic acid | 30.93 | 0.81 |
| MOL000275 | trametenolic acid | 38.71 | 0.8 |
| MOL000276 | 7,9(11)-dehydropachymic acid | 35.11 | 0.81 |
| MOL000279 | Cerevisterol | 37.96 | 0.77 |
| MOL000280 | (2R)-2-[(3S,5R,10S,13R,14R,16R,17R)-3,16-dihydroxy-4,4,10,13,14-pentamethyl-2,3,5,6,12,15,16,17-octahydro-1H-cyclopenta[a]phenanthren-17-yl]-5-isopropyl-hex-5-enoic acid | 31.07 | 0.82 |
| MOL000282 | ergosta-7,22E-dien-3beta-ol | 43.51 | 0.72 |
| MOL000283 | Ergosterol peroxide | 40.36 | 0.81 |
| MOL000285 | (2R)-2-[(5R,10S,13R,14R,16R,17R)-16-hydroxy-3-keto-4,4,10,13,14-pentamethyl-1,2,5,6,12,15,16,17-octahydrocyclopenta[a]phenanthren-17-yl]-5-isopropyl-hex-5-enoic acid | 38.26 | 0.82 |
| MOL000287 | 3beta-Hydroxy-24-methylene-8-lanostene-21-oic acid | 38.7 | 0.81 |
| MOL000289 | pachymic acid | 33.63 | 0.81 |
| MOL000290 | Poricoic acid A | 30.61 | 0.76 |
| MOL000291 | Poricoic acid B | 30.52 | 0.75 |
| MOL000292 | poricoic acid C | 38.15 | 0.75 |
| MOL000296 | hederagenin | 36.91 | 0.75 |
| MOL000300 | dehydroeburicoic acid | 44.17 | 0.83 |
| MOL001484 | Inermine | 75.18 | 0.54 |
| MOL001792 | DFV | 32.76 | 0.18 |
| MOL001789 | isoliquiritigenin | 85.32 | 0.15 |
| MOL000211 | Mairin | 55.38 | 0.78 |
| MOL002311 | Glycyrol | 90.78 | 0.67 |
| MOL000239 | Jaranol | 50.83 | 0.29 |
| MOL002565 | Medicarpin | 49.22 | 0.34 |
| MOL000354 | isorhamnetin | 49.6 | 0.31 |
| MOL000359 | sitosterol | 36.91 | 0.75 |
| MOL003656 | Lupiwighteone | 51.64 | 0.37 |
| MOL003896 | 7-Methoxy-2-methyl isoflavone | 42.56 | 0.2 |
| MOL000392 | formononetin | 69.67 | 0.21 |
| MOL000417 | Calycosin | 47.75 | 0.24 |
| MOL000422 | kaempferol | 41.88 | 0.24 |
| MOL004328 | naringenin | 59.29 | 0.21 |
| MOL004805 | (2S)-2-[4-hydroxy-3-(3-methylbut-2-enyl)phenyl]-8,8-dimethyl-2,3-dihydropyrano[2,3-f]chromen-4-one | 31.79 | 0.72 |
| MOL004806 | euchrenone | 30.29 | 0.57 |
| MOL004808 | glyasperin B | 65.22 | 0.44 |
| MOL004810 | glyasperin F | 75.84 | 0.54 |
| MOL004811 | Glyasperin C | 45.56 | 0.4 |
| MOL004814 | Isotrifoliol | 31.94 | 0.42 |
| MOL004815 | (E)-1-(2,4-dihydroxyphenyl)-3-(2,2-dimethylchromen-6-yl)prop-2-en-1-one | 39.62 | 0.35 |
| MOL004820 | kanzonols W | 50.48 | 0.52 |
| MOL004824 | (2S)-6-(2,4-dihydroxyphenyl)-2-(2-hydroxypropan-2-yl)-4-methoxy-2,3-dihydrofuro[3,2-g]chromen-7-one | 60.25 | 0.63 |
| MOL004827 | Semilicoisoflavone B | 48.78 | 0.55 |
| MOL004828 | Glepidotin A | 44.72 | 0.35 |
| MOL004829 | Glepidotin B | 64.46 | 0.34 |
| MOL004833 | Phaseolinisoflavan | 32.01 | 0.45 |
| MOL004835 | Glypallichalcone | 61.6 | 0.19 |
| MOL004838 | 8-(6-hydroxy-2-benzofuranyl)-2,2-dimethyl-5-chromenol | 58.44 | 0.38 |
| MOL004841 | Licochalcone B | 76.76 | 0.19 |
| MOL004848 | licochalcone G | 49.25 | 0.32 |
| MOL004849 | 3-(2,4-dihydroxyphenyl)-8-(1,1-dimethylprop-2-enyl)-7-hydroxy-5-methoxy-coumarin | 59.62 | 0.43 |
| MOL004855 | Licoricone | 63.58 | 0.47 |
| MOL004856 | Gancaonin A | 51.08 | 0.4 |
| MOL004857 | Gancaonin B | 48.79 | 0.45 |
| MOL004860 | licorice glycoside E | 32.89 | 0.27 |
| MOL004863 | 3-(3,4-dihydroxyphenyl)-5,7-dihydroxy-8-(3-methylbut-2-enyl)chromone | 66.37 | 0.41 |
| MOL004864 | 5,7-dihydroxy-3-(4-methoxyphenyl)-8-(3-methylbut-2-enyl)chromone | 30.49 | 0.41 |
| MOL004866 | 2-(3,4-dihydroxyphenyl)-5,7-dihydroxy-6-(3-methylbut-2-enyl)chromone | 44.15 | 0.41 |
| MOL004879 | Glycyrin | 52.61 | 0.47 |
| MOL004882 | Licocoumarone | 33.21 | 0.36 |
| MOL004883 | Licoisoflavone | 41.61 | 0.42 |
| MOL004884 | Licoisoflavone B | 38.93 | 0.55 |
| MOL004885 | licoisoflavanone | 52.47 | 0.54 |
| MOL004891 | shinpterocarpin | 80.3 | 0.73 |
| MOL004898 | (E)-3-[3,4-dihydroxy-5-(3-methylbut-2-enyl)phenyl]-1-(2,4-dihydroxyphenyl)prop-2-en-1-one | 46.27 | 0.31 |
| MOL004903 | liquiritin | 65.69 | 0.74 |
| MOL004904 | licopyranocoumarin | 80.36 | 0.65 |
| MOL004905 | 3,22-Dihydroxy-11-oxo-delta(12)-oleanene-27-alpha-methoxycarbonyl-29-oic acid | 34.32 | 0.55 |
| MOL004907 | Glyzaglabrin | 61.07 | 0.35 |
| MOL004908 | Glabridin | 53.25 | 0.47 |
| MOL004910 | Glabranin | 52.9 | 0.31 |
| MOL004911 | Glabrene | 46.27 | 0.44 |
| MOL004912 | Glabrone | 52.51 | 0.5 |
| MOL004913 | 1,3-dihydroxy-9-methoxy-6-benzofurano[3,2-c]chromenone | 48.14 | 0.43 |
| MOL004914 | 1,3-dihydroxy-8,9-dimethoxy-6-benzofurano[3,2-c]chromenone | 62.9 | 0.53 |
| MOL004915 | Eurycarpin A | 43.28 | 0.37 |
| MOL004917 | glycyroside | 37.25 | 0.79 |
| MOL004924 | (-)-Medicocarpin | 40.99 | 0.95 |
| MOL004935 | Sigmoidin-B | 34.88 | 0.41 |
| MOL004941 | (2R)-7-hydroxy-2-(4-hydroxyphenyl)chroman-4-one | 71.12 | 0.18 |
| MOL004945 | (2S)-7-hydroxy-2-(4-hydroxyphenyl)-8-(3-methylbut-2-enyl)chroman-4-one | 36.57 | 0.32 |
| MOL004948 | Isoglycyrol | 44.7 | 0.84 |
| MOL004949 | Isolicoflavonol | 45.17 | 0.42 |
| MOL004957 | HMO | 38.37 | 0.21 |
| MOL004959 | 1-Methoxyphaseollidin | 69.98 | 0.64 |
| MOL004961 | Quercetin der. | 46.45 | 0.33 |
| MOL004966 | 3'-Hydroxy-4'-O-Methylglabridin | 43.71 | 0.57 |
| MOL000497 | licochalcone a | 40.79 | 0.29 |
| MOL004974 | 3'-Methoxyglabridin | 46.16 | 0.57 |
| MOL004978 | 2-[(3R)-8,8-dimethyl-3,4-dihydro-2H-pyrano[6,5-f]chromen-3-yl]-5-methoxyphenol | 36.21 | 0.52 |
| MOL004980 | Inflacoumarin A | 39.71 | 0.33 |
| MOL004985 | icos-5-enoic acid | 30.7 | 0.2 |
| MOL004988 | Kanzonol F | 32.47 | 0.89 |
| MOL004989 | 6-prenylated eriodictyol | 39.22 | 0.41 |
| MOL004990 | 7,2',4'-trihydroxy－5-methoxy-3－arylcoumarin | 83.71 | 0.27 |
| MOL004991 | 7-Acetoxy-2-methylisoflavone | 38.92 | 0.26 |
| MOL004993 | 8-prenylated eriodictyol | 53.79 | 0.4 |
| MOL004996 | gadelaidic acid | 30.7 | 0.2 |
| MOL000500 | Vestitol | 74.66 | 0.21 |
| MOL005000 | Gancaonin G | 60.44 | 0.39 |
| MOL005001 | Gancaonin H | 50.1 | 0.78 |
| MOL005003 | Licoagrocarpin | 58.81 | 0.58 |
| MOL005007 | Glyasperins M | 72.67 | 0.59 |
| MOL005008 | Glycyrrhiza flavonol A | 41.28 | 0.6 |
| MOL005012 | Licoagroisoflavone | 57.28 | 0.49 |
| MOL005013 | 18α-hydroxyglycyrrhetic acid | 41.16 | 0.71 |
| MOL005016 | Odoratin | 49.95 | 0.3 |
| MOL005017 | Phaseol | 78.77 | 0.58 |
| MOL005018 | Xambioona | 54.85 | 0.87 |
| MOL005020 | dehydroglyasperins C | 53.82 | 0.37 |
| MOL000098 | quercetin | 46.43 | 0.28 |
