# Supplementary material for: Integrated Network Pharmacology and Gut Microbiota Analysis to Explore the Mechanism of Sijunzi Decoction Involved in Alleviating Airway Inflammation in a Mouse Model of Asthma
Source: Evid Based Complement Alternat Med. 2023 Jan 3;2023:1130893. doi: 10.1155/2023/1130893 (PMC9831717; doi:10.1155/2023/1130893)
Supplement: Supplementary Materials — Supplementary Table 1: active compounds from databases and literature in Sijunzi decoction. Supplementary Table 2: asthma-related genes in the database. Supplementary Table 3: common genes of asthma and Sijunzi decoction. Supplementary Table 4: GO functional categories. Supplementary Table 5: data of KEGG enrichment analysis. [file 1130893.f1.zip › Supplementary Table 2.docx]

| **Supplementary Table 2: asthma related genes in the database** | | | | |
| --- | --- | --- | --- | --- |
| A total of 1445 genes associated with asthma were obtained from the four databases | Information about asthma diseas in various databases | | | |
|  | OMIM | DRUGBANK | TTD | GeneCard |
| VDR | VDR | PDE3A | VIPR | DPP10 |
| 5-LOX | USP38 | ADORA3 | UTS2R | MMP9 |
| ABCA12 | TYRO3 | ADORA1 | TYK2 | HLA-DQB1 |
| ABCA3 | TRBC1 | CYP1A2 | TSLP | CCL17 |
| ABCB1 | TNIP1 | CYP2E1 | Tryptase | CCL26 |
| ABCB11 | TNF | CYP3A4 | TRPV1 | NPSR1-AS1 |
| ABCC1 | TLR4 | HDAC2 | TOP2 | MPO |
| ABCC2 | TBX21 | ADRB2 | TNFR | ITGAM |
| ABCG2 | STAT6 | CYP2D6 | TNF | ALOX15 |
| ACE | SPRED1 | CYP2C19 | TLR9 | CCL24 |
| ACKR3 | SOCS3 | CYP2A6 | TLR7 | IL16 |
| ACP1 | SELP | CYP2C9 | TBXAS1 | CD69 |
| ACTA2 | RUNX3 | SERPINA6 | TBXA2R | ITK |
| ADA | RASGRP4 | NR3C1 | TACR3 | TAC1 |
| ADAM33 | PTGDR2 | CYP3A5 | TACR2 | IL1A |
| ADAM8 | PTGDR | ABCB1 | TACR1 | IL1RL1 |
| ADAMTSL1 | PTEN | ABCG2 | SYK mRNA | IL1R1 |
| ADCY10 | POSTN | SLCO1B1 | SYK | GSTM1 |
| ADCY3 | PHF11 | SLCO1B3 | SERPINE1 | CCL2 |
| ADCY9 | ORMDL3 | ABCB11 | SELP | CCR3 |
| ADCYAP1 | NPSRAS1 | ANXA1 | SELL | IL1RN |
| ADIPOQ | NPSR1 | Cyp3a2 | SELE | PDE4A |
| ADM | NOD1 | CYP1B1 | SEL | CD40LG |
| ADORA1 | MMP9 | CYP2B6 | SCN9A | KITLG |
| ADORA1 mRNA | MMP2 | CYP2C8 | SCN10A | ALOX5AP |
| ADORA2A | MMP12 | SLCO1A2 | ROS | TLR6 |
| ADORA2B | MIR126 | SLC22A8 | ROCK | CCL22 |
| ADORA3 | MARCKS | ALB | PTK | BDNF |
| ADRA1A | LTC4S | HRH1 | PTGIR | PTGDR2 |
| ADRA1B | LTA | SLC6A4 | PTGER4 | HLA-DPB1 |
| ADRA1D | IRF4 | SLC6A2 | PTGER3 | ELANE |
| ADRA2A | IRAK3 | SLC6A3 | PTGDR2 | LEP |
| ADRA2B | IL9R | CYP3A7 | PTGDR | CAT |
| ADRA2C | IL9 | SLC22A2 | PTAFR | EDN1 |
| ADRB1 | IL6R | SLC22A1 | PRSS1 | GSDMB |
| ADRB2 | IL5 | CES1 | PLA2G1B | FCER1A |
| ADRB3 | IL4R | S100P | PK | HLA-DQA1 |
| ADSL | IL4 | NOS2 | PDE5A | SELP |
| AFF4 | IL13 | NR0B1 | PDE4D | VIP |
| AGER | IL12B | ABCC2 | PDE4B | SELE |
| AGPS | IL10 | CYP17A1 | PDE4A | ITGB2 |
| AGT | HNMT | CYP1A1 | PDE4 | NLRP3 |
| AGTR1 | HAVCR1 | CYP3A43 | PDE3A | VDR |
| AHI1 | GLCCI1 | CYP4A11 | PDE3 | MUC5AC |
| AICDA | GATA3 | NR1I2 | PDE | CXCL10 |
| AIF1 | GAD2 | CYP11B1 | p38 | SELL |
| AKR1B1 | GAD1 | HSD11B2 | OX40 | CSF3 |
| AKR1C1 | FLG | HSD11B1 | OPR | COL26A1 |
| AKR1C3 | FKBP5 | ADRA1A | NR3C1 | ITGA4 |
| AKR1D1 | DPP10 | ADRB1 | NOS2 | CDHR3 |
| AKT1 | DENND1B | ADRA1B | NFKB | TLR9 |
| ALAD | DAP3 | ADRA1D | nAChR | FCER2 |
| ALB | CYSLTR2 | ADRA2A | MycB mmpL3 | CXCR3 |
| ALDH18A1 | CYSLTR1 | ADRA2B | MPIP1 | CSF1 |
| ALDH1A2 | CMA1 | TNF | MOP | NOS2 |
| ALDH2 | CLCA1 | COMT | MMP-12 | TBXA2R |
| ALDH3A2 | CHI3L1 | MAOA | MME | DENND1B |
| ALMS1 | CFTR | PGR | MCL1 | IL18R1 |
| ALOX12B | C5AR1 | PLA2G4A | MC4R | NPY |
| ALOX15 | C5 | NR3C2 | MAP3K9 | FOXP3 |
| ALOX5 | C3AR1 | ADRB3 | M1 IgE | PRG2 |
| ALOX5 mRNA | C3 | UGT1A1 | LTR | KNG1 |
| ALOX5AP | ALOX5AP | UGT1A8 | LTB4R2 | CCR4 |
| ALOXE3 | ALOX5 | UGT1A9 | LTB4R | NOD1 |
| ANGPT1 | ADCY9 | UGT2B7 | LT synth | IRF1 |
| ANGPT2 | ADAM33 | UGT2B15 | L-CaC | TLR10 |
| ANXA1 | TPT1 | SLC22A4 | KCNMA1 | BGLAP |
| ANXA2 | TNFSF14 | SLC22A5 | KC | POSTN |
| ANXA5 | TNFSF10 | SLC22A3 | JAK-3 | LTA4H |
| AOAH | TNFRSF4 | GRIN1 | JAK-2 | FKBP5 |
| AOC1 | TBXA2R | SHBG | JAK-1 | IL5RA |
| AOC3 | SART1 | CYP11B2 | ITGA5 | HLA-DRA |
| APC | PTGER2 | AKR1D1 | ITGA4/B1 | NOS1 |
| APEX1 | PRKCA | SRD5A2 | ITGA4 | IL9R |
| APOA1 | PLA2G7 | CHRM1 | IsoM | NGF |
| APOH | NFKBIA | CHRM2 | IL9 | GLCCI1 |
| AQP3 | MUC7 | CHRM3 | IL5RA | CCL7 |
| AQP5 | IL13RA2 | ORM1 | IL5 | CALCA |
| AREG | IL13RA1 | GLB1 | IL4R | RASGRP4 |
| ARG1 | HSD11B2 | GLT6D1 | IL4 | ADAM8 |
| ARG2 | HLA-G | GLTP | IL33 | PPBP |
| ARL6 | GABRB2 | xlnA | IL23 | SOD2 |
| ARMC4 | GABRA2 | LGALS3 | IL17R | TIMP1 |
| ARRB2 | CHIA | nedA | IL17 | TRPV1 |
| ARSB | SPINK5 | AKR1C1 | IL13RA2 | IL31 |
| ARVCF | SETDB2 | CYSLTR1 | IL-1 synth/rele | BMP6 |
| ASCC1 | SERPINB4 | ALOX5 | IgE | FAS |
| ASOBS | SERPINB3 | SLCO2B1 | HSP90 | MMP2 |
| ASXL1 | SERPINB2 | PTGS1 | HSP20 | ALB |
| ATAT1 | SCGB3A2 | CYSLTR2 | HR | HLA-DPA1 |
| ATF6 | SCGB1A1 | PTGDR | His/p-LT rele | PF4 |
| ATF6B | MS4A2 | FPR1 | His rele | SFTPD |
| ATG3 | HLA-DRB1 | HSP90AA1 | H4R | IL17F |
| ATG5 | CPN1 | PDE4A | H1R | NPPA |
| ATIII | CCR5 | ADORA2A | GS | CCR6 |
| ATOD1 | CCL24 | SLC22A7 | GR | TPT1 |
| ATOD3 | CCL18 | IL5 | GIVA cPLA2 | POMC |
| ATOD5 | CCL11 | RNASE3 | FLAP | HLA-DQA2 |
| ATOD6 | BPIFA1 | NFKB1 | FCERG | CD40 |
| ATOD7 | LGALS3 | MUC2 | F2 | TPSAB1 |
| ATOD8 | FCER1A | ADRA2C | ESRRA | TLR3 |
| ATOD9 | FCER1A | SLC25A21 | ELR accum | MMP1 |
| ATP12A |  | SLC13A2 | D2R | CREB1 |
| ATP2A2 |  | SLC13A5 | CYSLTR2 | ADIPOQ |
| ATP4A |  | SLC25A1 | CYSLTR1 | JUN |
| ATP7A |  | BCHE | CYP26A1 | CD79A |
| ATXN2 |  | SULT2A1 | CXCR2 | F2RL1 |
| AVPR1B |  | PDE4B | CTSS | SYK |
| BAX |  | ADORA2B | CTSL | CXCL1 |
| BBIP1 |  | PDE5A | CTSK | TLR1 |
| BBS1 |  | ADA | CTSB | CDSN |
| BBS10 |  | CPNE1 | CSF2RB | FASLG |
| BBS12 |  | HM13 | COX-2 | TACR1 |
| BBS2 |  | NOMO1 | COX | FGFBP2 |
| BBS4 |  | PARP1 | CHRNA7 | JAK2 |
| BBS5 |  | RIC3 | CHRM5 | IL6R |
| BBS7 |  | TTLL3 | CHRM4 | SOCS3 |
| BBS9 |  | CHRM4 | CHRM3 | CPA3 |
| BCHE |  | CHRM5 | CHRM2 | FCER1G |
| BCL11B |  | PTGS2 | CHRM | ARG1 |
| BCL2 |  |  | CHR | CCL13 |
| BCL6 |  |  | CFB | PTGS1 |
| BDKRB2 |  |  | CCR4 | CHIT1 |
| BDNF |  |  | CCR3 | IL11 |
| BGLAP |  |  | CCL2 | HLA-DOA |
| BHLHE40 |  |  | CBR1 | PLA2G2A |
| BKbeta4 |  |  | CAMP | CD4 |
| BMP4 |  |  | CALC | SLC6A4 |
| BMP6 |  |  | CA-IV | PLAU |
| BPI |  |  | BKbeta4 | GSR |
| BPIFA1 |  |  | BDKRB2 | RAPGEF3 |
| BRD2 |  |  | ATP2A2 | CXCL12 |
| BSG |  |  | ATIII | CRH |
| BST2 |  |  | AOC3 | CYP3A4 |
| BTD |  |  | ANGPT2 | CMA1 |
| BTNL2 |  |  | ANGPT1 | CP |
| C19orf12 |  |  | ALOX5 mRNA | MAPK3 |
| C3 |  |  | ADRB3 | F2R |
| C3AR1 |  |  | ADRB2 | CXCR4 |
| C4A |  |  | ADRB1 | CD86 |
| C5 |  |  | ADRA2C | CD44 |
| C5AR1 |  |  | ADORA2B | G6PD |
| C9orf24 |  |  | ADORA1 mRNA | AREG |
| CABIN1 |  |  | ACKR3 | ADCY10 |
| CACNG6 |  |  | ACE | HDAC2 |
| CA-IV |  |  | 5-LOX | ABCB1 |
| CALC |  |  |  | CHRM3 |
| CALCA |  |  |  | CXCL5 |
| CALCOCO2 |  |  |  | CXCR1 |
| CALR |  |  |  | IL12RB2 |
| CAMP |  |  |  | PIK3CG |
| CANX |  |  |  | ADM |
| CARD11 |  |  |  | ADRA1B |
| CARMIL2 |  |  |  | NFKBIA |
| CASP1 |  |  |  | ARG2 |
| CASP10 |  |  |  | MMP12 |
| CASP14 |  |  |  | ABCC1 |
| CASR |  |  |  | CCL4 |
| CAT |  |  |  | CX3CR1 |
| CATSPER1 |  |  |  | CRHR1 |
| CAV1 |  |  |  | VTN |
| CBL |  |  |  | IL13RA1 |
| CBR1 |  |  |  | SOCS1 |
| CCDC103 |  |  |  | THPO |
| CCDC114 |  |  |  | GHRL |
| CCDC151 |  |  |  | CAMP |
| CCDC28B |  |  |  | F3 |
| CCDC39 |  |  |  | HAVCR2 |
| CCDC40 |  |  |  | PPARG |
| CCDC65 |  |  |  | AKR1B1 |
| CCHCR1 |  |  |  | OPRM1 |
| CCK |  |  |  | ADAMTSL1 |
| CCKBR |  |  |  | CLC |
| CCL1 |  |  |  | GAL |
| CCL11 |  |  |  | TNFRSF10A |
| CCL13 |  |  |  | IL13RA2 |
| CCL17 |  |  |  | CXCL9 |
| CCL18 |  |  |  | ITGAL |
| CCL2 |  |  |  | C5AR1 |
| CCL20 |  |  |  | TNFSF10 |
| CCL22 |  |  |  | SERPINA1 |
| CCL24 |  |  |  | TACR2 |
| CCL26 |  |  |  | TF |
| CCL27 |  |  |  | HP |
| CCL4 |  |  |  | ADCY9 |
| CCL7 |  |  |  | STAT5A |
| CCND1 |  |  |  | CD28 |
| CCNO |  |  |  | IGFBP3 |
| CCR10 |  |  |  | ACP1 |
| CCR2 |  |  |  | F2 |
| CCR3 |  |  |  | LPO |
| CCR4 |  |  |  | PDE4D |
| CCR5 |  |  |  | AGTR1 |
| CCR6 |  |  |  | SP1 |
| CCR7 |  |  |  | NQO1 |
| CCR8 |  |  |  | CD80 |
| CD177 |  |  |  | ITGA2 |
| CD2 |  |  |  | CTSG |
| CD209 |  |  |  | PTGER4 |
| CD247 |  |  |  | NFE2L2 |
| CD27 |  |  |  | P2RY12 |
| CD274 |  |  |  | F2RL3 |
| CD28 |  |  |  | FGA |
| CD34 |  |  |  | INS |
| CD36 |  |  |  | ADRB1 |
| CD4 |  |  |  | ICOSLG |
| CD40 |  |  |  | HRH2 |
| CD40LG |  |  |  | SST |
| CD44 |  |  |  | PITX2 |
| CD63 |  |  |  | CCR7 |
| CD69 |  |  |  | CYP2C9 |
| CD79A |  |  |  | MMRN1 |
| CD80 |  |  |  | MME |
| CD83 |  |  |  | LAT |
| CD86 |  |  |  | CLCA1 |
| CD8A |  |  |  | GNRH1 |
| CD9 |  |  |  | PLA2G4A |
| CDC5L |  |  |  | RYR2 |
| CDH1 |  |  |  | CCR8 |
| CDH26 |  |  |  | GRK5 |
| CDHR3 |  |  |  | ADCYAP1 |
| CDKN3 |  |  |  | SLC22A2 |
| CDON |  |  |  | ADORA2B |
| CDSN |  |  |  | ADORA1 |
| CDX2 |  |  |  | CXCR2 |
| CEACAM5 |  |  |  | SERPINA3 |
| CEBPB |  |  |  | IL7 |
| CEP290 |  |  |  | GZMB |
| CEP295 |  |  |  | LTB4R |
| CEP83 |  |  |  | EIF4E |
| CEP89 |  |  |  | MAPK14 |
| CES1 |  |  |  | KLK3 |
| CETN2 |  |  |  | C3AR1 |
| CFAP221 |  |  |  | RNASE2 |
| CFAP298 |  |  |  | ADORA2A |
| CFAP300 |  |  |  | SLC22A3 |
| CFAP54 |  |  |  | IFNB1 |
| CFB |  |  |  | CCL1 |
| CFHR2 |  |  |  | MB |
| CFLAR |  |  |  | HBG2 |
| CFTR |  |  |  | ADRB3 |
| CHAT |  |  |  | EGR1 |
| CHD6 |  |  |  | GAPDH |
| CHD7 |  |  |  | SERPINA6 |
| CHGA |  |  |  | CYP3A5 |
| CHI3L1 |  |  |  | SDHB |
| CHIA |  |  |  | TNFSF13B |
| CHIC2 |  |  |  | TNFRSF4 |
| CHIT1 |  |  |  | SELPLG |
| CHR |  |  |  | EPRS1 |
| CHRM |  |  |  | PVT1 |
| CHRM1 |  |  |  | ADORA3 |
| CHRM2 |  |  |  | BCL11B |
| CHRM3 |  |  |  | SHH |
| CHRM4 |  |  |  | COL5A1 |
| CHRM5 |  |  |  | NEK9 |
| CHRNA7 |  |  |  | COL5A2 |
| CHUK |  |  |  | FGFR1 |
| CILD4 |  |  |  | BBS1 |
| CILD8 |  |  |  | NKX2-1 |
| CLC |  |  |  | PLCG2 |
| CLCA1 |  |  |  | CARD11 |
| CLCA4 |  |  |  | DOCK8 |
| CLCN2 |  |  |  | FOXJ1 |
| CLCN3 |  |  |  | CPN1 |
| CLCN5 |  |  |  | ATOD3 |
| CLEC16A |  |  |  | DDX41 |
| CLEC1A |  |  |  | LTA |
| CLEC7A |  |  |  | KIT |
| CLIC2 |  |  |  | PDGFRA |
| CMA1 |  |  |  | ACE |
| CNTLN |  |  |  | TGIF1 |
| CNTN2 |  |  |  | GRHL2 |
| COL1A1 |  |  |  | COL1A1 |
| COL1A2 |  |  |  | ATOD5 |
| COL26A1 |  |  |  | ATOD6 |
| COL2A1 |  |  |  | FSIP1 |
| COL5A1 |  |  |  | FGF8 |
| COL5A2 |  |  |  | ELOVL4 |
| COL6A5 |  |  |  | PEX5 |
| COMT |  |  |  | NODAL |
| COTL1 |  |  |  | GP1BB |
| COX |  |  |  | ALMS1 |
| COX-2 |  |  |  | GTF2H5 |
| COX4I2 |  |  |  | RPGR |
| COX5A |  |  |  | TRAIP |
| CP |  |  |  | CCDC151 |
| CPA3 |  |  |  | CARMIL2 |
| CPN1 |  |  |  | ATOD1 |
| CPNE1 |  |  |  | VEGFA |
| CPS1 |  |  |  | GP1BA |
| CPSF4 |  |  |  | WDR46 |
| CRB1 |  |  |  | HLA-B |
| CREB1 |  |  |  | SMAD3 |
| CREB5 |  |  |  | AFF4 |
| CREBBP |  |  |  | LIFR |
| CRH |  |  |  | POLR1H |
| CRHR1 |  |  |  | CREBBP |
| CRHR2 |  |  |  | STAT3 |
| CRLF2 |  |  |  | IL2RB |
| CRP |  |  |  | TBCK |
| CSF1 |  |  |  | ATOD7 |
| CSF2RB |  |  |  | ATOD8 |
| CSF3 |  |  |  | ATOD9 |
| CSN1S1 |  |  |  | GLI2 |
| CSN3 |  |  |  | GAS1 |
| CSTA |  |  |  | JMJD1C |
| CTCF |  |  |  | SPAG1 |
| CTHRC1 |  |  |  | ARMC4 |
| CTNNA3 |  |  |  | CCDC103 |
| CTNNB1 |  |  |  | SPEF2 |
| CTRL |  |  |  | PTGS2 |
| CTSB |  |  |  | NFKB2 |
| CTSE |  |  |  | LIG4 |
| CTSG |  |  |  | DNAH5 |
| CTSK |  |  |  | TGFBR1 |
| CTSL |  |  |  | MUC5B |
| CTSS |  |  |  | DNAH9 |
| CX3CL1 |  |  |  | TNFRSF8 |
| CX3CR1 |  |  |  | TGFB2 |
| CXCL1 |  |  |  | MTOR |
| CXCL10 |  |  |  | PEPD |
| CXCL11 |  |  |  | CLEC7A |
| CXCL12 |  |  |  | IL21R |
| CXCL5 |  |  |  | PTCH1 |
| CXCL9 |  |  |  | IDS |
| CXCR1 |  |  |  | STXBP1 |
| CXCR2 |  |  |  | ERCC2 |
| CXCR3 |  |  |  | TALDO1 |
| CXCR4 |  |  |  | USP7 |
| CXCR5 |  |  |  | GP9 |
| CYBA |  |  |  | CDON |
| CYBB |  |  |  | DLL1 |
| CYFIP2 |  |  |  | LMX1B |
| CYLD |  |  |  | SEC24C |
| CYP11B1 |  |  |  | SCN4A |
| CYP11B2 |  |  |  | SDHD |
| CYP17A1 |  |  |  | RREB1 |
| CYP1A1 |  |  |  | PGM3 |
| CYP1A2 |  |  |  | STK36 |
| CYP1B1 |  |  |  | SUFU |
| CYP21A2 |  |  |  | ARL6 |
| CYP26A1 |  |  |  | HIRA |
| CYP2A6 |  |  |  | SIX3 |
| CYP2B6 |  |  |  | NSUN2 |
| CYP2C19 |  |  |  | TBX1 |
| CYP2C8 |  |  |  | ZIC2 |
| CYP2C9 |  |  |  | CCNO |
| CYP2D6 |  |  |  | LRBA |
| CYP2E1 |  |  |  | FOXH1 |
| CYP2R1 |  |  |  | COX4I2 |
| Cyp3a2 |  |  |  | DNAI1 |
| CYP3A4 |  |  |  | DNAL1 |
| CYP3A43 |  |  |  | DNAH11 |
| CYP3A5 |  |  |  | OFD1 |
| CYP3A7 |  |  |  | SIK3 |
| CYP4A11 |  |  |  | TDGF1 |
| CYP4F22 |  |  |  | NME8 |
| CYSLTR1 |  |  |  | DNAI2 |
| CYSLTR2 |  |  |  | GAS8 |
| D2R |  |  |  | RSPH1 |
| DAB2 |  |  |  | ZMYND10 |
| DAP3 |  |  |  | ARVCF |
| DAW1 |  |  |  | RSPH4A |
| DCD |  |  |  | CCDC28B |
| DCDC2 |  |  |  | LRRC6 |
| DCLRE1C |  |  |  | DISP1 |
| DCTN4 |  |  |  | DNAAF2 |
| DDIT3 |  |  |  | DNAH1 |
| DDX39B |  |  |  | CCDC40 |
| DDX41 |  |  |  | RSPH9 |
| DDX58 |  |  |  | HYDIN |
| DEFB1 |  |  |  | CCDC114 |
| DEFB103A |  |  |  | CCDC65 |
| DEFB103B |  |  |  | LRRC56 |
| DEFB4A |  |  |  | DNAAF1 |
| DEL16P13.2 |  |  |  | DNAAF3 |
| DEL16P13.3 |  |  |  | DNAAF5 |
| DEL18Q |  |  |  | CCDC39 |
| DENND1B |  |  |  | RSPH3 |
| DGCR2 |  |  |  | DNAJB13 |
| DGCR6 |  |  |  | UFD1 |
| DGCR6L |  |  |  | TTC25 |
| DGCR8 |  |  |  | GAS2L2 |
| DGKE |  |  |  | DRC1 |
| DISP1 |  |  |  | DNAAF4 |
| DLL1 |  |  |  | CFAP298 |
| DNAAF1 |  |  |  | MCIDAS |
| DNAAF2 |  |  |  | DNAAF6 |
| DNAAF3 |  |  |  | CFAP221 |
| DNAAF4 |  |  |  | CFAP300 |
| DNAAF5 |  |  |  | MIR22 |
| DNAAF6 |  |  |  | IL15 |
| DNAH1 |  |  |  | TET2 |
| DNAH11 |  |  |  | CLEC1A |
| DNAH5 |  |  |  | CRP |
| DNAH7 |  |  |  | IFNA1 |
| DNAH8 |  |  |  | CRLF2 |
| DNAH9 |  |  |  | NPS |
| DNAI1 |  |  |  | SFTPA1 |
| DNAI2 |  |  |  | TGFBR2 |
| DNAJB13 |  |  |  | CCR5 |
| DNAJC3 |  |  |  | EP300 |
| DNAL1 |  |  |  | CCL18 |
| DNASE1 |  |  |  | FLG-AS1 |
| DNASE1L3 |  |  |  | IVL |
| DOCK8 |  |  |  | IL12RB1 |
| DPEP2 |  |  |  | CD8A |
| DPP10 |  |  |  | FBN1 |
| DPP4 |  |  |  | ASXL1 |
| DRC1 |  |  |  | IFITM3 |
| DRC7 |  |  |  | SRSF2 |
| DRD2 |  |  |  | CSN1S1 |
| DRD3 |  |  |  | S100A8 |
| DRD4 |  |  |  | SOD1 |
| DSC1 |  |  |  | FCGR3A |
| DSG1 |  |  |  | GSTT1 |
| DUPXQ28 |  |  |  | RIC1 |
| DUSP1 |  |  |  | CBL |
| DYNC2H1 |  |  |  | KRT14 |
| DYNLT1 |  |  |  | DNAH8 |
| EDIL3 |  |  |  | PRTN3 |
| EDN1 |  |  |  | PDCD1 |
| EFEMP2 |  |  |  | TLR7 |
| EGF |  |  |  | CX3CL1 |
| EGFR |  |  |  | LGALS3 |
| EGR1 |  |  |  | NEU1 |
| EHMT1 |  |  |  | LORICRIN |
| EIF2AK2 |  |  |  | CCL20 |
| EIF2S1 |  |  |  | IFNA2 |
| EIF4E |  |  |  | LRP1 |
| ELANE |  |  |  | PIK3C2A |
| ELN |  |  |  | THBD |
| ELOVL4 |  |  |  | TLR5 |
| ELR accum |  |  |  | MIR142 |
| EMSY |  |  |  | TNFRSF18 |
| ENKUR |  |  |  | RUNX1 |
| ENO1 |  |  |  | MMP3 |
| ENO2 |  |  |  | SFTPA2 |
| ENPP3 |  |  |  | EGF |
| ENTPD1 |  |  |  | WDR19 |
| EOE1 |  |  |  | MIF |
| EOE2 |  |  |  | ENPP3 |
| EP300 |  |  |  | NTF3 |
| EPHX1 |  |  |  | KLK7 |
| EPHX2 |  |  |  | KIF3A |
| EPRS1 |  |  |  | CCL27 |
| ERBB2 |  |  |  | FMR1 |
| ERBB4 |  |  |  | IL17D |
| ERCC1 |  |  |  | HRH4 |
| ERCC2 |  |  |  | TNFRSF1B |
| ERCC4 |  |  |  | PTX3 |
| ERCC6 |  |  |  | HMOX1 |
| ERMP1 |  |  |  | EGFR |
| ESR1 |  |  |  | IL6ST |
| ESRRA |  |  |  | DRD2 |
| ESS2 |  |  |  | RPS27A |
| EZR |  |  |  | PI3 |
| F2 |  |  |  | IDO1 |
| F2R |  |  |  | CD209 |
| F2RL1 |  |  |  | CHAT |
| F2RL3 |  |  |  | TGM3 |
| F3 |  |  |  | KLK5 |
| F8 |  |  |  | BBS10 |
| FADD |  |  |  | TMEM67 |
| FADS2 |  |  |  | USB1 |
| FAF2 |  |  |  | MT-ATP6 |
| FAM216A |  |  |  | GER |
| FAM53B |  |  |  | DEL18Q |
| FAM76B |  |  |  | ELN |
| FAM81B |  |  |  | NSD1 |
| FAR1 |  |  |  | NTS |
| FAS |  |  |  | HTR2A |
| FASLG |  |  |  | CSTA |
| FBLN5 |  |  |  | PRL |
| FBN1 |  |  |  | FCGR1A |
| FBN2 |  |  |  | IFIH1 |
| FCER1A |  |  |  | NTF4 |
| FCER1G |  |  |  | S100A9 |
| FCER2 |  |  |  | MIR146A |
| FCERG |  |  |  | ICAM3 |
| FCGR1A |  |  |  | CASP1 |
| FCGR3A |  |  |  | SLPI |
| FEN1 |  |  |  | MAVS |
| FGA |  |  |  | LY96 |
| FGF10 |  |  |  | HMGB1 |
| FGF14 |  |  |  | GBA |
| FGF7 |  |  |  | ATP12A |
| FGF8 |  |  |  | ATP4A |
| FGFBP2 |  |  |  | H2AC18 |
| FGFR1 |  |  |  | PEDS1 |
| FIP1L1 |  |  |  | MAPK1 |
| FKBP5 |  |  |  | IL10RA |
| FLAP |  |  |  | IRF3 |
| FLG |  |  |  | HSP90AA1 |
| FLG2 |  |  |  | HDC |
| FLG-AS1 |  |  |  | PAFAH1B1 |
| FLNC |  |  |  | SERPINB4 |
| FLT1 |  |  |  | ADRA1A |
| FLT3 |  |  |  | SERPINE1 |
| FMR1 |  |  |  | DEFB4A |
| FN1 |  |  |  | CHUK |
| FOXH1 |  |  |  | COL1A2 |
| FOXJ1 |  |  |  | EPHX1 |
| FOXP3 |  |  |  | DEFB103B |
| FPR1 |  |  |  | DDX58 |
| FRAXA |  |  |  | SUOX |
| FSIP1 |  |  |  | GNB3 |
| FURIN |  |  |  | CTNNA3 |
| G6PD |  |  |  | PSIP1 |
| GAA |  |  |  | S100A7 |
| GABBR1 |  |  |  | PTPN11 |
| GABRA2 |  |  |  | MBP |
| GABRB2 |  |  |  | STAT5B |
| GAD1 |  |  |  | HLA-A |
| GAD2 |  |  |  | BBS9 |
| GAL |  |  |  | FLG2 |
| GALNS |  |  |  | TGM1 |
| GALR1 |  |  |  | TMEM79 |
| GAPDH |  |  |  | TXK |
| GAS1 |  |  |  | MMP8 |
| GAS2L2 |  |  |  | TNXB |
| GAS8 |  |  |  | HRNR |
| GAST |  |  |  | IRF7 |
| GATA3 |  |  |  | CD83 |
| GATA4 |  |  |  | SMAD2 |
| GBA |  |  |  | IRF4 |
| GC |  |  |  | CD63 |
| GCG |  |  |  | MLX |
| GCH1 |  |  |  | ITGB3 |
| GER |  |  |  | BST2 |
| GFAP |  |  |  | EMSY |
| GGT1 |  |  |  | CYP2C19 |
| GHITM |  |  |  | TNFRSF1A |
| GHRH |  |  |  | ANXA5 |
| GHRL |  |  |  | NOS3 |
| GIVA cPLA2 |  |  |  | LBR |
| GJC2 |  |  |  | SLC6A11 |
| GLB1 |  |  |  | MIR21 |
| GLCCI1 |  |  |  | CCND1 |
| GLI2 |  |  |  | HERC5 |
| GLT6D1 |  |  |  | PSORS1C1 |
| GLTP |  |  |  | ADRA2B |
| GNAI1 |  |  |  | GAST |
| GNB3 |  |  |  | MX1 |
| GNPAT |  |  |  | KPNB1 |
| GNRH1 |  |  |  | LTF |
| GP1BA |  |  |  | MIR221 |
| GP1BB |  |  |  | WAS |
| GP6 |  |  |  | AQP3 |
| GP9 |  |  |  | ADSL |
| GPR12 |  |  |  | TPMT |
| GPT2 |  |  |  | KCNJ11 |
| GPX7 |  |  |  | DSG1 |
| GR |  |  |  | CTSE |
| GRHL2 |  |  |  | COTL1 |
| GRIN1 |  |  |  | CYP4F22 |
| GRK5 |  |  |  | SPN |
| GRP |  |  |  | LALBA |
| GS |  |  |  | FIP1L1 |
| GSDMA |  |  |  | CSN3 |
| GSDMB |  |  |  | DEFB103A |
| GSN |  |  |  | MYH11 |
| GSR |  |  |  | NTRK3 |
| GSTA1 |  |  |  | NAT2 |
| GSTM1 |  |  |  | TP53 |
| GSTO2 |  |  |  | SLC9A1 |
| GSTT1 |  |  |  | VWF |
| GTF2H4 |  |  |  | GCG |
| GTF2H5 |  |  |  | DEFB1 |
| GUCA2A |  |  |  | MICA |
| GUCY2C |  |  |  | RAD50 |
| GYS1 |  |  |  | TRAF3 |
| GZMB |  |  |  | KDM4C |
| H1R |  |  |  | PSMD3 |
| H2AC18 |  |  |  | FN1 |
| H3-2 |  |  |  | SFTPC |
| H4-16 |  |  |  | PRKCZ |
| H4C11 |  |  |  | SFTPB |
| H4C12 |  |  |  | HTR3A |
| H4C13 |  |  |  | FLT1 |
| H4C8 |  |  |  | RORA |
| H4R |  |  |  | PDGFRB |
| HAO1 |  |  |  | CD274 |
| HARS1 |  |  |  | GSDMA |
| HAVCR1 |  |  |  | MIR338 |
| HAVCR2 |  |  |  | CD36 |
| HBG2 |  |  |  | MMP21 |
| HCG23 |  |  |  | MYD88 |
| HCRT |  |  |  | TXN |
| HCRTR1 |  |  |  | KRT19 |
| HDAC1 |  |  |  | TGFA |
| HDAC2 |  |  |  | ERMP1 |
| HDAC8 |  |  |  | IFRD1 |
| HDAC9 |  |  |  | FURIN |
| HDC |  |  |  | NIPAL4 |
| HERC5 |  |  |  | FAM76B |
| HIF1A |  |  |  | SULT1A3 |
| HIRA |  |  |  | VDAC1 |
| His rele |  |  |  | ESS2 |
| His/p-LT rele |  |  |  | BCL2 |
| HLA-A |  |  |  | TFAP2A |
| HLA-B |  |  |  | IFNGR1 |
| HLA-C |  |  |  | LEPQTL1 |
| HLA-DMA |  |  |  | F8 |
| HLA-DMB |  |  |  | APC |
| HLA-DOA |  |  |  | CAV1 |
| HLA-DOB |  |  |  | TGFB3 |
| HLA-DPA1 |  |  |  | STX1A |
| HLA-DPB1 |  |  |  | ALAD |
| HLA-DQA1 |  |  |  | PRODH |
| HLA-DQA2 |  |  |  | CANX |
| HLA-DQB1 |  |  |  | FLNC |
| HLA-DRA |  |  |  | CD2 |
| HLA-DRB1 |  |  |  | SLC25A6 |
| HLA-DRB5 |  |  |  | TREX1 |
| HLA-G |  |  |  | CABIN1 |
| HM13 |  |  |  | SUZ12 |
| HMGB1 |  |  |  | TGM5 |
| HMGCR |  |  |  | CLIC2 |
| HMOX1 |  |  |  | DGCR8 |
| HNMT |  |  |  | LZTFL1 |
| HP |  |  |  | DGCR2 |
| HPGDS |  |  |  | RAB39B |
| HR |  |  |  | KIF7 |
| HRAS |  |  |  | IVNS1ABP |
| HRH1 |  |  |  | DCTN4 |
| HRH2 |  |  |  | WAC |
| HRH4 |  |  |  | CLCA4 |
| HRNR |  |  |  | TTC12 |
| HSD11B1 |  |  |  | C19orf12 |
| HSD11B2 |  |  |  | MPLKIP |
| HSP20 |  |  |  | HARS1 |
| HSP90 |  |  |  | NEK10 |
| HSP90AA1 |  |  |  | DNAH7 |
| HSPA1A |  |  |  | VPS51 |
| HSPA1B |  |  |  | BBIP1 |
| HSPA8 |  |  |  | DGCR6 |
| HSPG2 |  |  |  | NM |
| HTR2A |  |  |  | EOE1 |
| HTR3A |  |  |  | EOE2 |
| HTRA2 |  |  |  | CILD4 |
| HYDIN |  |  |  | CILD8 |
| IAPP |  |  |  | DUPXQ28 |
| ICAM3 |  |  |  | DEL16P13.2 |
| ICOS |  |  |  | DEL16P13.3 |
| ICOSLG |  |  |  | PAND1 |
| IDH1 |  |  |  | PAND2 |
| IDO1 |  |  |  | PAND3 |
| IDS |  |  |  | LYN |
| IFI27 |  |  |  | CTRL |
| IFIH1 |  |  |  | TAC3 |
| IFITM1 |  |  |  | RNASEH2C |
| IFITM2 |  |  |  | BBS12 |
| IFITM3 |  |  |  | MIR199A1 |
| IFNA1 |  |  |  | TERT |
| IFNA2 |  |  |  | SOCS2 |
| IFNB1 |  |  |  | S100A12 |
| IFNGR1 |  |  |  | MIR145 |
| IFNL1 |  |  |  | TNFSF4 |
| IFRD1 |  |  |  | SERPINB1 |
| IFT122 |  |  |  | MIR9-1 |
| IFT140 |  |  |  | TAP2 |
| IFT80 |  |  |  | BPI |
| IFT81 |  |  |  | MIR200C |
| IFT88 |  |  |  | MIR200B |
| IgE |  |  |  | ATG3 |
| IGF1 |  |  |  | KRT1 |
| IGFBP3 |  |  |  | PRKG1 |
| IK |  |  |  | RAC2 |
| IKZF3 |  |  |  | KCNQ1 |
| IKZF4 |  |  |  | AGER |
| IL-1 synth/rele |  |  |  | PDGFB |
| IL10 |  |  |  | HLA-C |
| IL10RA |  |  |  | TTR |
| IL11 |  |  |  | NPPB |
| IL12A |  |  |  | GRP |
| IL12B |  |  |  | CCR10 |
| IL12RB1 |  |  |  | EIF2AK2 |
| IL12RB2 |  |  |  | MAOA |
| IL13 |  |  |  | CALR |
| IL13RA1 |  |  |  | SMPD1 |
| IL13RA2 |  |  |  | PSMB8 |
| IL15 |  |  |  | MIRLET7A1 |
| IL16 |  |  |  | CD27 |
| IL17 |  |  |  | TMEM132D |
| IL17D |  |  |  | IL12A |
| IL17F |  |  |  | ATF6 |
| IL17R |  |  |  | ASOBS |
| IL17RB |  |  |  | CRHR2 |
| IL18BP |  |  |  | IL25 |
| IL18R1 |  |  |  | SIGLEC8 |
| IL18RAP |  |  |  | SLC6A2 |
| IL1A |  |  |  | GCH1 |
| IL1R1 |  |  |  | SLC18A2 |
| IL1RL1 |  |  |  | DRD4 |
| IL1RN |  |  |  | GP6 |
| IL21 |  |  |  | PTK7 |
| IL21R |  |  |  | CHGA |
| IL22 |  |  |  | STS |
| IL23 |  |  |  | PYY |
| IL23A |  |  |  | BBS2 |
| IL25 |  |  |  | BBS4 |
| IL2RB |  |  |  | CCK |
| IL2RG |  |  |  | PTPN22 |
| IL31 |  |  |  | PHB |
| IL31RA |  |  |  | COL2A1 |
| IL33 |  |  |  | KRT18 |
| IL37 |  |  |  | MUC1 |
| IL3RA |  |  |  | DRD3 |
| IL4 |  |  |  | SLC2A10 |
| IL4R |  |  |  | DNASE1 |
| IL5 |  |  |  | BBS7 |
| IL5RA |  |  |  | TMEM216 |
| IL6R |  |  |  | MLN |
| IL6ST |  |  |  | TAP1 |
| IL7 |  |  |  | BPIFA1 |
| IL7R |  |  |  | ISG15 |
| IL9 |  |  |  | ALDH3A2 |
| IL9R |  |  |  | MECP2 |
| INHBA |  |  |  | PML |
| INS |  |  |  | ST14 |
| INSR |  |  |  | PHYH |
| IRAK3 |  |  |  | RANBP2 |
| IRAK4 |  |  |  | XPO1 |
| IRF1 |  |  |  | CASP14 |
| IRF1-AS1 |  |  |  | EIF2S1 |
| IRF3 |  |  |  | PABPN1 |
| IRF4 |  |  |  | SLC26A2 |
| IRF5 |  |  |  | TMPRSS2 |
| IRF7 |  |  |  | TRIM25 |
| ISG15 |  |  |  | ALOX12B |
| IsoM |  |  |  | NPHP1 |
| ITGA2 |  |  |  | KPNA3 |
| ITGA2B |  |  |  | ABCA12 |
| ITGA3 |  |  |  | NUP98 |
| ITGA4 |  |  |  | KLK6 |
| ITGA4/B1 |  |  |  | KPNA1 |
| ITGA5 |  |  |  | KLK4 |
| ITGAL |  |  |  | KRT10 |
| ITGAM |  |  |  | ALOXE3 |
| ITGB1 |  |  |  | IFITM1 |
| ITGB2 |  |  |  | U2AF1 |
| ITGB3 |  |  |  | CPSF4 |
| ITK |  |  |  | DNAJC3 |
| ITLN1 |  |  |  | PDCL |
| IVL |  |  |  | RSAD2 |
| IVNS1ABP |  |  |  | NLRX1 |
| JAK-1 |  |  |  | NXF1 |
| JAK2 |  |  |  | TSHZ1 |
| JAK-2 |  |  |  | CALCOCO2 |
| JAK-3 |  |  |  | KPNA5 |
| JMJD1C |  |  |  | MX2 |
| JUN |  |  |  | IK |
| KAT2A |  |  |  | DSC1 |
| KAT2B |  |  |  | CHD6 |
| KAT6B |  |  |  | IFITM2 |
| KC |  |  |  | PTER |
| KCNJ11 |  |  |  | KLK14 |
| KCNMA1 |  |  |  | STAU1 |
| KCNQ1 |  |  |  | PACRG |
| KDM1A |  |  |  | MORC3 |
| KDM4C |  |  |  | DCD |
| KDM6A |  |  |  | PAF1 |
| KIAA1109 |  |  |  | SMU1 |
| KIF3A |  |  |  | TTLL1 |
| KIF7 |  |  |  | RRP1B |
| KIT |  |  |  | TTC38 |
| KITLG |  |  |  | FAM81B |
| KLK14 |  |  |  | DGCR6L |
| KLK3 |  |  |  | OR4D10 |
| KLK4 |  |  |  | FAM216A |
| KLK5 |  |  |  | SPINK9 |
| KLK6 |  |  |  | RTRAF |
| KLK7 |  |  |  | WDR87 |
| KLRG1 |  |  |  | OR8K3 |
| KNG1 |  |  |  | CLCN3 |
| KPNA1 |  |  |  | PCNA |
| KPNA3 |  |  |  | MUC4 |
| KPNA5 |  |  |  | RANBP6 |
| KPNB1 |  |  |  | SPTBN2 |
| KRT1 |  |  |  | CYFIP2 |
| KRT10 |  |  |  | CYBB |
| KRT13 |  |  |  | NETO1 |
| KRT14 |  |  |  | WDR36 |
| KRT18 |  |  |  | BCL6 |
| KRT19 |  |  |  | GTF2H4 |
| KRT24 |  |  |  | OXA1L |
| LALBA |  |  |  | GC |
| LAT |  |  |  | SRC |
| LBR |  |  |  | MIR16-1 |
| L-CaC |  |  |  | PSMB9 |
| LCN2 |  |  |  | CEP89 |
| LEP |  |  |  | SLC25A46 |
| LEPQTL1 |  |  |  | MIR20B |
| LEPR |  |  |  | RGS2 |
| LGALS3 |  |  |  | SERPINE2 |
| LIFR |  |  |  | SMAD6 |
| LIG1 |  |  |  | MIR15A |
| LIG4 |  |  |  | C3 |
| LILRB3 |  |  |  | ORMDL2 |
| LMX1B |  |  |  | MYH9 |
| LNX1 |  |  |  | IKZF3 |
| LORICRIN |  |  |  | KRT13 |
| LPO |  |  |  | MIR192 |
| LRBA |  |  |  | RAC1 |
| LRP1 |  |  |  | COL6A5 |
| LRRC32 |  |  |  | SMAD4 |
| LRRC46 |  |  |  | NFKB1 |
| LRRC56 |  |  |  | MIR223 |
| LRRC6 |  |  |  | PLAUR |
| LRRK2 |  |  |  | MUC6 |
| LT synth |  |  |  | SLC11A1 |
| LTA |  |  |  | ROM1 |
| LTA4H |  |  |  | XRCC1 |
| LTB |  |  |  | FBN2 |
| LTB4R |  |  |  | RECK |
| LTB4R2 |  |  |  | PLA2R1 |
| LTC4S |  |  |  | RTEL1 |
| LTF |  |  |  | MEN1 |
| LTR |  |  |  | EHMT1 |
| LY96 |  |  |  | MPZ |
| LYN |  |  |  | EFEMP2 |
| LZTFL1 |  |  |  | CATSPER1 |
| M1 IgE |  |  |  | ATAT1 |
| MAOA |  |  |  | MIR28 |
| MAP3K9 |  |  |  | EZR |
| MAPK1 |  |  |  | P2RY2 |
| MAPK14 |  |  |  | MIR29C |
| MAPK3 |  |  |  | ATG5 |
| MAPK8 |  |  |  | CCR2 |
| MARCKS |  |  |  | CHRM2 |
| MARCO |  |  |  | AOAH |
| MAVS |  |  |  | ANXA1 |
| MB |  |  |  | NOTCH1 |
| MBP |  |  |  | APOH |
| MC4R |  |  |  | PTPRC |
| MCIDAS |  |  |  | ADCY3 |
| MCL1 |  |  |  | CHD7 |
| MECP2 |  |  |  | PPL |
| MED24 |  |  |  | CNTLN |
| MEI1 |  |  |  | MIR222 |
| MEN1 |  |  |  | SCP2 |
| MFAP5 |  |  |  | MSR1 |
| MICA |  |  |  | TNNT1 |
| MICB |  |  |  | BMP4 |
| MIF |  |  |  | MIR23A |
| MIR10A |  |  |  | MYC |
| MIR124-1 |  |  |  | CASR |
| MIR126 |  |  |  | NTRK1 |
| MIR132 |  |  |  | CDH26 |
| MIR140 |  |  |  | MIR149 |
| MIR142 |  |  |  | TRPA1 |
| MIR143 |  |  |  | SLC22A5 |
| MIR145 |  |  |  | HLA-DMA |
| MIR146A |  |  |  | ERBB2 |
| MIR149 |  |  |  | LRRC32 |
| MIR15A |  |  |  | SCNN1A |
| MIR15B |  |  |  | GABBR1 |
| MIR16-1 |  |  |  | ENO2 |
| MIR17 |  |  |  | NDFIP1 |
| MIR181A1 |  |  |  | ZPBP2 |
| MIR183 |  |  |  | SPP1 |
| MIR192 |  |  |  | C4A |
| MIR196A1 |  |  |  | SNCA |
| MIR196A2 |  |  |  | ACTA2 |
| MIR199A1 |  |  |  | AGPS |
| MIR200A |  |  |  | NPTN |
| MIR200B |  |  |  | MIR132 |
| MIR200C |  |  |  | MIR10A |
| MIR20A |  |  |  | CYLD |
| MIR20B |  |  |  | FEN1 |
| MIR21 |  |  |  | ERCC1 |
| MIR210 |  |  |  | NAGLU |
| MIR22 |  |  |  | ATXN2 |
| MIR221 |  |  |  | AHI1 |
| MIR222 |  |  |  | RCOR1 |
| MIR223 |  |  |  | MEI1 |
| MIR23A |  |  |  | KRT24 |
| MIR24-1 |  |  |  | RPAP3 |
| MIR28 |  |  |  | MIR34C |
| MIR29A |  |  |  | MTHFR |
| MIR29C |  |  |  | TPSB2 |
| MIR30E |  |  |  | SERPINB2 |
| MIR31 |  |  |  | TBXT |
| MIR338 |  |  |  | HLA-DMB |
| MIR34C |  |  |  | HLA-DOB |
| MIR423 |  |  |  | HLA-DRB5 |
| MIR483 |  |  |  | PBX2 |
| MIR486-1 |  |  |  | MUC2 |
| MIR532 |  |  |  | HSPA1A |
| MIR574 |  |  |  | IL21 |
| MIR9-1 |  |  |  | ANGPT1 |
| MIRLET7A1 |  |  |  | TNFAIP3 |
| MLN |  |  |  | CYP26A1 |
| MLX |  |  |  | FLT3 |
| MME |  |  |  | NRAS |
| MMP1 |  |  |  | DGKE |
| MMP12 |  |  |  | GAA |
| MMP-12 |  |  |  | KDM1A |
| MMP19 |  |  |  | GPT2 |
| MMP2 |  |  |  | GNPAT |
| MMP21 |  |  |  | ATP7A |
| MMP28 |  |  |  | ALDH18A1 |
| MMP3 |  |  |  | ADRA2C |
| MMP8 |  |  |  | ERCC6 |
| MMP9 |  |  |  | GUCY2C |
| MMRN1 |  |  |  | GALR1 |
| MOP |  |  |  | FGF14 |
| MORC3 |  |  |  | DCLRE1C |
| MPIP1 |  |  |  | HAO1 |
| MPLKIP |  |  |  | AVPR1B |
| MPO |  |  |  | FAR1 |
| MPZ |  |  |  | CDX2 |
| MRGPRX2 |  |  |  | GPX7 |
| MS4A2 |  |  |  | PEX7 |
| MSR1 |  |  |  | DNASE1L3 |
| MT-ATP6 |  |  |  | RNASEH2A |
| MTHFR |  |  |  | CETN2 |
| MTOR |  |  |  | PGC |
| MUC1 |  |  |  | SNAP29 |
| MUC2 |  |  |  | GJC2 |
| MUC22 |  |  |  | ASCC1 |
| MUC4 |  |  |  | MFAP5 |
| MUC5AC |  |  |  | CTHRC1 |
| MUC5B |  |  |  | PSMD12 |
| MUC6 |  |  |  | RECQL4 |
| MUC7 |  |  |  | GHRH |
| MX1 |  |  |  | CEP290 |
| MX2 |  |  |  | IFT88 |
| MYB |  |  |  | BBS5 |
| MYC |  |  |  | RPGRIP1L |
| MycB mmpL3 |  |  |  | NPHP3 |
| MYD88 |  |  |  | IFT81 |
| MYH11 |  |  |  | SMTN |
| MYH9 |  |  |  | TOR1B |
| nAChR |  |  |  | GHITM |
| NAGLU |  |  |  | DYNLT1 |
| NAT2 |  |  |  | IFT80 |
| NDFIP1 |  |  |  | KLRG1 |
| NEAT1 |  |  |  | RNASEH2B |
| nedA |  |  |  | NELFA |
| NEK10 |  |  |  | SALL3 |
| NEK9 |  |  |  | SLAMF8 |
| NELFA |  |  |  | PEX5L |
| NETO1 |  |  |  | SRY |
| NEU1 |  |  |  | FAM53B |
| NFE2L2 |  |  |  | ZNF516 |
| NFKB |  |  |  | ZNF469 |
| NFKB1 |  |  |  | SDAD1 |
| NFKB2 |  |  |  | NUTM1 |
| NFKBIA |  |  |  | TULP4 |
| NFKBIL1 |  |  |  | TTC28 |
| NGF |  |  |  | ZNF236 |
| NIPAL4 |  |  |  | DRC7 |
| NKX2-1 |  |  |  | CEP295 |
| NLRP3 |  |  |  | MIR200A |
| NLRX1 |  |  |  | MIR210 |
| NM |  |  |  | MIR30E |
| NME8 |  |  |  | MIR17 |
| NOD1 |  |  |  | MIR31 |
| NODAL |  |  |  | MIR196A1 |
| NOMO1 |  |  |  | MIR15B |
| NOS1 |  |  |  | MIR423 |
| NOS2 |  |  |  | MIR24-1 |
| NOS3 |  |  |  | H3-2 |
| NOTCH1 |  |  |  | MIR532 |
| NOTCH4 |  |  |  | FRAXA |
| NPHP1 |  |  |  | TOP2A |
| NPHP3 |  |  |  | CYP21A2 |
| NPPA |  |  |  | GSTA1 |
| NPPB |  |  |  | GGT1 |
| NPS |  |  |  | DPP4 |
| NPSR1 |  |  |  | TLE4 |
| NPSR1-AS1 |  |  |  | TPSD1 |
| NPSRAS1 |  |  |  | ALDH2 |
| NPTN |  |  |  | PRKAA2 |
| NPY |  |  |  | POU2F1 |
| NQO1 |  |  |  | IL7R |
| NR0B1 |  |  |  | LCN2 |
| NR1I2 |  |  |  | IDH1 |
| NR3C1 |  |  |  | SMARCA2 |
| NR3C2 |  |  |  | GFAP |
| NRAS |  |  |  | ALDH1A2 |
| NRXN1 |  |  |  | PAX3 |
| NSD1 |  |  |  | NRXN1 |
| NSUN2 |  |  |  | KAT2A |
| NTF3 |  |  |  | LIG1 |
| NTF4 |  |  |  | PSMA6 |
| NTRK1 |  |  |  | ERCC4 |
| NTRK3 |  |  |  | CLCN2 |
| NTS |  |  |  | GALNS |
| NUP98 |  |  |  | HCRTR1 |
| NUTM1 |  |  |  | LNX1 |
| NXF1 |  |  |  | SLC6A13 |
| OFD1 |  |  |  | PSMD2 |
| OPR |  |  |  | XRCC4 |
| OPRM1 |  |  |  | RNF5 |
| OR4D10 |  |  |  | IFT122 |
| OR8K3 |  |  |  | RAB8A |
| ORM1 |  |  |  | CDC5L |
| ORMDL2 |  |  |  | FAF2 |
| ORMDL3 |  |  |  | DYNC2H1 |
| OX40 |  |  |  | CFHR2 |
| OXA1L |  |  |  | CHIC2 |
| P2RX7 |  |  |  | SAMD12 |
| P2RY12 |  |  |  | WDPCP |
| P2RY2 |  |  |  | CEP83 |
| p38 |  |  |  | SDK2 |
| PABPN1 |  |  |  | ENKUR |
| PACRG |  |  |  | TNP1 |
| PAF1 |  |  |  | LRRC46 |
| PAFAH1B1 |  |  |  | H4C8 |
| PAND1 |  |  |  | RSPH6A |
| PAND2 |  |  |  | H4C11 |
| PAND3 |  |  |  | DAW1 |
| PARP1 |  |  |  | H4C12 |
| PAX3 |  |  |  | H4C13 |
| PBX2 |  |  |  | CFAP54 |
| PCDH1 |  |  |  | NEAT1 |
| PCNA |  |  |  | MIR574 |
| PDCD1 |  |  |  | MIR181A1 |
| PDCD5 |  |  |  | SLC7A2 |
| PDCL |  |  |  | ICOS |
| PDE |  |  |  | UBC |
| PDE3 |  |  |  | AICDA |
| PDE3A |  |  |  | DUSP1 |
| PDE4 |  |  |  | CDH1 |
| PDE4A |  |  |  | CACNG6 |
| PDE4B |  |  |  | MIR140 |
| PDE4D |  |  |  | ERBB4 |
| PDE5A |  |  |  | IL22 |
| PDGFB |  |  |  | IL23A |
| PDGFRA |  |  |  | TPSG1 |
| PDGFRB |  |  |  | SLC22A4 |
| PEDS1 |  |  |  | IGF1 |
| PEPD |  |  |  | CASP10 |
| PEX5 |  |  |  | CFLAR |
| PEX5L |  |  |  | MRGPRX2 |
| PEX7 |  |  |  | MYB |
| PF4 |  |  |  | CLEC16A |
| PGAP3 |  |  |  | RBM17 |
| PGC |  |  |  | PNMT |
| PGF |  |  |  | FADD |
| PGM3 |  |  |  | IRF1-AS1 |
| PGR |  |  |  | TSPO |
| PHB |  |  |  | SOD3 |
| PHF11 |  |  |  | COX5A |
| PHYH |  |  |  | CTCF |
| PI3 |  |  |  | PRKCQ |
| PIK3C2A |  |  |  | HSPG2 |
| PIK3CD |  |  |  | TPH1 |
| PIK3CG |  |  |  | SLC26A9 |
| PITX2 |  |  |  | MIR143 |
| PK |  |  |  | ESR1 |
| PLA2G10 |  |  |  | HSPA8 |
| PLA2G1B |  |  |  | KIAA1109 |
| PLA2G2A |  |  |  | AQP5 |
| PLA2G4A |  |  |  | DDX39B |
| PLA2G7 |  |  |  | NFKBIL1 |
| PLA2R1 |  |  |  | IL2RG |
| PLAU |  |  |  | IRAK4 |
| PLAUR |  |  |  | FBLN5 |
| PLCG2 |  |  |  | IFNL1 |
| PLG |  |  |  | PIK3CD |
| PMCH |  |  |  | HIF1A |
| PMEL |  |  |  | MUC22 |
| PML |  |  |  | RUNX3 |
| PNMT |  |  |  | RETN |
| POLR1H |  |  |  | TSC2 |
| POMC |  |  |  | GSN |
| PON1 |  |  |  | TSC1 |
| POSTN |  |  |  | HDAC8 |
| POU2F1 |  |  |  | SRSF7 |
| PPARG |  |  |  | PMCH |
| PPBP |  |  |  | CRB1 |
| PPL |  |  |  | NOTCH4 |
| PRG2 |  |  |  | BRD2 |
| PRKAA2 |  |  |  | MICB |
| PRKCA |  |  |  | ATF6B |
| PRKCQ |  |  |  | HCG23 |
| PRKCZ |  |  |  | MED24 |
| PRKG1 |  |  |  | ZBTB10 |
| PRL |  |  |  | ITGB1 |
| PRODH |  |  |  | IRF5 |
| PRRC2A |  |  |  | CD34 |
| PRSS1 |  |  |  | CLCN5 |
| PRTN3 |  |  |  | IL3RA |
| PSIP1 |  |  |  | CXCL11 |
| PSMA6 |  |  |  | PSMC4 |
| PSMB8 |  |  |  | IFT140 |
| PSMB9 |  |  |  | GNAI1 |
| PSMC4 |  |  |  | SPATS2L |
| PSMC6 |  |  |  | PCDH1 |
| PSMD12 |  |  |  | IL17RB |
| PSMD2 |  |  |  | ARSB |
| PSMD3 |  |  |  | PTPRD |
| PSORS1C1 |  |  |  | IL37 |
| PTAFR |  |  |  | CCHCR1 |
| PTCH1 |  |  |  | SLC26A4 |
| PTEN |  |  |  | CD177 |
| PTER |  |  |  | PLA2G10 |
| PTGDR |  |  |  | STAT4 |
| PTGDR2 |  |  |  | PON1 |
| PTGER2 |  |  |  | RORC |
| PTGER3 |  |  |  | AKR1C3 |
| PTGER4 |  |  |  | LEPR |
| PTGIR |  |  |  | CYBA |
| PTGS1 |  |  |  | C9orf24 |
| PTGS2 |  |  |  | CD247 |
| PTK |  |  |  | SMARCE1 |
| PTK7 |  |  |  | AOC1 |
| PTPN11 |  |  |  | CTSS |
| PTPN22 |  |  |  | EDIL3 |
| PTPRC |  |  |  | BTNL2 |
| PTPRD |  |  |  | H4-16 |
| PTX3 |  |  |  | DDIT3 |
| PVT1 |  |  |  | LTB |
| PYY |  |  |  | AIF1 |
| RAB39B |  |  |  | PRRC2A |
| RAB8A |  |  |  | ANXA2 |
| RAC1 |  |  |  | CEBPB |
| RAC2 |  |  |  | CYP2R1 |
| RAD50 |  |  |  | GSTO2 |
| RANBP2 |  |  |  | IL18RAP |
| RANBP6 |  |  |  | SERPINB10 |
| RAPGEF3 |  |  |  | PRKCA |
| RASGRP4 |  |  |  | C5 |
| RBM17 |  |  |  | MMP19 |
| RCOR1 |  |  |  | FADS2 |
| RECK |  |  |  | TIMELESS |
| RECQL4 |  |  |  | CPS1 |
| REN |  |  |  | MIR196A2 |
| RETN |  |  |  | MARCO |
| RGS2 |  |  |  | INSR |
| RHO |  |  |  | GYS1 |
| RIC1 |  |  |  | RHO |
| RIC3 |  |  |  | ADRA1D |
| RIPK1 |  |  |  | HCRT |
| RLN1 |  |  |  | IAPP |
| RNASE2 |  |  |  | AGT |
| RNASE3 |  |  |  | SLC24A2 |
| RNASEH2A |  |  |  | CDK2 |
| RNASEH2B |  |  |  | HSPA1B |
| RNASEH2C |  |  |  | PDCD5 |
| RNF5 |  |  |  | PMEL |
| ROCK |  |  |  | IKZF4 |
| ROM1 |  |  |  | TSBP1 |
| RORA |  |  |  | HPGDS |
| RORC |  |  |  | CDKN3 |
| ROS |  |  |  | ARRB2 |
| RPAP3 |  |  |  | IL18BP |
| RPGR |  |  |  | APOA1 |
| RPGRIP1L |  |  |  | CXCR5 |
| RPS27A |  |  |  | MMP28 |
| RREB1 |  |  |  | GPR12 |
| RRP1B |  |  |  | ITLN1 |
| RSAD2 |  |  |  | APEX1 |
| RSPH1 |  |  |  | CD9 |
| RSPH3 |  |  |  | PSMC6 |
| RSPH4A |  |  |  | MAPK8 |
| RSPH6A |  |  |  | CYP1A1 |
| RSPH9 |  |  |  | REN |
| RTEL1 |  |  |  | CHRM1 |
| RTRAF |  |  |  | TIMD4 |
| RUNX1 |  |  |  | P2RX7 |
| RUNX3 |  |  |  | PARP1 |
| RYR2 |  |  |  | WT1 |
| S100A12 |  |  |  | GATA4 |
| S100A7 |  |  |  | ABCA3 |
| S100A8 |  |  |  | FGF10 |
| S100A9 |  |  |  | ENTPD1 |
| S100P |  |  |  | TKT |
| SALL3 |  |  |  | SLC9A3R1 |
| SAMD12 |  |  |  | FGF7 |
| SART1 |  |  |  | GUCA2A |
| SCGB1A1 |  |  |  | IFI27 |
| SCGB3A2 |  |  |  | MIR20A |
| SCN10A |  |  |  | ENO1 |
| SCN4A |  |  |  | SEMA3E |
| SCN9A |  |  |  | INHBA |
| SCNN1A |  |  |  | PGAP3 |
| SCP2 |  |  |  | HDAC9 |
| SDAD1 |  |  |  | BAX |
| SDHB |  |  |  | CTNNB1 |
| SDHD |  |  |  | EPHX2 |
| SDK2 |  |  |  | HDAC1 |
| SEC24C |  |  |  | HRAS |
| SEL |  |  |  | GAD1 |
| SELE |  |  |  | ITGA2B |
| SELL |  |  |  | LRRK2 |
| SELP |  |  |  | RIPK1 |
| SELPLG |  |  |  | PLG |
| SEMA3E |  |  |  | TNNI3 |
| SERPINA1 |  |  |  | KAT2B |
| SERPINA3 |  |  |  | HTRA2 |
| SERPINA6 |  |  |  | GAD2 |
| SERPINB1 |  |  |  | TXNRD2 |
| SERPINB10 |  |  |  | KDM6A |
| SERPINB2 |  |  |  | ITGA3 |
| SERPINB3 |  |  |  | CNTN2 |
| SERPINB4 |  |  |  | TBX5 |
| SERPINE1 |  |  |  | CCKBR |
| SERPINE2 |  |  |  | BSG |
| SETDB2 |  |  |  | BTD |
| SFTPA1 |  |  |  | DAB2 |
| SFTPA2 |  |  |  | SI |
| SFTPB |  |  |  | PGF |
| SFTPC |  |  |  | IL31RA |
| SFTPD |  |  |  | BHLHE40 |
| SHBG |  |  |  | CEACAM5 |
| SHH |  |  |  | KAT6B |
| SI |  |  |  | DPEP2 |
| SIGLEC5 |  |  |  | DCDC2 |
| SIGLEC8 |  |  |  | LILRB3 |
| SIK3 |  |  |  | SIGLEC5 |
| SIX3 |  |  |  | RLN1 |
| SLAMF8 |  |  |  | MIR124-1 |
| SLC11A1 |  |  |  | MIR29A |
| SLC13A2 |  |  |  | MIR183 |
| SLC13A5 |  |  |  | MIR483 |
| SLC18A2 |  |  |  | MIR486-1 |
| SLC22A1 |  |  |  | CREB5 |
| SLC22A2 |  |  |  | AKT1 |
| SLC22A3 |  |  |  | HMGCR |
| SLC22A4 |  |  |  |  |
| SLC22A5 |  |  |  |  |
| SLC22A7 |  |  |  |  |
| SLC22A8 |  |  |  |  |
| SLC24A2 |  |  |  |  |
| SLC25A1 |  |  |  |  |
| SLC25A21 |  |  |  |  |
| SLC25A46 |  |  |  |  |
| SLC25A6 |  |  |  |  |
| SLC26A2 |  |  |  |  |
| SLC26A4 |  |  |  |  |
| SLC26A9 |  |  |  |  |
| SLC2A10 |  |  |  |  |
| SLC6A11 |  |  |  |  |
| SLC6A13 |  |  |  |  |
| SLC6A2 |  |  |  |  |
| SLC6A3 |  |  |  |  |
| SLC6A4 |  |  |  |  |
| SLC7A2 |  |  |  |  |
| SLC9A1 |  |  |  |  |
| SLC9A3R1 |  |  |  |  |
| SLCO1A2 |  |  |  |  |
| SLCO1B1 |  |  |  |  |
| SLCO1B3 |  |  |  |  |
| SLCO2B1 |  |  |  |  |
| SLPI |  |  |  |  |
| SMAD2 |  |  |  |  |
| SMAD3 |  |  |  |  |
| SMAD4 |  |  |  |  |
| SMAD6 |  |  |  |  |
| SMARCA2 |  |  |  |  |
| SMARCE1 |  |  |  |  |
| SMPD1 |  |  |  |  |
| SMTN |  |  |  |  |
| SMU1 |  |  |  |  |
| SNAP29 |  |  |  |  |
| SNCA |  |  |  |  |
| SOCS1 |  |  |  |  |
| SOCS2 |  |  |  |  |
| SOCS3 |  |  |  |  |
| SOD1 |  |  |  |  |
| SOD2 |  |  |  |  |
| SOD3 |  |  |  |  |
| SP1 |  |  |  |  |
| SPAG1 |  |  |  |  |
| SPATS2L |  |  |  |  |
| SPEF2 |  |  |  |  |
| SPINK5 |  |  |  |  |
| SPINK9 |  |  |  |  |
| SPN |  |  |  |  |
| SPP1 |  |  |  |  |
| SPRED1 |  |  |  |  |
| SPTBN2 |  |  |  |  |
| SRC |  |  |  |  |
| SRD5A2 |  |  |  |  |
| SRSF2 |  |  |  |  |
| SRSF7 |  |  |  |  |
| SRY |  |  |  |  |
| SST |  |  |  |  |
| ST14 |  |  |  |  |
| STAT3 |  |  |  |  |
| STAT4 |  |  |  |  |
| STAT5A |  |  |  |  |
| STAT5B |  |  |  |  |
| STAT6 |  |  |  |  |
| STAU1 |  |  |  |  |
| STK36 |  |  |  |  |
| STS |  |  |  |  |
| STX1A |  |  |  |  |
| STXBP1 |  |  |  |  |
| SUFU |  |  |  |  |
| SULT1A3 |  |  |  |  |
| SULT2A1 |  |  |  |  |
| SUOX |  |  |  |  |
| SUZ12 |  |  |  |  |
| SYK |  |  |  |  |
| SYK mRNA |  |  |  |  |
| TAC1 |  |  |  |  |
| TAC3 |  |  |  |  |
| TACR1 |  |  |  |  |
| TACR2 |  |  |  |  |
| TACR3 |  |  |  |  |
| TALDO1 |  |  |  |  |
| TAP1 |  |  |  |  |
| TAP2 |  |  |  |  |
| TBCK |  |  |  |  |
| TBX1 |  |  |  |  |
| TBX21 |  |  |  |  |
| TBX5 |  |  |  |  |
| TBXA2R |  |  |  |  |
| TBXAS1 |  |  |  |  |
| TBXT |  |  |  |  |
| TDGF1 |  |  |  |  |
| TERT |  |  |  |  |
| TET2 |  |  |  |  |
| TF |  |  |  |  |
| TFAP2A |  |  |  |  |
| TGFA |  |  |  |  |
| TGFB2 |  |  |  |  |
| TGFB3 |  |  |  |  |
| TGFBR1 |  |  |  |  |
| TGFBR2 |  |  |  |  |
| TGIF1 |  |  |  |  |
| TGM1 |  |  |  |  |
| TGM3 |  |  |  |  |
| TGM5 |  |  |  |  |
| THBD |  |  |  |  |
| THPO |  |  |  |  |
| TIMD4 |  |  |  |  |
| TIMELESS |  |  |  |  |
| TIMP1 |  |  |  |  |
| TKT |  |  |  |  |
| TLE4 |  |  |  |  |
| TLR1 |  |  |  |  |
| TLR10 |  |  |  |  |
| TLR3 |  |  |  |  |
| TLR4 |  |  |  |  |
| TLR5 |  |  |  |  |
| TLR6 |  |  |  |  |
| TLR7 |  |  |  |  |
| TLR9 |  |  |  |  |
| TMEM132D |  |  |  |  |
| TMEM216 |  |  |  |  |
| TMEM67 |  |  |  |  |
| TMEM79 |  |  |  |  |
| TMPRSS2 |  |  |  |  |
| TNF |  |  |  |  |
| TNFAIP3 |  |  |  |  |
| TNFR |  |  |  |  |
| TNFRSF10A |  |  |  |  |
| TNFRSF18 |  |  |  |  |
| TNFRSF1A |  |  |  |  |
| TNFRSF1B |  |  |  |  |
| TNFRSF4 |  |  |  |  |
| TNFRSF8 |  |  |  |  |
| TNFSF10 |  |  |  |  |
| TNFSF13B |  |  |  |  |
| TNFSF14 |  |  |  |  |
| TNFSF4 |  |  |  |  |
| TNIP1 |  |  |  |  |
| TNNI3 |  |  |  |  |
| TNNT1 |  |  |  |  |
| TNP1 |  |  |  |  |
| TNXB |  |  |  |  |
| TOP2 |  |  |  |  |
| TOP2A |  |  |  |  |
| TOR1B |  |  |  |  |
| TP53 |  |  |  |  |
| TPH1 |  |  |  |  |
| TPMT |  |  |  |  |
| TPSAB1 |  |  |  |  |
| TPSB2 |  |  |  |  |
| TPSD1 |  |  |  |  |
| TPSG1 |  |  |  |  |
| TPT1 |  |  |  |  |
| TRAF3 |  |  |  |  |
| TRAIP |  |  |  |  |
| TRBC1 |  |  |  |  |
| TREX1 |  |  |  |  |
| TRIM25 |  |  |  |  |
| TRPA1 |  |  |  |  |
| TRPV1 |  |  |  |  |
| Tryptase |  |  |  |  |
| TSBP1 |  |  |  |  |
| TSC1 |  |  |  |  |
| TSC2 |  |  |  |  |
| TSHZ1 |  |  |  |  |
| TSLP |  |  |  |  |
| TSPO |  |  |  |  |
| TTC12 |  |  |  |  |
| TTC25 |  |  |  |  |
| TTC28 |  |  |  |  |
| TTC38 |  |  |  |  |
| TTLL1 |  |  |  |  |
| TTLL3 |  |  |  |  |
| TTR |  |  |  |  |
| TULP4 |  |  |  |  |
| TXK |  |  |  |  |
| TXN |  |  |  |  |
| TXNRD2 |  |  |  |  |
| TYK2 |  |  |  |  |
| TYRO3 |  |  |  |  |
| U2AF1 |  |  |  |  |
| UBC |  |  |  |  |
| UFD1 |  |  |  |  |
| UGT1A1 |  |  |  |  |
| UGT1A8 |  |  |  |  |
| UGT1A9 |  |  |  |  |
| UGT2B15 |  |  |  |  |
| UGT2B7 |  |  |  |  |
| USB1 |  |  |  |  |
| USP38 |  |  |  |  |
| USP7 |  |  |  |  |
| UTS2R |  |  |  |  |
| VDAC1 |  |  |  |  |
| VEGFA |  |  |  |  |
| VIP |  |  |  |  |
| VIPR |  |  |  |  |
| VPS51 |  |  |  |  |
| VTN |  |  |  |  |
| VWF |  |  |  |  |
| WAC |  |  |  |  |
| WAS |  |  |  |  |
| WDPCP |  |  |  |  |
| WDR19 |  |  |  |  |
| WDR36 |  |  |  |  |
| WDR46 |  |  |  |  |
| WDR87 |  |  |  |  |
| WT1 |  |  |  |  |
| xlnA |  |  |  |  |
| XPO1 |  |  |  |  |
| XRCC1 |  |  |  |  |
| XRCC4 |  |  |  |  |
| ZBTB10 |  |  |  |  |
| ZIC2 |  |  |  |  |
| ZMYND10 |  |  |  |  |
| ZNF236 |  |  |  |  |
| ZNF469 |  |  |  |  |
| ZNF516 |  |  |  |  |
| ZPBP2 |  |  |  |  |
