# Supplementary material for: Integrated Network Pharmacology and Gut Microbiota Analysis to Explore the Mechanism of Sijunzi Decoction Involved in Alleviating Airway Inflammation in a Mouse Model of Asthma
Source: Evid Based Complement Alternat Med. 2023 Jan 3;2023:1130893. doi: 10.1155/2023/1130893 (PMC9831717; doi:10.1155/2023/1130893)
Supplement: Supplementary Materials — Supplementary Table 1: active compounds from databases and literature in Sijunzi decoction. Supplementary Table 2: asthma-related genes in the database. Supplementary Table 3: common genes of asthma and Sijunzi decoction. Supplementary Table 4: GO functional categories. Supplementary Table 5: data of KEGG enrichment analysis. [file 1130893.f1.zip › Supplementary Table 3.docx]

| **Supplementary Table 3: common genes of asthma and Sijunzi decoction** |
| --- |
| common genes |
| ABCC1 |
| ADCYAP1 |
| ADIPOQ |
| ADRA1A |
| ADRA1B |
| ADRA1D |
| ADRA2A |
| ADRB1 |
| ADRB2 |
| AKR1C1 |
| AKR1C3 |
| AKT1 |
| ALOX5 |
| ALOX5AP |
| BAX |
| BCL2 |
| CASP1 |
| CAT |
| CAV1 |
| CCL2 |
| CCND1 |
| CD40LG |
| CES1 |
| CHRM1 |
| CHRM2 |
| CHRM3 |
| CHRM4 |
| CHRM5 |
| CHRNA7 |
| CHUK |
| COL1A1 |
| CRP |
| CXCL10 |
| CXCL11 |
| CYP1A1 |
| CYP1A2 |
| CYP1B1 |
| CYP3A4 |
| DPP4 |
| EGF |
| EGFR |
| ERBB2 |
| ESR1 |
| F3 |
| GABRA2 |
| GSR |
| GSTM1 |
| HIF1A |
| HMGCR |
| HMOX1 |
| HTR2A |
| HTR3A |
| IGFBP3 |
| IL10 |
| IL1A |
| IL4 |
| INSR |
| IRF1 |
| JUN |
| KCNMA1 |
| LTA4H |
| MAOA |
| MAPK1 |
| MAPK14 |
| MAPK3 |
| MAPK8 |
| MMP1 |
| MMP2 |
| MMP3 |
| MMP9 |
| MPO |
| MYC |
| NFE2L2 |
| NFKBIA |
| NOS2 |
| NOS3 |
| NQO1 |
| NR1I2 |
| NR3C1 |
| NR3C2 |
| OPRM1 |
| PDE3A |
| PGR |
| PIK3CG |
| PLAU |
| PON1 |
| PPARG |
| PRKCA |
| PRSS1 |
| PSMD3 |
| PTEN |
| PTGER3 |
| PTGS1 |
| PTGS2 |
| SELE |
| SERPINE1 |
| SLC6A2 |
| SLC6A3 |
| SLC6A4 |
| SLPI |
| SOD1 |
| SPP1 |
| STAT3 |
| THBD |
| TNF |
| TOP2A |
| TP53 |
| UGT1A1 |
| VEGFA |
