# Supplementary material for: Integrated Network Pharmacology and Gut Microbiota Analysis to Explore the Mechanism of Sijunzi Decoction Involved in Alleviating Airway Inflammation in a Mouse Model of Asthma
Source: Evid Based Complement Alternat Med. 2023 Jan 3;2023:1130893. doi: 10.1155/2023/1130893 (PMC9831717; doi:10.1155/2023/1130893)
Supplement: Supplementary Materials — Supplementary Table 1: active compounds from databases and literature in Sijunzi decoction. Supplementary Table 2: asthma-related genes in the database. Supplementary Table 3: common genes of asthma and Sijunzi decoction. Supplementary Table 4: GO functional categories. Supplementary Table 5: data of KEGG enrichment analysis. [file 1130893.f1.zip › Supplementary Table 4.docx]

# Supplementary Table 4: GO functional categories (molecular function; biological processe; cell component)

## molecular function

| Category | Term | Count | | % | PValue |
| --- | --- | --- | --- | --- | --- |
| GOTERM_MF_DIRECT | GO:0019899~enzyme binding | | 25 | 22.93578 | 7.11E-19 |
| GOTERM_MF_DIRECT | GO:0042802~identical protein binding | | 22 | 20.18349 | 9.42E-09 |
| GOTERM_MF_DIRECT | GO:0020037~heme binding | | 11 | 10.09174 | 1.75E-08 |
| GOTERM_MF_DIRECT | GO:0042803~protein homodimerization activity | | 21 | 19.26606 | 3.28E-08 |
| GOTERM_MF_DIRECT | GO:0016907~G-protein coupled acetylcholine receptor activity | | 5 | 4.587156 | 5.46E-08 |
| GOTERM_MF_DIRECT | GO:0046982~protein heterodimerization activity | | 16 | 14.6789 | 2.67E-07 |
| GOTERM_MF_DIRECT | GO:0008144~drug binding | | 8 | 7.33945 | 5.48E-07 |
| GOTERM_MF_DIRECT | GO:0005496~steroid binding | | 6 | 5.504587 | 7.05E-07 |
| GOTERM_MF_DIRECT | GO:0005515~protein binding | | 81 | 74.31193 | 2.10E-06 |
| GOTERM_MF_DIRECT | GO:0008134~transcription factor binding | | 11 | 10.09174 | 1.41E-05 |
| GOTERM_MF_DIRECT | GO:0051721~protein phosphatase 2A binding | | 5 | 4.587156 | 2.48E-05 |
| GOTERM_MF_DIRECT | GO:0003707~steroid hormone receptor activity | | 6 | 5.504587 | 2.88E-05 |
| GOTERM_MF_DIRECT | GO:0002020~protease binding | | 7 | 6.422018 | 4.62E-05 |
| GOTERM_MF_DIRECT | GO:0019903~protein phosphatase binding | | 6 | 5.504587 | 5.11E-05 |
| GOTERM_MF_DIRECT | GO:0050661~NADP binding | | 5 | 4.587156 | 7.12E-05 |
| GOTERM_MF_DIRECT | GO:0004707~MAP kinase activity | | 4 | 3.669725 | 8.81E-05 |
| GOTERM_MF_DIRECT | GO:0005102~receptor binding | | 11 | 10.09174 | 8.86E-05 |
| GOTERM_MF_DIRECT | GO:0016712~oxidoreductase activity, acting on paired donors, with incorporation or reduction of molecular oxygen, reduced flavin or flavoprotein as one donor, and incorporation of one atom of oxygen | | 4 | 3.669725 | 1.10E-04 |
| GOTERM_MF_DIRECT | GO:0043565~sequence-specific DNA binding | | 13 | 11.92661 | 1.18E-04 |
| GOTERM_MF_DIRECT | GO:0004937~alpha1-adrenergic receptor activity | | 3 | 2.752294 | 1.21E-04 |
| GOTERM_MF_DIRECT | GO:0005125~cytokine activity | | 8 | 7.33945 | 1.37E-04 |
| GOTERM_MF_DIRECT | GO:0051380~norepinephrine binding | | 3 | 2.752294 | 2.41E-04 |
| GOTERM_MF_DIRECT | GO:0016491~oxidoreductase activity | | 8 | 7.33945 | 3.00E-04 |
| GOTERM_MF_DIRECT | GO:0051379~epinephrine binding | | 3 | 2.752294 | 5.98E-04 |
| GOTERM_MF_DIRECT | GO:0070330~aromatase activity | | 4 | 3.669725 | 6.66E-04 |
| GOTERM_MF_DIRECT | GO:0001077~transcriptional activator activity, RNA polymerase II core promoter proximal region sequence-specific binding | | 8 | 7.33945 | 8.05E-04 |
| GOTERM_MF_DIRECT | GO:0008504~monoamine transmembrane transporter activity | | 3 | 2.752294 | 8.34E-04 |
| GOTERM_MF_DIRECT | GO:0016301~kinase activity | | 8 | 7.33945 | 9.09E-04 |
| GOTERM_MF_DIRECT | GO:0030235~nitric-oxide synthase regulator activity | | 3 | 2.752294 | 0.001107 |
| GOTERM_MF_DIRECT | GO:0004252~serine-type endopeptidase activity | | 8 | 7.33945 | 0.001262 |
| GOTERM_MF_DIRECT | GO:0004879~RNA polymerase II transcription factor activity, ligand-activated sequence-specific DNA binding | | 4 | 3.669725 | 0.001559 |
| GOTERM_MF_DIRECT | GO:0004716~receptor signaling protein tyrosine kinase activity | | 3 | 2.752294 | 0.001765 |
| GOTERM_MF_DIRECT | GO:0004672~protein kinase activity | | 9 | 8.256881 | 0.002151 |
| GOTERM_MF_DIRECT | GO:0005506~iron ion binding | | 6 | 5.504587 | 0.00302 |
| GOTERM_MF_DIRECT | GO:0019825~oxygen binding | | 4 | 3.669725 | 0.003366 |
| GOTERM_MF_DIRECT | GO:0004175~endopeptidase activity | | 4 | 3.669725 | 0.004985 |
| GOTERM_MF_DIRECT | GO:0004497~monooxygenase activity | | 4 | 3.669725 | 0.006088 |
| GOTERM_MF_DIRECT | GO:0046934~phosphatidylinositol-4,5-bisphosphate 3-kinase activity | | 4 | 3.669725 | 0.007325 |
| GOTERM_MF_DIRECT | GO:0004601~peroxidase activity | | 3 | 2.752294 | 0.008617 |
| GOTERM_MF_DIRECT | GO:0032403~protein complex binding | | 6 | 5.504587 | 0.010429 |
| GOTERM_MF_DIRECT | GO:0019901~protein kinase binding | | 8 | 7.33945 | 0.010503 |
| GOTERM_MF_DIRECT | GO:0044212~transcription regulatory region DNA binding | | 6 | 5.504587 | 0.011922 |
| GOTERM_MF_DIRECT | GO:0042166~acetylcholine binding | | 3 | 2.752294 | 0.011924 |
| GOTERM_MF_DIRECT | GO:0005330~dopamine:sodium symporter activity | | 2 | 1.834862 | 0.012755 |
| GOTERM_MF_DIRECT | GO:0070576~vitamin D 24-hydroxylase activity | | 2 | 1.834862 | 0.012755 |
| GOTERM_MF_DIRECT | GO:0004051~arachidonate 5-lipoxygenase activity | | 2 | 1.834862 | 0.012755 |
| GOTERM_MF_DIRECT | GO:0004666~prostaglandin-endoperoxide synthase activity | | 2 | 1.834862 | 0.012755 |
| GOTERM_MF_DIRECT | GO:0004435~phosphatidylinositol phospholipase C activity | | 3 | 2.752294 | 0.012825 |
| GOTERM_MF_DIRECT | GO:0008237~metallopeptidase activity | | 4 | 3.669725 | 0.015146 |
| GOTERM_MF_DIRECT | GO:0005230~extracellular ligand-gated ion channel activity | | 3 | 2.752294 | 0.018819 |
| GOTERM_MF_DIRECT | GO:0047718~indanol dehydrogenase activity | | 2 | 1.834862 | 0.019072 |
| GOTERM_MF_DIRECT | GO:0004517~nitric-oxide synthase activity | | 2 | 1.834862 | 0.019072 |
| GOTERM_MF_DIRECT | GO:0047086~ketosteroid monooxygenase activity | | 2 | 1.834862 | 0.019072 |
| GOTERM_MF_DIRECT | GO:0008201~heparin binding | | 5 | 4.587156 | 0.01947 |
| GOTERM_MF_DIRECT | GO:0003700~transcription factor activity, sequence-specific DNA binding | | 13 | 11.92661 | 0.019799 |
| GOTERM_MF_DIRECT | GO:0001228~transcriptional activator activity, RNA polymerase II transcription regulatory region sequence-specific binding | | 4 | 3.669725 | 0.023051 |
| GOTERM_MF_DIRECT | GO:0004714~transmembrane receptor protein tyrosine kinase activity | | 3 | 2.752294 | 0.024545 |
| GOTERM_MF_DIRECT | GO:0047115~trans-1,2-dihydrobenzene-1,2-diol dehydrogenase activity | | 2 | 1.834862 | 0.025349 |
| GOTERM_MF_DIRECT | GO:0034875~caffeine oxidase activity | | 2 | 1.834862 | 0.025349 |
| GOTERM_MF_DIRECT | GO:0018636~phenanthrene 9,10-monooxygenase activity | | 2 | 1.834862 | 0.025349 |
| GOTERM_MF_DIRECT | GO:0034617~tetrahydrobiopterin binding | | 2 | 1.834862 | 0.025349 |
| GOTERM_MF_DIRECT | GO:0051434~BH3 domain binding | | 2 | 1.834862 | 0.025349 |
| GOTERM_MF_DIRECT | GO:0000978~RNA polymerase II core promoter proximal region sequence-specific DNA binding | | 7 | 6.422018 | 0.026516 |
| GOTERM_MF_DIRECT | GO:0042826~histone deacetylase binding | | 4 | 3.669725 | 0.027697 |
| GOTERM_MF_DIRECT | GO:0001046~core promoter sequence-specific DNA binding | | 3 | 2.752294 | 0.030885 |
| GOTERM_MF_DIRECT | GO:0048248~CXCR3 chemokine receptor binding | | 2 | 1.834862 | 0.031586 |
| GOTERM_MF_DIRECT | GO:0004784~superoxide dismutase activity | | 2 | 1.834862 | 0.031586 |
| GOTERM_MF_DIRECT | GO:0032451~demethylase activity | | 2 | 1.834862 | 0.031586 |
| GOTERM_MF_DIRECT | GO:0050544~arachidonic acid binding | | 2 | 1.834862 | 0.031586 |
| GOTERM_MF_DIRECT | GO:0004674~protein serine/threonine kinase activity | | 7 | 6.422018 | 0.033756 |
| GOTERM_MF_DIRECT | GO:0008270~zinc ion binding | | 14 | 12.84404 | 0.0357 |
| GOTERM_MF_DIRECT | GO:0004222~metalloendopeptidase activity | | 4 | 3.669725 | 0.03593 |
| GOTERM_MF_DIRECT | GO:0005088~Ras guanyl-nucleotide exchange factor activity | | 4 | 3.669725 | 0.037548 |
| GOTERM_MF_DIRECT | GO:0097110~scaffold protein binding | | 3 | 2.752294 | 0.037797 |
| GOTERM_MF_DIRECT | GO:0008009~chemokine activity | | 3 | 2.752294 | 0.039244 |
| GOTERM_MF_DIRECT | GO:0003682~chromatin binding | | 7 | 6.422018 | 0.039646 |
| GOTERM_MF_DIRECT | GO:0004032~alditol:NADP+ 1-oxidoreductase activity | | 2 | 1.834862 | 0.043941 |
| GOTERM_MF_DIRECT | GO:0034618~arginine binding | | 2 | 1.834862 | 0.043941 |
| GOTERM_MF_DIRECT | GO:0005507~copper ion binding | | 3 | 2.752294 | 0.04994 |
| GOTERM_MF_DIRECT | GO:0031995~insulin-like growth factor II binding | | 2 | 1.834862 | 0.05006 |
| GOTERM_MF_DIRECT | GO:0033691~sialic acid binding | | 2 | 1.834862 | 0.05006 |
| GOTERM_MF_DIRECT | GO:0016655~oxidoreductase activity, acting on NAD(P)H, quinone or similar compound as acceptor | | 2 | 1.834862 | 0.05006 |
| GOTERM_MF_DIRECT | GO:0004713~protein tyrosine kinase activity | | 4 | 3.669725 | 0.053742 |
| GOTERM_MF_DIRECT | GO:0051378~serotonin binding | | 2 | 1.834862 | 0.062183 |
| GOTERM_MF_DIRECT | GO:0050660~flavin adenine dinucleotide binding | | 3 | 2.752294 | 0.063279 |
| GOTERM_MF_DIRECT | GO:0015267~channel activity | | 2 | 1.834862 | 0.074151 |
| GOTERM_MF_DIRECT | GO:0031994~insulin-like growth factor I binding | | 2 | 1.834862 | 0.074151 |
| GOTERM_MF_DIRECT | GO:0001784~phosphotyrosine binding | | 2 | 1.834862 | 0.080079 |
| GOTERM_MF_DIRECT | GO:0051117~ATPase binding | | 3 | 2.752294 | 0.08141 |
| GOTERM_MF_DIRECT | GO:0008083~growth factor activity | | 4 | 3.669725 | 0.08564 |
| GOTERM_MF_DIRECT | GO:0050998~nitric-oxide synthase binding | | 2 | 1.834862 | 0.085969 |
| GOTERM_MF_DIRECT | GO:0004033~aldo-keto reductase (NADP) activity | | 2 | 1.834862 | 0.091821 |
| GOTERM_MF_DIRECT | GO:0005161~platelet-derived growth factor receptor binding | | 2 | 1.834862 | 0.091821 |
| GOTERM_MF_DIRECT | GO:0010181~FMN binding | | 2 | 1.834862 | 0.091821 |
| GOTERM_MF_DIRECT | GO:0051087~chaperone binding | | 3 | 2.752294 | 0.094934 |
| GOTERM_MF_DIRECT | GO:0003690~double-stranded DNA binding | | 3 | 2.752294 | 0.094934 |
|  |  | |  |  |  |

## biological processe

| Category | Term | Count | % | Pvalue |
| --- | --- | --- | --- | --- |
| GOTERM_BP_DIRECT | GO:0042493~response to drug | 23 | 21.10092 | 2.46E-17 |
| GOTERM_BP_DIRECT | GO:0045471~response to ethanol | 14 | 12.84404 | 1.31E-13 |
| GOTERM_BP_DIRECT | GO:0007568~aging | 16 | 14.6789 | 1.61E-13 |
| GOTERM_BP_DIRECT | GO:0001666~response to hypoxia | 16 | 14.6789 | 2.97E-13 |
| GOTERM_BP_DIRECT | GO:0001525~angiogenesis | 16 | 14.6789 | 1.29E-11 |
| GOTERM_BP_DIRECT | GO:0032496~response to lipopolysaccharide | 14 | 12.84404 | 4.21E-11 |
| GOTERM_BP_DIRECT | GO:0045944~positive regulation of transcription from RNA polymerase II promoter | 28 | 25.68807 | 7.73E-11 |
| GOTERM_BP_DIRECT | GO:0032355~response to estradiol | 11 | 10.09174 | 3.3E-10 |
| GOTERM_BP_DIRECT | GO:0045907~positive regulation of vasoconstriction | 8 | 7.33945 | 1.1E-09 |
| GOTERM_BP_DIRECT | GO:0031663~lipopolysaccharide-mediated signaling pathway | 8 | 7.33945 | 1.1E-09 |
| GOTERM_BP_DIRECT | GO:0070374~positive regulation of ERK1 and ERK2 cascade | 13 | 11.92661 | 1.32E-09 |
| GOTERM_BP_DIRECT | GO:0010628~positive regulation of gene expression | 15 | 13.76147 | 1.36E-09 |
| GOTERM_BP_DIRECT | GO:0006954~inflammatory response | 17 | 15.59633 | 2.55E-09 |
| GOTERM_BP_DIRECT | GO:0007271~synaptic transmission, cholinergic | 8 | 7.33945 | 3.28E-09 |
| GOTERM_BP_DIRECT | GO:0045766~positive regulation of angiogenesis | 11 | 10.09174 | 3.37E-09 |
| GOTERM_BP_DIRECT | GO:0009636~response to toxic substance | 10 | 9.174312 | 3.68E-09 |
| GOTERM_BP_DIRECT | GO:0043066~negative regulation of apoptotic process | 18 | 16.51376 | 4.85E-09 |
| GOTERM_BP_DIRECT | GO:0008284~positive regulation of cell proliferation | 18 | 16.51376 | 6.93E-09 |
| GOTERM_BP_DIRECT | GO:0008283~cell proliferation | 16 | 14.6789 | 1.25E-08 |
| GOTERM_BP_DIRECT | GO:0007165~signal transduction | 27 | 24.77064 | 1.46E-08 |
| GOTERM_BP_DIRECT | GO:0007584~response to nutrient | 9 | 8.256881 | 2.39E-08 |
| GOTERM_BP_DIRECT | GO:0007197~adenylate cyclase-inhibiting G-protein coupled acetylcholine receptor signaling pathway | 5 | 4.587156 | 5.58E-08 |
| GOTERM_BP_DIRECT | GO:0043406~positive regulation of MAP kinase activity | 8 | 7.33945 | 9.69E-08 |
| GOTERM_BP_DIRECT | GO:0071880~adenylate cyclase-activating adrenergic receptor signaling pathway | 6 | 5.504587 | 1.09E-07 |
| GOTERM_BP_DIRECT | GO:0048661~positive regulation of smooth muscle cell proliferation | 8 | 7.33945 | 1.09E-07 |
| GOTERM_BP_DIRECT | GO:0007207~phospholipase C-activating G-protein coupled acetylcholine receptor signaling pathway | 5 | 4.587156 | 1.11E-07 |
| GOTERM_BP_DIRECT | GO:0001934~positive regulation of protein phosphorylation | 10 | 9.174312 | 1.27E-07 |
| GOTERM_BP_DIRECT | GO:0045893~positive regulation of transcription, DNA-templated | 17 | 15.59633 | 1.83E-07 |
| GOTERM_BP_DIRECT | GO:0071456~cellular response to hypoxia | 9 | 8.256881 | 1.87E-07 |
| GOTERM_BP_DIRECT | GO:0045429~positive regulation of nitric oxide biosynthetic process | 7 | 6.422018 | 3.09E-07 |
| GOTERM_BP_DIRECT | GO:0071260~cellular response to mechanical stimulus | 8 | 7.33945 | 3.55E-07 |
| GOTERM_BP_DIRECT | GO:0051090~regulation of sequence-specific DNA binding transcription factor activity | 6 | 5.504587 | 4.81E-07 |
| GOTERM_BP_DIRECT | GO:0006805~xenobiotic metabolic process | 8 | 7.33945 | 6.79E-07 |
| GOTERM_BP_DIRECT | GO:0097421~liver regeneration | 6 | 5.504587 | 1.05E-06 |
| GOTERM_BP_DIRECT | GO:0006809~nitric oxide biosynthetic process | 5 | 4.587156 | 1.11E-06 |
| GOTERM_BP_DIRECT | GO:0055114~oxidation-reduction process | 17 | 15.59633 | 1.17E-06 |
| GOTERM_BP_DIRECT | GO:0010332~response to gamma radiation | 6 | 5.504587 | 1.49E-06 |
| GOTERM_BP_DIRECT | GO:0007213~G-protein coupled acetylcholine receptor signaling pathway | 5 | 4.587156 | 2.09E-06 |
| GOTERM_BP_DIRECT | GO:0071407~cellular response to organic cyclic compound | 7 | 6.422018 | 2.1E-06 |
| GOTERM_BP_DIRECT | GO:0043065~positive regulation of apoptotic process | 12 | 11.00917 | 3.65E-06 |
| GOTERM_BP_DIRECT | GO:0042593~glucose homeostasis | 8 | 7.33945 | 3.92E-06 |
| GOTERM_BP_DIRECT | GO:0007200~phospholipase C-activating G-protein coupled receptor signaling pathway | 7 | 6.422018 | 4.09E-06 |
| GOTERM_BP_DIRECT | GO:0001938~positive regulation of endothelial cell proliferation | 7 | 6.422018 | 5.31E-06 |
| GOTERM_BP_DIRECT | GO:0035690~cellular response to drug | 7 | 6.422018 | 5.31E-06 |
| GOTERM_BP_DIRECT | GO:0000187~activation of MAPK activity | 8 | 7.33945 | 5.75E-06 |
| GOTERM_BP_DIRECT | GO:0043536~positive regulation of blood vessel endothelial cell migration | 5 | 4.587156 | 5.82E-06 |
| GOTERM_BP_DIRECT | GO:0001541~ovarian follicle development | 6 | 5.504587 | 7.06E-06 |
| GOTERM_BP_DIRECT | GO:0008202~steroid metabolic process | 6 | 5.504587 | 7.94E-06 |
| GOTERM_BP_DIRECT | GO:0071222~cellular response to lipopolysaccharide | 8 | 7.33945 | 8.26E-06 |
| GOTERM_BP_DIRECT | GO:0032094~response to food | 5 | 4.587156 | 8.9E-06 |
| GOTERM_BP_DIRECT | GO:0045987~positive regulation of smooth muscle contraction | 5 | 4.587156 | 8.9E-06 |
| GOTERM_BP_DIRECT | GO:0008285~negative regulation of cell proliferation | 13 | 11.92661 | 9.09E-06 |
| GOTERM_BP_DIRECT | GO:0030168~platelet activation | 8 | 7.33945 | 9.27E-06 |
| GOTERM_BP_DIRECT | GO:0042542~response to hydrogen peroxide | 6 | 5.504587 | 1.86E-05 |
| GOTERM_BP_DIRECT | GO:0030335~positive regulation of cell migration | 9 | 8.256881 | 2.47E-05 |
| GOTERM_BP_DIRECT | GO:0007204~positive regulation of cytosolic calcium ion concentration | 8 | 7.33945 | 2.51E-05 |
| GOTERM_BP_DIRECT | GO:0045909~positive regulation of vasodilation | 5 | 4.587156 | 3.4E-05 |
| GOTERM_BP_DIRECT | GO:0007267~cell-cell signaling | 10 | 9.174312 | 3.82E-05 |
| GOTERM_BP_DIRECT | GO:0046326~positive regulation of glucose import | 5 | 4.587156 | 3.9E-05 |
| GOTERM_BP_DIRECT | GO:0000165~MAPK cascade | 10 | 9.174312 | 4.87E-05 |
| GOTERM_BP_DIRECT | GO:0019430~removal of superoxide radicals | 4 | 3.669725 | 5.46E-05 |
| GOTERM_BP_DIRECT | GO:0043950~positive regulation of cAMP-mediated signaling | 4 | 3.669725 | 5.46E-05 |
| GOTERM_BP_DIRECT | GO:0071364~cellular response to epidermal growth factor stimulus | 5 | 4.587156 | 5.74E-05 |
| GOTERM_BP_DIRECT | GO:0051091~positive regulation of sequence-specific DNA binding transcription factor activity | 7 | 6.422018 | 5.91E-05 |
| GOTERM_BP_DIRECT | GO:0043627~response to estrogen | 6 | 5.504587 | 6.09E-05 |
| GOTERM_BP_DIRECT | GO:0008217~regulation of blood pressure | 6 | 5.504587 | 6.09E-05 |
| GOTERM_BP_DIRECT | GO:0010745~negative regulation of macrophage derived foam cell differentiation | 4 | 3.669725 | 7.06E-05 |
| GOTERM_BP_DIRECT | GO:0045019~negative regulation of nitric oxide biosynthetic process | 4 | 3.669725 | 7.06E-05 |
| GOTERM_BP_DIRECT | GO:0006915~apoptotic process | 14 | 12.84404 | 7.06E-05 |
| GOTERM_BP_DIRECT | GO:0032461~positive regulation of protein oligomerization | 4 | 3.669725 | 8.94E-05 |
| GOTERM_BP_DIRECT | GO:0045821~positive regulation of glycolytic process | 4 | 3.669725 | 8.94E-05 |
| GOTERM_BP_DIRECT | GO:0006691~leukotriene metabolic process | 4 | 3.669725 | 0.000111 |
| GOTERM_BP_DIRECT | GO:0007623~circadian rhythm | 6 | 5.504587 | 0.000121 |
| GOTERM_BP_DIRECT | GO:0031281~positive regulation of cyclase activity | 3 | 2.752294 | 0.000122 |
| GOTERM_BP_DIRECT | GO:0070141~response to UV-A | 3 | 2.752294 | 0.000122 |
| GOTERM_BP_DIRECT | GO:0034465~response to carbon monoxide | 3 | 2.752294 | 0.000122 |
| GOTERM_BP_DIRECT | GO:0022617~extracellular matrix disassembly | 6 | 5.504587 | 0.000129 |
| GOTERM_BP_DIRECT | GO:0006935~chemotaxis | 7 | 6.422018 | 0.000136 |
| GOTERM_BP_DIRECT | GO:0006940~regulation of smooth muscle contraction | 4 | 3.669725 | 0.000136 |
| GOTERM_BP_DIRECT | GO:0032869~cellular response to insulin stimulus | 6 | 5.504587 | 0.000137 |
| GOTERM_BP_DIRECT | GO:0014066~regulation of phosphatidylinositol 3-kinase signaling | 6 | 5.504587 | 0.000146 |
| GOTERM_BP_DIRECT | GO:0007566~embryo implantation | 5 | 4.587156 | 0.00015 |
| GOTERM_BP_DIRECT | GO:0007595~lactation | 5 | 4.587156 | 0.00015 |
| GOTERM_BP_DIRECT | GO:0018105~peptidyl-serine phosphorylation | 7 | 6.422018 | 0.000155 |
| GOTERM_BP_DIRECT | GO:0071276~cellular response to cadmium ion | 4 | 3.669725 | 0.000165 |
| GOTERM_BP_DIRECT | GO:0060749~mammary gland alveolus development | 4 | 3.669725 | 0.000165 |
| GOTERM_BP_DIRECT | GO:0043085~positive regulation of catalytic activity | 6 | 5.504587 | 0.000174 |
| GOTERM_BP_DIRECT | GO:0042127~regulation of cell proliferation | 8 | 7.33945 | 0.000192 |
| GOTERM_BP_DIRECT | GO:0051897~positive regulation of protein kinase B signaling | 6 | 5.504587 | 0.000207 |
| GOTERM_BP_DIRECT | GO:0051591~response to cAMP | 5 | 4.587156 | 0.000214 |
| GOTERM_BP_DIRECT | GO:0001994~norepinephrine-epinephrine vasoconstriction involved in regulation of systemic arterial blood pressure | 3 | 2.752294 | 0.000244 |
| GOTERM_BP_DIRECT | GO:0019233~sensory perception of pain | 5 | 4.587156 | 0.000345 |
| GOTERM_BP_DIRECT | GO:0006974~cellular response to DNA damage stimulus | 8 | 7.33945 | 0.000392 |
| GOTERM_BP_DIRECT | GO:0031622~positive regulation of fever generation | 3 | 2.752294 | 0.000405 |
| GOTERM_BP_DIRECT | GO:0072584~caveolin-mediated endocytosis | 3 | 2.752294 | 0.000405 |
| GOTERM_BP_DIRECT | GO:0043401~steroid hormone mediated signaling pathway | 5 | 4.587156 | 0.000492 |
| GOTERM_BP_DIRECT | GO:0050999~regulation of nitric-oxide synthase activity | 4 | 3.669725 | 0.000604 |
| GOTERM_BP_DIRECT | GO:0007202~activation of phospholipase C activity | 4 | 3.669725 | 0.000604 |
| GOTERM_BP_DIRECT | GO:0046483~heterocycle metabolic process | 3 | 2.752294 | 0.000605 |
| GOTERM_BP_DIRECT | GO:0048015~phosphatidylinositol-mediated signaling | 6 | 5.504587 | 0.000607 |
| GOTERM_BP_DIRECT | GO:0007186~G-protein coupled receptor signaling pathway | 16 | 14.6789 | 0.000625 |
| GOTERM_BP_DIRECT | GO:0017144~drug metabolic process | 4 | 3.669725 | 0.000676 |
| GOTERM_BP_DIRECT | GO:0010575~positive regulation of vascular endothelial growth factor production | 4 | 3.669725 | 0.000676 |
| GOTERM_BP_DIRECT | GO:0007613~memory | 5 | 4.587156 | 0.000678 |
| GOTERM_BP_DIRECT | GO:0006468~protein phosphorylation | 11 | 10.09174 | 0.000713 |
| GOTERM_BP_DIRECT | GO:0006979~response to oxidative stress | 6 | 5.504587 | 0.000718 |
| GOTERM_BP_DIRECT | GO:0000060~protein import into nucleus, translocation | 4 | 3.669725 | 0.000754 |
| GOTERM_BP_DIRECT | GO:2000352~negative regulation of endothelial cell apoptotic process | 4 | 3.669725 | 0.000754 |
| GOTERM_BP_DIRECT | GO:0030574~collagen catabolic process | 5 | 4.587156 | 0.000764 |
| GOTERM_BP_DIRECT | GO:0051384~response to glucocorticoid | 5 | 4.587156 | 0.00081 |
| GOTERM_BP_DIRECT | GO:0043542~endothelial cell migration | 4 | 3.669725 | 0.000837 |
| GOTERM_BP_DIRECT | GO:0048662~negative regulation of smooth muscle cell proliferation | 4 | 3.669725 | 0.000837 |
| GOTERM_BP_DIRECT | GO:0006006~glucose metabolic process | 5 | 4.587156 | 0.000908 |
| GOTERM_BP_DIRECT | GO:2000379~positive regulation of reactive oxygen species metabolic process | 4 | 3.669725 | 0.000926 |
| GOTERM_BP_DIRECT | GO:0071392~cellular response to estradiol stimulus | 4 | 3.669725 | 0.00102 |
| GOTERM_BP_DIRECT | GO:0038095~Fc-epsilon receptor signaling pathway | 7 | 6.422018 | 0.001022 |
| GOTERM_BP_DIRECT | GO:0033138~positive regulation of peptidyl-serine phosphorylation | 5 | 4.587156 | 0.001071 |
| GOTERM_BP_DIRECT | GO:0071300~cellular response to retinoic acid | 5 | 4.587156 | 0.001071 |
| GOTERM_BP_DIRECT | GO:0010818~T cell chemotaxis | 3 | 2.752294 | 0.001119 |
| GOTERM_BP_DIRECT | GO:0010888~negative regulation of lipid storage | 3 | 2.752294 | 0.001119 |
| GOTERM_BP_DIRECT | GO:0015844~monoamine transport | 3 | 2.752294 | 0.001119 |
| GOTERM_BP_DIRECT | GO:0050873~brown fat cell differentiation | 4 | 3.669725 | 0.00112 |
| GOTERM_BP_DIRECT | GO:0046677~response to antibiotic | 4 | 3.669725 | 0.00112 |
| GOTERM_BP_DIRECT | GO:0050900~leukocyte migration | 6 | 5.504587 | 0.001145 |
| GOTERM_BP_DIRECT | GO:0043491~protein kinase B signaling | 4 | 3.669725 | 0.001227 |
| GOTERM_BP_DIRECT | GO:2001244~positive regulation of intrinsic apoptotic signaling pathway | 4 | 3.669725 | 0.001227 |
| GOTERM_BP_DIRECT | GO:0097192~extrinsic apoptotic signaling pathway in absence of ligand | 4 | 3.669725 | 0.001339 |
| GOTERM_BP_DIRECT | GO:0050665~hydrogen peroxide biosynthetic process | 3 | 2.752294 | 0.001433 |
| GOTERM_BP_DIRECT | GO:0097267~omega-hydroxylase P450 pathway | 3 | 2.752294 | 0.001433 |
| GOTERM_BP_DIRECT | GO:0032930~positive regulation of superoxide anion generation | 3 | 2.752294 | 0.001433 |
| GOTERM_BP_DIRECT | GO:0006955~immune response | 10 | 9.174312 | 0.001576 |
| GOTERM_BP_DIRECT | GO:0009409~response to cold | 4 | 3.669725 | 0.001583 |
| GOTERM_BP_DIRECT | GO:0043524~negative regulation of neuron apoptotic process | 6 | 5.504587 | 0.001624 |
| GOTERM_BP_DIRECT | GO:0035094~response to nicotine | 4 | 3.669725 | 0.001715 |
| GOTERM_BP_DIRECT | GO:0007188~adenylate cyclase-modulating G-protein coupled receptor signaling pathway | 4 | 3.669725 | 0.001715 |
| GOTERM_BP_DIRECT | GO:2001240~negative regulation of extrinsic apoptotic signaling pathway in absence of ligand | 4 | 3.669725 | 0.001715 |
| GOTERM_BP_DIRECT | GO:0045080~positive regulation of chemokine biosynthetic process | 3 | 2.752294 | 0.001783 |
| GOTERM_BP_DIRECT | GO:0019371~cyclooxygenase pathway | 3 | 2.752294 | 0.001783 |
| GOTERM_BP_DIRECT | GO:0051146~striated muscle cell differentiation | 3 | 2.752294 | 0.001783 |
| GOTERM_BP_DIRECT | GO:0046902~regulation of mitochondrial membrane permeability | 3 | 2.752294 | 0.001783 |
| GOTERM_BP_DIRECT | GO:0043267~negative regulation of potassium ion transport | 3 | 2.752294 | 0.001783 |
| GOTERM_BP_DIRECT | GO:0043410~positive regulation of MAPK cascade | 5 | 4.587156 | 0.001841 |
| GOTERM_BP_DIRECT | GO:0038128~ERBB2 signaling pathway | 4 | 3.669725 | 0.001853 |
| GOTERM_BP_DIRECT | GO:0018107~peptidyl-threonine phosphorylation | 4 | 3.669725 | 0.001853 |
| GOTERM_BP_DIRECT | GO:0006919~activation of cysteine-type endopeptidase activity involved in apoptotic process | 5 | 4.587156 | 0.002013 |
| GOTERM_BP_DIRECT | GO:0007190~activation of adenylate cyclase activity | 4 | 3.669725 | 0.00215 |
| GOTERM_BP_DIRECT | GO:0030278~regulation of ossification | 3 | 2.752294 | 0.00217 |
| GOTERM_BP_DIRECT | GO:0042574~retinal metabolic process | 3 | 2.752294 | 0.002594 |
| GOTERM_BP_DIRECT | GO:0042738~exogenous drug catabolic process | 3 | 2.752294 | 0.002594 |
| GOTERM_BP_DIRECT | GO:0043525~positive regulation of neuron apoptotic process | 4 | 3.669725 | 0.002648 |
| GOTERM_BP_DIRECT | GO:0014823~response to activity | 4 | 3.669725 | 0.002648 |
| GOTERM_BP_DIRECT | GO:0048538~thymus development | 4 | 3.669725 | 0.002648 |
| GOTERM_BP_DIRECT | GO:0045785~positive regulation of cell adhesion | 4 | 3.669725 | 0.002648 |
| GOTERM_BP_DIRECT | GO:0034644~cellular response to UV | 4 | 3.669725 | 0.002829 |
| GOTERM_BP_DIRECT | GO:0006879~cellular iron ion homeostasis | 4 | 3.669725 | 0.002829 |
| GOTERM_BP_DIRECT | GO:0006367~transcription initiation from RNA polymerase II promoter | 6 | 5.504587 | 0.003003 |
| GOTERM_BP_DIRECT | GO:0071363~cellular response to growth factor stimulus | 4 | 3.669725 | 0.003017 |
| GOTERM_BP_DIRECT | GO:0051926~negative regulation of calcium ion transport | 3 | 2.752294 | 0.003052 |
| GOTERM_BP_DIRECT | GO:0019372~lipoxygenase pathway | 3 | 2.752294 | 0.003052 |
| GOTERM_BP_DIRECT | GO:0048546~digestive tract morphogenesis | 3 | 2.752294 | 0.003052 |
| GOTERM_BP_DIRECT | GO:0071318~cellular response to ATP | 3 | 2.752294 | 0.003052 |
| GOTERM_BP_DIRECT | GO:0030336~negative regulation of cell migration | 5 | 4.587156 | 0.003289 |
| GOTERM_BP_DIRECT | GO:0071230~cellular response to amino acid stimulus | 4 | 3.669725 | 0.003416 |
| GOTERM_BP_DIRECT | GO:0008630~intrinsic apoptotic signaling pathway in response to DNA damage | 4 | 3.669725 | 0.003416 |
| GOTERM_BP_DIRECT | GO:0010875~positive regulation of cholesterol efflux | 3 | 2.752294 | 0.003546 |
| GOTERM_BP_DIRECT | GO:0019395~fatty acid oxidation | 3 | 2.752294 | 0.003546 |
| GOTERM_BP_DIRECT | GO:0014912~negative regulation of smooth muscle cell migration | 3 | 2.752294 | 0.003546 |
| GOTERM_BP_DIRECT | GO:0009408~response to heat | 4 | 3.669725 | 0.003627 |
| GOTERM_BP_DIRECT | GO:0043123~positive regulation of I-kappaB kinase/NF-kappaB signaling | 6 | 5.504587 | 0.00384 |
| GOTERM_BP_DIRECT | GO:0010468~regulation of gene expression | 5 | 4.587156 | 0.003953 |
| GOTERM_BP_DIRECT | GO:0032270~positive regulation of cellular protein metabolic process | 3 | 2.752294 | 0.004075 |
| GOTERM_BP_DIRECT | GO:0060397~JAK-STAT cascade involved in growth hormone signaling pathway | 3 | 2.752294 | 0.004075 |
| GOTERM_BP_DIRECT | GO:1904707~positive regulation of vascular smooth muscle cell proliferation | 3 | 2.752294 | 0.004075 |
| GOTERM_BP_DIRECT | GO:0001649~osteoblast differentiation | 5 | 4.587156 | 0.004546 |
| GOTERM_BP_DIRECT | GO:0032880~regulation of protein localization | 4 | 3.669725 | 0.004549 |
| GOTERM_BP_DIRECT | GO:0090026~positive regulation of monocyte chemotaxis | 3 | 2.752294 | 0.004637 |
| GOTERM_BP_DIRECT | GO:0044130~negative regulation of growth of symbiont in host | 3 | 2.752294 | 0.004637 |
| GOTERM_BP_DIRECT | GO:0035994~response to muscle stretch | 3 | 2.752294 | 0.004637 |
| GOTERM_BP_DIRECT | GO:0048146~positive regulation of fibroblast proliferation | 4 | 3.669725 | 0.005058 |
| GOTERM_BP_DIRECT | GO:0030224~monocyte differentiation | 3 | 2.752294 | 0.005233 |
| GOTERM_BP_DIRECT | GO:0055093~response to hyperoxia | 3 | 2.752294 | 0.005233 |
| GOTERM_BP_DIRECT | GO:0070542~response to fatty acid | 3 | 2.752294 | 0.005233 |
| GOTERM_BP_DIRECT | GO:0051260~protein homooligomerization | 6 | 5.504587 | 0.005723 |
| GOTERM_BP_DIRECT | GO:0019373~epoxygenase P450 pathway | 3 | 2.752294 | 0.005863 |
| GOTERM_BP_DIRECT | GO:0010039~response to iron ion | 3 | 2.752294 | 0.005863 |
| GOTERM_BP_DIRECT | GO:0033189~response to vitamin A | 3 | 2.752294 | 0.005863 |
| GOTERM_BP_DIRECT | GO:0033280~response to vitamin D | 3 | 2.752294 | 0.005863 |
| GOTERM_BP_DIRECT | GO:0006939~smooth muscle contraction | 3 | 2.752294 | 0.005863 |
| GOTERM_BP_DIRECT | GO:0032091~negative regulation of protein binding | 4 | 3.669725 | 0.005884 |
| GOTERM_BP_DIRECT | GO:0051592~response to calcium ion | 4 | 3.669725 | 0.006177 |
| GOTERM_BP_DIRECT | GO:0009612~response to mechanical stimulus | 4 | 3.669725 | 0.006477 |
| GOTERM_BP_DIRECT | GO:0001659~temperature homeostasis | 3 | 2.752294 | 0.006525 |
| GOTERM_BP_DIRECT | GO:0051968~positive regulation of synaptic transmission, glutamatergic | 3 | 2.752294 | 0.006525 |
| GOTERM_BP_DIRECT | GO:0050679~positive regulation of epithelial cell proliferation | 4 | 3.669725 | 0.006787 |
| GOTERM_BP_DIRECT | GO:0019370~leukotriene biosynthetic process | 3 | 2.752294 | 0.00722 |
| GOTERM_BP_DIRECT | GO:1902895~positive regulation of pri-miRNA transcription from RNA polymerase II promoter | 3 | 2.752294 | 0.00722 |
| GOTERM_BP_DIRECT | GO:0050727~regulation of inflammatory response | 4 | 3.669725 | 0.007767 |
| GOTERM_BP_DIRECT | GO:0035902~response to immobilization stress | 3 | 2.752294 | 0.007947 |
| GOTERM_BP_DIRECT | GO:0007166~cell surface receptor signaling pathway | 7 | 6.422018 | 0.008551 |
| GOTERM_BP_DIRECT | GO:0051000~positive regulation of nitric-oxide synthase activity | 3 | 2.752294 | 0.008705 |
| GOTERM_BP_DIRECT | GO:0010165~response to X-ray | 3 | 2.752294 | 0.008705 |
| GOTERM_BP_DIRECT | GO:0050796~regulation of insulin secretion | 4 | 3.669725 | 0.009199 |
| GOTERM_BP_DIRECT | GO:0001836~release of cytochrome c from mitochondria | 3 | 2.752294 | 0.009494 |
| GOTERM_BP_DIRECT | GO:0007194~negative regulation of adenylate cyclase activity | 3 | 2.752294 | 0.009494 |
| GOTERM_BP_DIRECT | GO:0009617~response to bacterium | 3 | 2.752294 | 0.009494 |
| GOTERM_BP_DIRECT | GO:0035924~cellular response to vascular endothelial growth factor stimulus | 3 | 2.752294 | 0.009494 |
| GOTERM_BP_DIRECT | GO:0001819~positive regulation of cytokine production | 3 | 2.752294 | 0.010314 |
| GOTERM_BP_DIRECT | GO:0070371~ERK1 and ERK2 cascade | 3 | 2.752294 | 0.010314 |
| GOTERM_BP_DIRECT | GO:0098869~cellular oxidant detoxification | 4 | 3.669725 | 0.010366 |
| GOTERM_BP_DIRECT | GO:0007265~Ras protein signal transduction | 4 | 3.669725 | 0.010366 |
| GOTERM_BP_DIRECT | GO:0030855~epithelial cell differentiation | 4 | 3.669725 | 0.010366 |
| GOTERM_BP_DIRECT | GO:0051092~positive regulation of NF-kappaB transcription factor activity | 5 | 4.587156 | 0.010695 |
| GOTERM_BP_DIRECT | GO:0007569~cell aging | 3 | 2.752294 | 0.011165 |
| GOTERM_BP_DIRECT | GO:0050715~positive regulation of cytokine secretion | 3 | 2.752294 | 0.011165 |
| GOTERM_BP_DIRECT | GO:0034612~response to tumor necrosis factor | 3 | 2.752294 | 0.011165 |
| GOTERM_BP_DIRECT | GO:0051899~membrane depolarization | 3 | 2.752294 | 0.011165 |
| GOTERM_BP_DIRECT | GO:0010629~negative regulation of gene expression | 5 | 4.587156 | 0.011825 |
| GOTERM_BP_DIRECT | GO:0032757~positive regulation of interleukin-8 production | 3 | 2.752294 | 0.012045 |
| GOTERM_BP_DIRECT | GO:0045840~positive regulation of mitotic nuclear division | 3 | 2.752294 | 0.012045 |
| GOTERM_BP_DIRECT | GO:0016032~viral process | 7 | 6.422018 | 0.012768 |
| GOTERM_BP_DIRECT | GO:0071306~cellular response to vitamin E | 2 | 1.834862 | 0.012822 |
| GOTERM_BP_DIRECT | GO:0001997~positive regulation of the force of heart contraction by epinephrine-norepinephrine | 2 | 1.834862 | 0.012822 |
| GOTERM_BP_DIRECT | GO:0001996~positive regulation of heart rate by epinephrine-norepinephrine | 2 | 1.834862 | 0.012822 |
| GOTERM_BP_DIRECT | GO:0014806~smooth muscle hyperplasia | 2 | 1.834862 | 0.012822 |
| GOTERM_BP_DIRECT | GO:0060073~micturition | 2 | 1.834862 | 0.012822 |
| GOTERM_BP_DIRECT | GO:0006808~regulation of nitrogen utilization | 2 | 1.834862 | 0.012822 |
| GOTERM_BP_DIRECT | GO:0003057~regulation of the force of heart contraction by chemical signal | 2 | 1.834862 | 0.012822 |
| GOTERM_BP_DIRECT | GO:0002540~leukotriene production involved in inflammatory response | 2 | 1.834862 | 0.012822 |
| GOTERM_BP_DIRECT | GO:0032227~negative regulation of synaptic transmission, dopaminergic | 2 | 1.834862 | 0.012822 |
| GOTERM_BP_DIRECT | GO:0001912~positive regulation of leukocyte mediated cytotoxicity | 2 | 1.834862 | 0.012822 |
| GOTERM_BP_DIRECT | GO:0030324~lung development | 4 | 3.669725 | 0.01295 |
| GOTERM_BP_DIRECT | GO:0014065~phosphatidylinositol 3-kinase signaling | 3 | 2.752294 | 0.012954 |
| GOTERM_BP_DIRECT | GO:0051281~positive regulation of release of sequestered calcium ion into cytosol | 3 | 2.752294 | 0.012954 |
| GOTERM_BP_DIRECT | GO:0045454~cell redox homeostasis | 4 | 3.669725 | 0.013413 |
| GOTERM_BP_DIRECT | GO:0031295~T cell costimulation | 4 | 3.669725 | 0.013885 |
| GOTERM_BP_DIRECT | GO:0009314~response to radiation | 3 | 2.752294 | 0.013893 |
| GOTERM_BP_DIRECT | GO:0043388~positive regulation of DNA binding | 3 | 2.752294 | 0.013893 |
| GOTERM_BP_DIRECT | GO:0090200~positive regulation of release of cytochrome c from mitochondria | 3 | 2.752294 | 0.013893 |
| GOTERM_BP_DIRECT | GO:2000145~regulation of cell motility | 3 | 2.752294 | 0.013893 |
| GOTERM_BP_DIRECT | GO:0043392~negative regulation of DNA binding | 3 | 2.752294 | 0.013893 |
| GOTERM_BP_DIRECT | GO:0032715~negative regulation of interleukin-6 production | 3 | 2.752294 | 0.013893 |
| GOTERM_BP_DIRECT | GO:0051881~regulation of mitochondrial membrane potential | 3 | 2.752294 | 0.013893 |
| GOTERM_BP_DIRECT | GO:0001503~ossification | 4 | 3.669725 | 0.014859 |
| GOTERM_BP_DIRECT | GO:0051973~positive regulation of telomerase activity | 3 | 2.752294 | 0.01486 |
| GOTERM_BP_DIRECT | GO:0042149~cellular response to glucose starvation | 3 | 2.752294 | 0.01486 |
| GOTERM_BP_DIRECT | GO:0001662~behavioral fear response | 3 | 2.752294 | 0.01486 |
| GOTERM_BP_DIRECT | GO:0050852~T cell receptor signaling pathway | 5 | 4.587156 | 0.015319 |
| GOTERM_BP_DIRECT | GO:0006508~proteolysis | 9 | 8.256881 | 0.015365 |
| GOTERM_BP_DIRECT | GO:1900740~positive regulation of protein insertion into mitochondrial membrane involved in apoptotic signaling pathway | 3 | 2.752294 | 0.015855 |
| GOTERM_BP_DIRECT | GO:0045776~negative regulation of blood pressure | 3 | 2.752294 | 0.015855 |
| GOTERM_BP_DIRECT | GO:0044344~cellular response to fibroblast growth factor stimulus | 3 | 2.752294 | 0.015855 |
| GOTERM_BP_DIRECT | GO:0050731~positive regulation of peptidyl-tyrosine phosphorylation | 4 | 3.669725 | 0.015869 |
| GOTERM_BP_DIRECT | GO:0060070~canonical Wnt signaling pathway | 4 | 3.669725 | 0.016389 |
| GOTERM_BP_DIRECT | GO:0048147~negative regulation of fibroblast proliferation | 3 | 2.752294 | 0.016878 |
| GOTERM_BP_DIRECT | GO:0007626~locomotory behavior | 4 | 3.669725 | 0.016918 |
| GOTERM_BP_DIRECT | GO:0030307~positive regulation of cell growth | 4 | 3.669725 | 0.016918 |
| GOTERM_BP_DIRECT | GO:0042220~response to cocaine | 3 | 2.752294 | 0.017929 |
| GOTERM_BP_DIRECT | GO:0042100~B cell proliferation | 3 | 2.752294 | 0.017929 |
| GOTERM_BP_DIRECT | GO:0006928~movement of cell or subcellular component | 4 | 3.669725 | 0.018005 |
| GOTERM_BP_DIRECT | GO:1902042~negative regulation of extrinsic apoptotic signaling pathway via death domain receptors | 3 | 2.752294 | 0.019006 |
| GOTERM_BP_DIRECT | GO:0000189~MAPK import into nucleus | 2 | 1.834862 | 0.019172 |
| GOTERM_BP_DIRECT | GO:0002679~respiratory burst involved in defense response | 2 | 1.834862 | 0.019172 |
| GOTERM_BP_DIRECT | GO:0019087~transformation of host cell by virus | 2 | 1.834862 | 0.019172 |
| GOTERM_BP_DIRECT | GO:0061308~cardiac neural crest cell development involved in heart development | 2 | 1.834862 | 0.019172 |
| GOTERM_BP_DIRECT | GO:0002904~positive regulation of B cell apoptotic process | 2 | 1.834862 | 0.019172 |
| GOTERM_BP_DIRECT | GO:0032800~receptor biosynthetic process | 2 | 1.834862 | 0.019172 |
| GOTERM_BP_DIRECT | GO:0031649~heat generation | 2 | 1.834862 | 0.019172 |
| GOTERM_BP_DIRECT | GO:0002025~vasodilation by norepinephrine-epinephrine involved in regulation of systemic arterial blood pressure | 2 | 1.834862 | 0.019172 |
| GOTERM_BP_DIRECT | GO:0014854~response to inactivity | 2 | 1.834862 | 0.019172 |
| GOTERM_BP_DIRECT | GO:0007565~female pregnancy | 4 | 3.669725 | 0.019706 |
| GOTERM_BP_DIRECT | GO:0043330~response to exogenous dsRNA | 3 | 2.752294 | 0.02011 |
| GOTERM_BP_DIRECT | GO:0045787~positive regulation of cell cycle | 3 | 2.752294 | 0.02011 |
| GOTERM_BP_DIRECT | GO:0042594~response to starvation | 3 | 2.752294 | 0.021241 |
| GOTERM_BP_DIRECT | GO:0001890~placenta development | 3 | 2.752294 | 0.021241 |
| GOTERM_BP_DIRECT | GO:0006874~cellular calcium ion homeostasis | 4 | 3.669725 | 0.022108 |
| GOTERM_BP_DIRECT | GO:0046854~phosphatidylinositol phosphorylation | 4 | 3.669725 | 0.022733 |
| GOTERM_BP_DIRECT | GO:0008584~male gonad development | 4 | 3.669725 | 0.022733 |
| GOTERM_BP_DIRECT | GO:0034605~cellular response to heat | 3 | 2.752294 | 0.023579 |
| GOTERM_BP_DIRECT | GO:0032720~negative regulation of tumor necrosis factor production | 3 | 2.752294 | 0.024786 |
| GOTERM_BP_DIRECT | GO:0008625~extrinsic apoptotic signaling pathway via death domain receptors | 3 | 2.752294 | 0.024786 |
| GOTERM_BP_DIRECT | GO:0006778~porphyrin-containing compound metabolic process | 2 | 1.834862 | 0.025482 |
| GOTERM_BP_DIRECT | GO:0061419~positive regulation of transcription from RNA polymerase II promoter in response to hypoxia | 2 | 1.834862 | 0.025482 |
| GOTERM_BP_DIRECT | GO:0045191~regulation of isotype switching | 2 | 1.834862 | 0.025482 |
| GOTERM_BP_DIRECT | GO:0071677~positive regulation of mononuclear cell migration | 2 | 1.834862 | 0.025482 |
| GOTERM_BP_DIRECT | GO:0003056~regulation of vascular smooth muscle contraction | 2 | 1.834862 | 0.025482 |
| GOTERM_BP_DIRECT | GO:0000320~re-entry into mitotic cell cycle | 2 | 1.834862 | 0.025482 |
| GOTERM_BP_DIRECT | GO:1900015~regulation of cytokine production involved in inflammatory response | 2 | 1.834862 | 0.025482 |
| GOTERM_BP_DIRECT | GO:0045986~negative regulation of smooth muscle contraction | 2 | 1.834862 | 0.025482 |
| GOTERM_BP_DIRECT | GO:0070849~response to epidermal growth factor | 2 | 1.834862 | 0.025482 |
| GOTERM_BP_DIRECT | GO:0071395~cellular response to jasmonic acid stimulus | 2 | 1.834862 | 0.025482 |
| GOTERM_BP_DIRECT | GO:0090170~regulation of Golgi inheritance | 2 | 1.834862 | 0.025482 |
| GOTERM_BP_DIRECT | GO:0002248~connective tissue replacement involved in inflammatory response wound healing | 2 | 1.834862 | 0.025482 |
| GOTERM_BP_DIRECT | GO:0008219~cell death | 3 | 2.752294 | 0.026018 |
| GOTERM_BP_DIRECT | GO:0006953~acute-phase response | 3 | 2.752294 | 0.026018 |
| GOTERM_BP_DIRECT | GO:0000302~response to reactive oxygen species | 3 | 2.752294 | 0.026018 |
| GOTERM_BP_DIRECT | GO:0016310~phosphorylation | 4 | 3.669725 | 0.02668 |
| GOTERM_BP_DIRECT | GO:0051262~protein tetramerization | 3 | 2.752294 | 0.027274 |
| GOTERM_BP_DIRECT | GO:0002576~platelet degranulation | 4 | 3.669725 | 0.028783 |
| GOTERM_BP_DIRECT | GO:0045740~positive regulation of DNA replication | 3 | 2.752294 | 0.029858 |
| GOTERM_BP_DIRECT | GO:0007596~blood coagulation | 5 | 4.587156 | 0.031014 |
| GOTERM_BP_DIRECT | GO:0051583~dopamine uptake involved in synaptic transmission | 2 | 1.834862 | 0.031751 |
| GOTERM_BP_DIRECT | GO:0042092~type 2 immune response | 2 | 1.834862 | 0.031751 |
| GOTERM_BP_DIRECT | GO:0010656~negative regulation of muscle cell apoptotic process | 2 | 1.834862 | 0.031751 |
| GOTERM_BP_DIRECT | GO:0051023~regulation of immunoglobulin secretion | 2 | 1.834862 | 0.031751 |
| GOTERM_BP_DIRECT | GO:0046541~saliva secretion | 2 | 1.834862 | 0.031751 |
| GOTERM_BP_DIRECT | GO:2001300~lipoxin metabolic process | 2 | 1.834862 | 0.031751 |
| GOTERM_BP_DIRECT | GO:0016101~diterpenoid metabolic process | 2 | 1.834862 | 0.031751 |
| GOTERM_BP_DIRECT | GO:0006706~steroid catabolic process | 2 | 1.834862 | 0.031751 |
| GOTERM_BP_DIRECT | GO:0042420~dopamine catabolic process | 2 | 1.834862 | 0.031751 |
| GOTERM_BP_DIRECT | GO:0032755~positive regulation of interleukin-6 production | 3 | 2.752294 | 0.033909 |
| GOTERM_BP_DIRECT | GO:0032147~activation of protein kinase activity | 3 | 2.752294 | 0.033909 |
| GOTERM_BP_DIRECT | GO:0032870~cellular response to hormone stimulus | 3 | 2.752294 | 0.033909 |
| GOTERM_BP_DIRECT | GO:0009615~response to virus | 4 | 3.669725 | 0.034021 |
| GOTERM_BP_DIRECT | GO:0071356~cellular response to tumor necrosis factor | 4 | 3.669725 | 0.034021 |
| GOTERM_BP_DIRECT | GO:0007187~G-protein coupled receptor signaling pathway, coupled to cyclic nucleotide second messenger | 3 | 2.752294 | 0.035304 |
| GOTERM_BP_DIRECT | GO:0003151~outflow tract morphogenesis | 3 | 2.752294 | 0.035304 |
| GOTERM_BP_DIRECT | GO:0031100~organ regeneration | 3 | 2.752294 | 0.036721 |
| GOTERM_BP_DIRECT | GO:0045600~positive regulation of fat cell differentiation | 3 | 2.752294 | 0.036721 |
| GOTERM_BP_DIRECT | GO:0042110~T cell activation | 3 | 2.752294 | 0.036721 |
| GOTERM_BP_DIRECT | GO:0051781~positive regulation of cell division | 3 | 2.752294 | 0.036721 |
| GOTERM_BP_DIRECT | GO:0071321~cellular response to cGMP | 2 | 1.834862 | 0.03798 |
| GOTERM_BP_DIRECT | GO:0002740~negative regulation of cytokine secretion involved in immune response | 2 | 1.834862 | 0.03798 |
| GOTERM_BP_DIRECT | GO:0016098~monoterpenoid metabolic process | 2 | 1.834862 | 0.03798 |
| GOTERM_BP_DIRECT | GO:0030816~positive regulation of cAMP metabolic process | 2 | 1.834862 | 0.03798 |
| GOTERM_BP_DIRECT | GO:0090336~positive regulation of brown fat cell differentiation | 2 | 1.834862 | 0.03798 |
| GOTERM_BP_DIRECT | GO:0042737~drug catabolic process | 2 | 1.834862 | 0.03798 |
| GOTERM_BP_DIRECT | GO:0002933~lipid hydroxylation | 2 | 1.834862 | 0.03798 |
| GOTERM_BP_DIRECT | GO:0045908~negative regulation of vasodilation | 2 | 1.834862 | 0.03798 |
| GOTERM_BP_DIRECT | GO:0060137~maternal process involved in parturition | 2 | 1.834862 | 0.03798 |
| GOTERM_BP_DIRECT | GO:0043619~regulation of transcription from RNA polymerase II promoter in response to oxidative stress | 2 | 1.834862 | 0.03798 |
| GOTERM_BP_DIRECT | GO:0001957~intramembranous ossification | 2 | 1.834862 | 0.03798 |
| GOTERM_BP_DIRECT | GO:0007253~cytoplasmic sequestering of NF-kappaB | 2 | 1.834862 | 0.03798 |
| GOTERM_BP_DIRECT | GO:0060440~trachea formation | 2 | 1.834862 | 0.03798 |
| GOTERM_BP_DIRECT | GO:2001171~positive regulation of ATP biosynthetic process | 2 | 1.834862 | 0.03798 |
| GOTERM_BP_DIRECT | GO:0042752~regulation of circadian rhythm | 3 | 2.752294 | 0.039621 |
| GOTERM_BP_DIRECT | GO:0071560~cellular response to transforming growth factor beta stimulus | 3 | 2.752294 | 0.039621 |
| GOTERM_BP_DIRECT | GO:0045892~negative regulation of transcription, DNA-templated | 8 | 7.33945 | 0.042089 |
| GOTERM_BP_DIRECT | GO:0071277~cellular response to calcium ion | 3 | 2.752294 | 0.042604 |
| GOTERM_BP_DIRECT | GO:0034097~response to cytokine | 3 | 2.752294 | 0.044126 |
| GOTERM_BP_DIRECT | GO:0090050~positive regulation of cell migration involved in sprouting angiogenesis | 2 | 1.834862 | 0.04417 |
| GOTERM_BP_DIRECT | GO:0043496~regulation of protein homodimerization activity | 2 | 1.834862 | 0.04417 |
| GOTERM_BP_DIRECT | GO:0040012~regulation of locomotion | 2 | 1.834862 | 0.04417 |
| GOTERM_BP_DIRECT | GO:0044321~response to leptin | 2 | 1.834862 | 0.04417 |
| GOTERM_BP_DIRECT | GO:0034351~negative regulation of glial cell apoptotic process | 2 | 1.834862 | 0.04417 |
| GOTERM_BP_DIRECT | GO:0071455~cellular response to hyperoxia | 2 | 1.834862 | 0.04417 |
| GOTERM_BP_DIRECT | GO:0015872~dopamine transport | 2 | 1.834862 | 0.04417 |
| GOTERM_BP_DIRECT | GO:0042167~heme catabolic process | 2 | 1.834862 | 0.04417 |
| GOTERM_BP_DIRECT | GO:0032287~peripheral nervous system myelin maintenance | 2 | 1.834862 | 0.04417 |
| GOTERM_BP_DIRECT | GO:0071639~positive regulation of monocyte chemotactic protein-1 production | 2 | 1.834862 | 0.04417 |
| GOTERM_BP_DIRECT | GO:0045727~positive regulation of translation | 3 | 2.752294 | 0.045668 |
| GOTERM_BP_DIRECT | GO:0051726~regulation of cell cycle | 4 | 3.669725 | 0.045872 |
| GOTERM_BP_DIRECT | GO:0048511~rhythmic process | 3 | 2.752294 | 0.04723 |
| GOTERM_BP_DIRECT | GO:0006366~transcription from RNA polymerase II promoter | 8 | 7.33945 | 0.047605 |
| GOTERM_BP_DIRECT | GO:0042981~regulation of apoptotic process | 5 | 4.587156 | 0.04869 |

## cell component

| Category | Term | Count | % | PValue |
| --- | --- | --- | --- | --- |
| GOTERM_CC_DIRECT | GO:0005615~extracellular space | 33 | 30.27523 | 4.79E-12 |
| GOTERM_CC_DIRECT | GO:0005901~caveola | 10 | 9.174312 | 1.56E-10 |
| GOTERM_CC_DIRECT | GO:0005886~plasma membrane | 49 | 44.95413 | 3.87E-07 |
| GOTERM_CC_DIRECT | GO:0005829~cytosol | 41 | 37.61468 | 2.77E-06 |
| GOTERM_CC_DIRECT | GO:0005576~extracellular region | 26 | 23.85321 | 6.00E-06 |
| GOTERM_CC_DIRECT | GO:0005887~integral component of plasma membrane | 24 | 22.01835 | 7.32E-06 |
| GOTERM_CC_DIRECT | GO:0045121~membrane raft | 9 | 8.256881 | 3.11E-05 |
| GOTERM_CC_DIRECT | GO:0045211~postsynaptic membrane | 9 | 8.256881 | 3.69E-05 |
| GOTERM_CC_DIRECT | GO:0045202~synapse | 8 | 7.33945 | 1.02E-04 |
| GOTERM_CC_DIRECT | GO:0043235~receptor complex | 7 | 6.422018 | 1.09E-04 |
| GOTERM_CC_DIRECT | GO:0031090~organelle membrane | 6 | 5.504587 | 1.67E-04 |
| GOTERM_CC_DIRECT | GO:0005739~mitochondrion | 20 | 18.34862 | 2.85E-04 |
| GOTERM_CC_DIRECT | GO:0005789~endoplasmic reticulum membrane | 15 | 13.76147 | 5.65E-04 |
| GOTERM_CC_DIRECT | GO:0070062~extracellular exosome | 31 | 28.44037 | 7.16E-04 |
| GOTERM_CC_DIRECT | GO:0043234~protein complex | 10 | 9.174312 | 7.69E-04 |
| GOTERM_CC_DIRECT | GO:0032279~asymmetric synapse | 3 | 2.752294 | 9.52E-04 |
| GOTERM_CC_DIRECT | GO:0009986~cell surface | 11 | 10.09174 | 0.001422 |
| GOTERM_CC_DIRECT | GO:0016324~apical plasma membrane | 8 | 7.33945 | 0.001739 |
| GOTERM_CC_DIRECT | GO:0043679~axon terminus | 4 | 3.669725 | 0.002884 |
| GOTERM_CC_DIRECT | GO:0005783~endoplasmic reticulum | 13 | 11.92661 | 0.003646 |
| GOTERM_CC_DIRECT | GO:0016323~basolateral plasma membrane | 6 | 5.504587 | 0.004369 |
| GOTERM_CC_DIRECT | GO:0032839~dendrite cytoplasm | 3 | 2.752294 | 0.005004 |
| GOTERM_CC_DIRECT | GO:0043231~intracellular membrane-bounded organelle | 10 | 9.174312 | 0.005927 |
| GOTERM_CC_DIRECT | GO:0098794~postsynapse | 3 | 2.752294 | 0.00744 |
| GOTERM_CC_DIRECT | GO:0031012~extracellular matrix | 7 | 6.422018 | 0.008389 |
| GOTERM_CC_DIRECT | GO:0005925~focal adhesion | 8 | 7.33945 | 0.008677 |
| GOTERM_CC_DIRECT | GO:0009897~external side of plasma membrane | 6 | 5.504587 | 0.008777 |
| GOTERM_CC_DIRECT | GO:0043025~neuronal cell body | 7 | 6.422018 | 0.011176 |
| GOTERM_CC_DIRECT | GO:0048471~perinuclear region of cytoplasm | 10 | 9.174312 | 0.011505 |
| GOTERM_CC_DIRECT | GO:0031965~nuclear membrane | 6 | 5.504587 | 0.01175 |
| GOTERM_CC_DIRECT | GO:0043005~neuron projection | 6 | 5.504587 | 0.013466 |
| GOTERM_CC_DIRECT | GO:0005635~nuclear envelope | 5 | 4.587156 | 0.014838 |
| GOTERM_CC_DIRECT | GO:0090575~RNA polymerase II transcription factor complex | 3 | 2.752294 | 0.016301 |
| GOTERM_CC_DIRECT | GO:0030054~cell junction | 8 | 7.33945 | 0.019435 |
| GOTERM_CC_DIRECT | GO:0016020~membrane | 21 | 19.26606 | 0.033194 |
| GOTERM_CC_DIRECT | GO:0005654~nucleoplasm | 25 | 22.93578 | 0.034766 |
| GOTERM_CC_DIRECT | GO:0030139~endocytic vesicle | 3 | 2.752294 | 0.04078 |
| GOTERM_CC_DIRECT | GO:0031093~platelet alpha granule lumen | 3 | 2.752294 | 0.042159 |
| GOTERM_CC_DIRECT | GO:0030424~axon | 5 | 4.587156 | 0.043114 |
| GOTERM_CC_DIRECT | GO:0030425~dendrite | 6 | 5.504587 | 0.049148 |
| GOTERM_CC_DIRECT | GO:0005622~intracellular | 14 | 12.84404 | 0.051293 |
| GOTERM_CC_DIRECT | GO:0046930~pore complex | 2 | 1.834862 | 0.0521 |
| GOTERM_CC_DIRECT | GO:0005741~mitochondrial outer membrane | 4 | 3.669725 | 0.058811 |
| GOTERM_CC_DIRECT | GO:0005634~nucleus | 41 | 37.61468 | 0.060894 |
| GOTERM_CC_DIRECT | GO:0030141~secretory granule | 3 | 2.752294 | 0.071374 |
| GOTERM_CC_DIRECT | GO:0005578~proteinaceous extracellular matrix | 5 | 4.587156 | 0.075195 |
| GOTERM_CC_DIRECT | GO:0031143~pseudopodium | 2 | 1.834862 | 0.096149 |
| GOTERM_CC_DIRECT | GO:0010008~endosome membrane | 4 | 3.669725 | 0.097376 |
