# Supplementary material for: Integrated Network Pharmacology and Gut Microbiota Analysis to Explore the Mechanism of Sijunzi Decoction Involved in Alleviating Airway Inflammation in a Mouse Model of Asthma
Source: Evid Based Complement Alternat Med. 2023 Jan 3;2023:1130893. doi: 10.1155/2023/1130893 (PMC9831717; doi:10.1155/2023/1130893)
Supplement: Supplementary Materials — Supplementary Table 1: active compounds from databases and literature in Sijunzi decoction. Supplementary Table 2: asthma-related genes in the database. Supplementary Table 3: common genes of asthma and Sijunzi decoction. Supplementary Table 4: GO functional categories. Supplementary Table 5: data of KEGG enrichment analysis. [file 1130893.f1.zip › Supplementary Table 5.docx]

**Supplementary Table 5: data of KEGG enrichment analysis.**

| Category | Term | Count | | % | PValue |
| --- | --- | --- | --- | --- | --- |
| KEGG_PATHWAY | hsa04066:HIF-1 signaling pathway | | 17 | 15.59633 | 3.50E-13 |
| KEGG_PATHWAY | hsa05200:Pathways in cancer | | 28 | 25.68807 | 5.96E-12 |
| KEGG_PATHWAY | hsa05219:Bladder cancer | | 12 | 11.00917 | 8.61E-12 |
| KEGG_PATHWAY | hsa05161:Hepatitis B | | 18 | 16.51376 | 2.20E-11 |
| KEGG_PATHWAY | hsa04668:TNF signaling pathway | | 16 | 14.6789 | 2.74E-11 |
| KEGG_PATHWAY | hsa05205:Proteoglycans in cancer | | 20 | 18.34862 | 5.49E-11 |
| KEGG_PATHWAY | hsa05212:Pancreatic cancer | | 13 | 11.92661 | 1.09E-10 |
| KEGG_PATHWAY | hsa05142:Chagas disease (American trypanosomiasis) | | 14 | 12.84404 | 2.73E-09 |
| KEGG_PATHWAY | hsa05213:Endometrial cancer | | 11 | 10.09174 | 2.81E-09 |
| KEGG_PATHWAY | hsa05215:Prostate cancer | | 13 | 11.92661 | 4.22E-09 |
| KEGG_PATHWAY | hsa04020:Calcium signaling pathway | | 17 | 15.59633 | 5.25E-09 |
| KEGG_PATHWAY | hsa05145:Toxoplasmosis | | 14 | 12.84404 | 5.50E-09 |
| KEGG_PATHWAY | hsa04068:FoxO signaling pathway | | 15 | 13.76147 | 6.94E-09 |
| KEGG_PATHWAY | hsa04071:Sphingolipid signaling pathway | | 14 | 12.84404 | 1.61E-08 |
| KEGG_PATHWAY | hsa05210:Colorectal cancer | | 11 | 10.09174 | 1.69E-08 |
| KEGG_PATHWAY | hsa04620:Toll-like receptor signaling pathway | | 13 | 11.92661 | 3.63E-08 |
| KEGG_PATHWAY | hsa04510:Focal adhesion | | 17 | 15.59633 | 3.96E-08 |
| KEGG_PATHWAY | hsa05160:Hepatitis C | | 14 | 12.84404 | 5.61E-08 |
| KEGG_PATHWAY | hsa05140:Leishmaniasis | | 11 | 10.09174 | 6.52E-08 |
| KEGG_PATHWAY | hsa04151:PI3K-Akt signaling pathway | | 21 | 19.26606 | 9.41E-08 |
| KEGG_PATHWAY | hsa05223:Non-small cell lung cancer | | 10 | 9.174312 | 9.46E-08 |
| KEGG_PATHWAY | hsa05133:Pertussis | | 11 | 10.09174 | 1.11E-07 |
| KEGG_PATHWAY | hsa04660:T cell receptor signaling pathway | | 12 | 11.00917 | 1.89E-07 |
| KEGG_PATHWAY | hsa05164:Influenza A | | 15 | 13.76147 | 1.96E-07 |
| KEGG_PATHWAY | hsa05230:Central carbon metabolism in cancer | | 10 | 9.174312 | 3.12E-07 |
| KEGG_PATHWAY | hsa05214:Glioma | | 10 | 9.174312 | 3.58E-07 |
| KEGG_PATHWAY | hsa05222:Small cell lung cancer | | 11 | 10.09174 | 3.73E-07 |
| KEGG_PATHWAY | hsa04080:Neuroactive ligand-receptor interaction | | 18 | 16.51376 | 4.45E-07 |
| KEGG_PATHWAY | hsa04012:ErbB signaling pathway | | 11 | 10.09174 | 4.65E-07 |
| KEGG_PATHWAY | hsa04725:Cholinergic synapse | | 12 | 11.00917 | 5.52E-07 |
| KEGG_PATHWAY | hsa04917:Prolactin signaling pathway | | 10 | 9.174312 | 7.75E-07 |
| KEGG_PATHWAY | hsa04621:NOD-like receptor signaling pathway | | 9 | 8.256881 | 1.30E-06 |
| KEGG_PATHWAY | hsa05169:Epstein-Barr virus infection | | 12 | 11.00917 | 1.43E-06 |
| KEGG_PATHWAY | hsa04915:Estrogen signaling pathway | | 11 | 10.09174 | 1.57E-06 |
| KEGG_PATHWAY | hsa04370:VEGF signaling pathway | | 9 | 8.256881 | 2.53E-06 |
| KEGG_PATHWAY | hsa04022:cGMP-PKG signaling pathway | | 13 | 11.92661 | 2.90E-06 |
| KEGG_PATHWAY | hsa04380:Osteoclast differentiation | | 12 | 11.00917 | 2.90E-06 |
| KEGG_PATHWAY | hsa04664:Fc epsilon RI signaling pathway | | 9 | 8.256881 | 5.84E-06 |
| KEGG_PATHWAY | hsa05014:Amyotrophic lateral sclerosis (ALS) | | 8 | 7.33945 | 7.11E-06 |
| KEGG_PATHWAY | hsa05218:Melanoma | | 9 | 8.256881 | 8.10E-06 |
| KEGG_PATHWAY | hsa05220:Chronic myeloid leukemia | | 9 | 8.256881 | 9.01E-06 |
| KEGG_PATHWAY | hsa04722:Neurotrophin signaling pathway | | 11 | 10.09174 | 9.04E-06 |
| KEGG_PATHWAY | hsa05231:Choline metabolism in cancer | | 10 | 9.174312 | 1.51E-05 |
| KEGG_PATHWAY | hsa04923:Regulation of lipolysis in adipocytes | | 8 | 7.33945 | 1.54E-05 |
| KEGG_PATHWAY | hsa05221:Acute myeloid leukemia | | 8 | 7.33945 | 1.54E-05 |
| KEGG_PATHWAY | hsa05206:MicroRNAs in cancer | | 16 | 14.6789 | 1.56E-05 |
| KEGG_PATHWAY | hsa04024:cAMP signaling pathway | | 13 | 11.92661 | 2.92E-05 |
| KEGG_PATHWAY | hsa04210:Apoptosis | | 8 | 7.33945 | 3.03E-05 |
| KEGG_PATHWAY | hsa04261:Adrenergic signaling in cardiomyocytes | | 11 | 10.09174 | 3.09E-05 |
| KEGG_PATHWAY | hsa04726:Serotonergic synapse | | 10 | 9.174312 | 3.24E-05 |
| KEGG_PATHWAY | hsa04919:Thyroid hormone signaling pathway | | 10 | 9.174312 | 4.30E-05 |
| KEGG_PATHWAY | hsa05216:Thyroid cancer | | 6 | 5.504587 | 5.55E-05 |
| KEGG_PATHWAY | hsa04930:Type II diabetes mellitus | | 7 | 6.422018 | 6.43E-05 |
| KEGG_PATHWAY | hsa04010:MAPK signaling pathway | | 14 | 12.84404 | 7.51E-05 |
| KEGG_PATHWAY | hsa05204:Chemical carcinogenesis | | 8 | 7.33945 | 1.58E-04 |
| KEGG_PATHWAY | hsa04931:Insulin resistance | | 9 | 8.256881 | 1.72E-04 |
| KEGG_PATHWAY | hsa04150:mTOR signaling pathway | | 7 | 6.422018 | 1.88E-04 |
| KEGG_PATHWAY | hsa00140:Steroid hormone biosynthesis | | 7 | 6.422018 | 1.88E-04 |
| KEGG_PATHWAY | hsa05132:Salmonella infection | | 8 | 7.33945 | 1.99E-04 |
| KEGG_PATHWAY | hsa04960:Aldosterone-regulated sodium reabsorption | | 6 | 5.504587 | 2.39E-04 |
| KEGG_PATHWAY | hsa04970:Salivary secretion | | 8 | 7.33945 | 2.48E-04 |
| KEGG_PATHWAY | hsa05152:Tuberculosis | | 11 | 10.09174 | 2.50E-04 |
| KEGG_PATHWAY | hsa04912:GnRH signaling pathway | | 8 | 7.33945 | 3.52E-04 |
| KEGG_PATHWAY | hsa05211:Renal cell carcinoma | | 7 | 6.422018 | 3.85E-04 |
| KEGG_PATHWAY | hsa04662:B cell receptor signaling pathway | | 7 | 6.422018 | 4.90E-04 |
| KEGG_PATHWAY | hsa04920:Adipocytokine signaling pathway | | 7 | 6.422018 | 5.29E-04 |
| KEGG_PATHWAY | hsa04913:Ovarian steroidogenesis | | 6 | 5.504587 | 7.06E-04 |
| KEGG_PATHWAY | hsa05162:Measles | | 9 | 8.256881 | 7.07E-04 |
| KEGG_PATHWAY | hsa00980:Metabolism of xenobiotics by cytochrome P450 | | 7 | 6.422018 | 7.14E-04 |
| KEGG_PATHWAY | hsa05020:Prion diseases | | 5 | 4.587156 | 0.001448 |
| KEGG_PATHWAY | hsa04062:Chemokine signaling pathway | | 10 | 9.174312 | 0.001561 |
| KEGG_PATHWAY | hsa04932:Non-alcoholic fatty liver disease (NAFLD) | | 9 | 8.256881 | 0.001613 |
| KEGG_PATHWAY | hsa04914:Progesterone-mediated oocyte maturation | | 7 | 6.422018 | 0.001669 |
| KEGG_PATHWAY | hsa04064:NF-kappa B signaling pathway | | 7 | 6.422018 | 0.001669 |
| KEGG_PATHWAY | hsa04014:Ras signaling pathway | | 11 | 10.09174 | 0.001683 |
| KEGG_PATHWAY | hsa04540:Gap junction | | 7 | 6.422018 | 0.00177 |
| KEGG_PATHWAY | hsa05323:Rheumatoid arthritis | | 7 | 6.422018 | 0.00177 |
| KEGG_PATHWAY | hsa04152:AMPK signaling pathway | | 8 | 7.33945 | 0.00211 |
| KEGG_PATHWAY | hsa05131:Shigellosis | | 6 | 5.504587 | 0.002373 |
| KEGG_PATHWAY | hsa05321:Inflammatory bowel disease (IBD) | | 6 | 5.504587 | 0.002373 |
| KEGG_PATHWAY | hsa00380:Tryptophan metabolism | | 5 | 4.587156 | 0.002667 |
| KEGG_PATHWAY | hsa04611:Platelet activation | | 8 | 7.33945 | 0.002888 |
| KEGG_PATHWAY | hsa04115:p53 signaling pathway | | 6 | 5.504587 | 0.002903 |
| KEGG_PATHWAY | hsa05120:Epithelial cell signaling in Helicobacter pylori infection | | 6 | 5.504587 | 0.002903 |
| KEGG_PATHWAY | hsa04622:RIG-I-like receptor signaling pathway | | 6 | 5.504587 | 0.003515 |
| KEGG_PATHWAY | hsa04723:Retrograde endocannabinoid signaling | | 7 | 6.422018 | 0.003553 |
| KEGG_PATHWAY | hsa04810:Regulation of actin cytoskeleton | | 10 | 9.174312 | 0.003562 |
| KEGG_PATHWAY | hsa04015:Rap1 signaling pathway | | 10 | 9.174312 | 0.003562 |
| KEGG_PATHWAY | hsa05144:Malaria | | 5 | 4.587156 | 0.005592 |
| KEGG_PATHWAY | hsa04921:Oxytocin signaling pathway | | 8 | 7.33945 | 0.00634 |
| KEGG_PATHWAY | hsa04270:Vascular smooth muscle contraction | | 7 | 6.422018 | 0.007269 |
| KEGG_PATHWAY | hsa05310:Asthma | | 4 | 3.669725 | 0.009356 |
| KEGG_PATHWAY | hsa05203:Viral carcinogenesis | | 9 | 8.256881 | 0.010246 |
| KEGG_PATHWAY | hsa05202:Transcriptional misregulation in cancer | | 8 | 7.33945 | 0.011152 |
| KEGG_PATHWAY | hsa05166:HTLV-I infection | | 10 | 9.174312 | 0.011975 |
| KEGG_PATHWAY | hsa00590:Arachidonic acid metabolism | | 5 | 4.587156 | 0.012051 |
| KEGG_PATHWAY | hsa05143:African trypanosomiasis | | 4 | 3.669725 | 0.012181 |
| KEGG_PATHWAY | hsa04924:Renin secretion | | 5 | 4.587156 | 0.014193 |
| KEGG_PATHWAY | hsa05330:Allograft rejection | | 4 | 3.669725 | 0.016632 |
| KEGG_PATHWAY | hsa04550:Signaling pathways regulating pluripotency of stem cells | | 7 | 6.422018 | 0.016671 |
| KEGG_PATHWAY | hsa00982:Drug metabolism - cytochrome P450 | | 5 | 4.587156 | 0.017403 |
| KEGG_PATHWAY | hsa05146:Amoebiasis | | 6 | 5.504587 | 0.019482 |
| KEGG_PATHWAY | hsa04630:Jak-STAT signaling pathway | | 7 | 6.422018 | 0.019489 |
| KEGG_PATHWAY | hsa04520:Adherens junction | | 5 | 4.587156 | 0.020086 |
| KEGG_PATHWAY | hsa04666:Fc gamma R-mediated phagocytosis | | 5 | 4.587156 | 0.034568 |
| KEGG_PATHWAY | hsa04728:Dopaminergic synapse | | 6 | 5.504587 | 0.039622 |
| KEGG_PATHWAY | hsa04910:Insulin signaling pathway | | 6 | 5.504587 | 0.051855 |
| KEGG_PATHWAY | hsa04310:Wnt signaling pathway | | 6 | 5.504587 | 0.051855 |
| KEGG_PATHWAY | hsa05168:Herpes simplex infection | | 7 | 6.422018 | 0.05187 |
| KEGG_PATHWAY | hsa04750:Inflammatory mediator regulation of TRP channels | | 5 | 4.587156 | 0.055528 |
| KEGG_PATHWAY | hsa04320:Dorso-ventral axis formation | | 3 | 2.752294 | 0.059069 |
| KEGG_PATHWAY | hsa04060:Cytokine-cytokine receptor interaction | | 8 | 7.33945 | 0.06567 |
| KEGG_PATHWAY | hsa00830:Retinol metabolism | | 4 | 3.669725 | 0.067325 |
| KEGG_PATHWAY | hsa04623:Cytosolic DNA-sensing pathway | | 4 | 3.669725 | 0.067325 |
| KEGG_PATHWAY | hsa05031:Amphetamine addiction | | 4 | 3.669725 | 0.072435 |
| KEGG_PATHWAY | hsa04610:Complement and coagulation cascades | | 4 | 3.669725 | 0.080417 |
| KEGG_PATHWAY | hsa04670:Leukocyte transendothelial migration | | 5 | 4.587156 | 0.088374 |
